# The best practice for microbiome analysis using R

**DOI:** 10.1093/procel/pwad024

**Published:** 2023-05-02

**Authors:** Tao Wen, Guoqing Niu, Tong Chen, Qirong Shen, Jun Yuan, Yong-Xin Liu

**Affiliations:** Shenzhen Branch, Guangdong Laboratory of Lingnan Modern Agriculture, Genome Analysis Laboratory of the Ministry of Agriculture and Rural Affairs, Agricultural Genomics Institute at Shenzhen, Chinese Academy of Agricultural Sciences, Shenzhen 518120, China; The Key Laboratory of Plant Immunity Jiangsu Provincial Key Lab for Organic Solid Waste Utilization Jiangsu Collaborative Innovation Center for Solid Organic Waste Resource Utilization, National Engineering Research Center for Organic-based Fertilizers, Nanjing Agricultural University, Nanjing 210095, China; The Key Laboratory of Plant Immunity Jiangsu Provincial Key Lab for Organic Solid Waste Utilization Jiangsu Collaborative Innovation Center for Solid Organic Waste Resource Utilization, National Engineering Research Center for Organic-based Fertilizers, Nanjing Agricultural University, Nanjing 210095, China; National Resource Center for Chinese Materia Medica, China Academy of Chinese Medical Sciences, Beijing 100700, China; The Key Laboratory of Plant Immunity Jiangsu Provincial Key Lab for Organic Solid Waste Utilization Jiangsu Collaborative Innovation Center for Solid Organic Waste Resource Utilization, National Engineering Research Center for Organic-based Fertilizers, Nanjing Agricultural University, Nanjing 210095, China; The Key Laboratory of Plant Immunity Jiangsu Provincial Key Lab for Organic Solid Waste Utilization Jiangsu Collaborative Innovation Center for Solid Organic Waste Resource Utilization, National Engineering Research Center for Organic-based Fertilizers, Nanjing Agricultural University, Nanjing 210095, China; Shenzhen Branch, Guangdong Laboratory of Lingnan Modern Agriculture, Genome Analysis Laboratory of the Ministry of Agriculture and Rural Affairs, Agricultural Genomics Institute at Shenzhen, Chinese Academy of Agricultural Sciences, Shenzhen 518120, China

**Keywords:** R package, microbiome, data analysis, visualization, amplicon, metagenome

## Abstract

With the gradual maturity of sequencing technology, many microbiome studies have published, driving the emergence and advance of related analysis tools. R language is the widely used platform for microbiome data analysis for powerful functions. However, tens of thousands of R packages and numerous similar analysis tools have brought major challenges for many researchers to explore microbiome data. How to choose suitable, efficient, convenient, and easy-to-learn tools from the numerous R packages has become a problem for many microbiome researchers. We have organized 324 common R packages for microbiome analysis and classified them according to application categories (diversity, difference, biomarker, correlation and network, functional prediction, and others), which could help researchers quickly find relevant R packages for microbiome analysis. Furthermore, we systematically sorted the integrated R packages (phyloseq, microbiome, MicrobiomeAnalystR, Animalcules, microeco, and amplicon) for microbiome analysis, and summarized the advantages and limitations, which will help researchers choose the appropriate tools. Finally, we thoroughly reviewed the R packages for microbiome analysis, summarized most of the common analysis content in the microbiome, and formed the most suitable pipeline for microbiome analysis. This paper is accompanied by hundreds of examples with 10,000 lines codes in GitHub, which can help beginners to learn, also help analysts compare and test different tools. This paper systematically sorts the application of R in microbiome, providing an important theoretical basis and practical reference for the development of better microbiome tools in the future. All the code is available at GitHub github.com/taowenmicro/EasyMicrobiomeR.

## Graphical Abstract

**Graphical Abstract:**
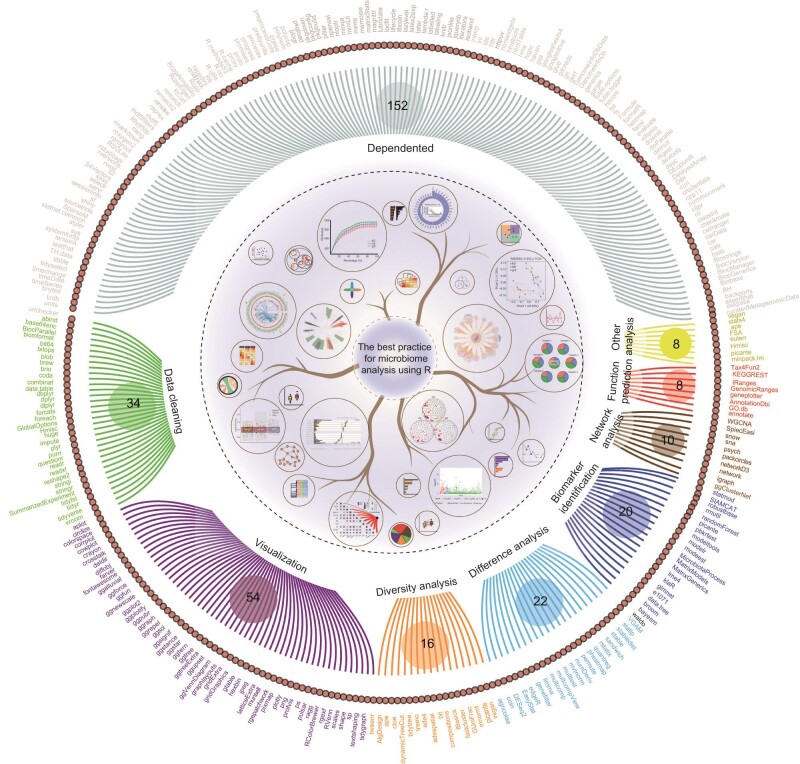

## Introduction

**Figure 1.**
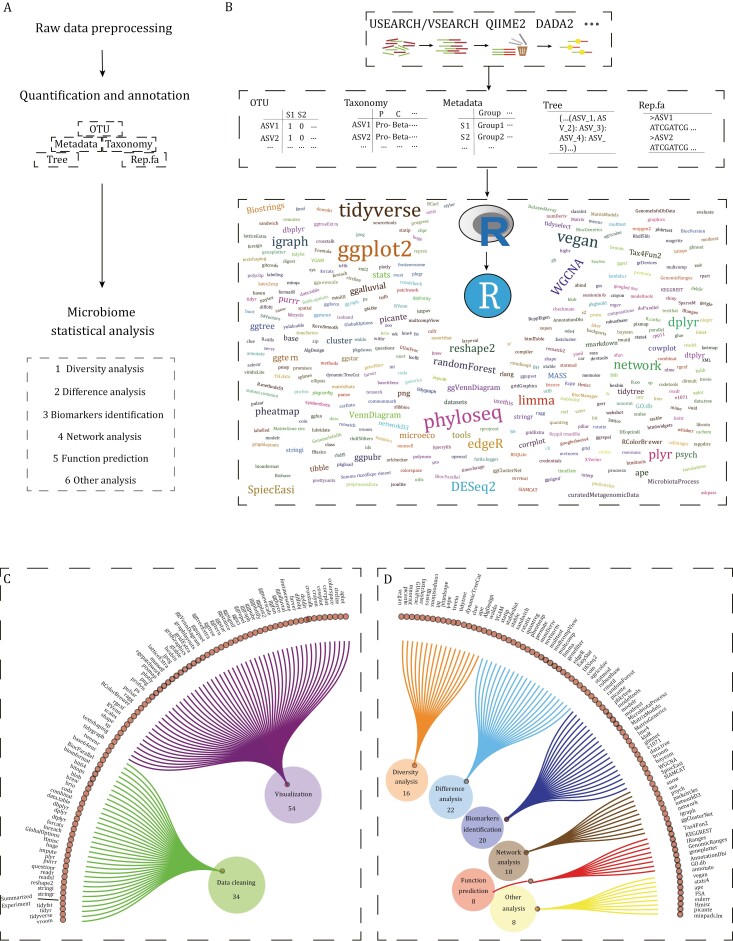
**Microbial community data analysis workflow and related R packages.** (A) Overview of microbial community data analysis workflow. Core files are feature table (OTU), Taxonomy, sample metadata (Metadata), phylogenetic tree (Tree), and representative sequences (Rep.fa). (B) Detail of microbial community analysis workflow. First, the raw data can be processed by using USEARCH/VSEARCH, QIIME 2, DADA2 packages. Then, the important files are saved and used for downstream analysis in R language and RStudio software. Many microbial analysis methods rely on numerous R packages developed with R language. The font size in the word cloud represents the number of citations of R packages. (C) Commonly used R packages for data-cleaning/manipulation and visualization. (D) Classification of R packages for six categories in microbial community analysis.

The metagenomic analysis is used to study microbial diversity, structure, and function by sequencing, quantifying, annotating, and analyzing DNA and/or RNA sequences of microbial communities or microbiota. The commonly used high-throughput sequencing technology in microbiome research is mainly known as amplicon sequencing and shotgun metagenomic sequencing. Amplicon sequencing with the advantages of low cost, mature analysis system, and simple analysis process was widely used in microbiome research. Shotgun metagenomic sequencing provided the functional information of microbes and more accurate information on the microbial composition with the higher sequencing cost and large amount of computational resources needed. The detailed pipeline for both sequencing methods have been systemically summarized in our previous review (Liu et al., 2021). As an important component of biodiversity, microbial communities play a vital role in biology, ecology, biotechnology, agriculture, and medicine. Various bioinformatics methods are required for microbial community analysis, which mainly includes three parts: (i) data preprocessing, (ii) quantification and annotation, and (iii) statistics and visualization (Fig. 1A). In the preprocessing step, the raw data is filtered and quality controlled to ensure data quality. In the quantification and annotation step, tools, and databases are used to identify microbial representative sequences and annotate microbial taxonomy and function. The first two parts of microbial community analysis have been well discussed and could be well done according to our previous paper (Liu et al., 2023). Finally, in the statistics and visualization step, various statistical methods are used to explore microbial community diversity, structure, and potential functions.

With the development of high-throughput sequencing technology, plenty of studies were performed with amplicon-sequencing technology (Thompson et al., 2017; Proctor et al., 2019) and shotgun metagenomes sequencing (Carrión et al., 2019; Li et al., 2022; Paoli et al., 2022), which led to the development of microbiome analysis methodologies, software, and pipelines, for example, QIIME (Caporaso et al., 2010), Mothur (Schloss et al., 2009), USEARCH (Edgar, 2010), VSEARCH (Rognes et al., 2016), QIIME 2 (Bolyen et al., 2019), Parallel-Meta Suite (Chen et al., 2022), EasyAmplicon (Liu et al., 2023), Kraken (Wood and Salzberg, 2014), MEGAN (Huson et al., 2007), MetaPhlAn2 (Truong et al., 2015), HUMAnN2 (Franzosa et al., 2018), etc. As the most crucial and basic procedure for amplicon sequencing data analysis, OTU (Operational taxonomic unit) clustering method was popular before the year of 2015 while non-clustering methods were gradually developed and widely used recently. Currently, the common non-clustering methods include DADA2 (Callahan et al., 2016), deblur (Amir et al., 2017), unoise3 (Edgar and Flyvbjerg, 2015). One of the most representative non-clustering algorithms among them is DADA2, which was created with R language. It makes the R language (Ihaka and Gentleman, 1996) occupy an important position in raw data processing for amplicon sequencing. Compared with many software that can be used in upstream steps of microbiota sequencing data analysis, the downstream analysis steps rely on the R language heavily with various packages. These analyses mainly include: (i) Diversity analysis; (ii) Difference analysis; (iii) Correlation and network analysis; (iv) Biomarker identification; (v) Functional predictions; (vi) Integrative analysis of microbial communities with other indicators (including phylogenetic analysis, multi-omics integration, environmental factor analysis, etc.). In addition to the kinds of multivariate statistical analysis that can be done in R, there are diversified data-cleaning packages that allow data to be transformed among different analyses.

R is a free, open-source language and environment for data statistical analysis and visualization, which was created by Ross Ihaka and Robert Gentleman from the University of Auckland in New Zealand and now is responsible by the “R Development Core Team”. Compared with other analysis tools, such as SPSS, MINITAB, MATLAB, which are more suitable for the statistics of processed and standardized data, R language can handle processed data as well as raw data. R can easily implement almost all analysis methods, many of the latest methods or algorithms were first exhibited in it. Furthermore, R shows excellent data visualization, particularly for complex data. The powerful and flexible interactive analysis is also an advantage of R, meanwhile enabling visual data exploration. The functionality of the R language relies heavily on thousands of R packages, which provide a wide variety of data processing and analysis strategies, allowing almost any data analysis process to be done in R. The total number of R packages published on CRAN is 18,981, and Bioconductor is 2,183 (by January 31, 2023). These packages demonstrated the powerful data process and analysis performance of R.

In recent years, numerous R packages have been developed on the R platform for the downstream analysis of microbiome, which have made important contributions to the associated-research field. However, the increasing number of downstream analysis R packages has reached a dizzying level (Fig. 1B). In addition, integrated R packages containing a large amount of microbiome analysis content, such as phyloseq (McMurdie and Holmes, 2013), microeco (Liu et al., 2020), and amplicon (Liu et al., 2023), have gradually emerged. This abundance of R packages provides microbiome analysts with more choices, but also makes it difficult to identify the most suitable tools among many similar analysis tools. Furthermore, this plethora of R packages make it difficult for beginners to embark on a well-organized learning path for microbiome analysis. Therefore, it is urgent to compare similar analysis functions, and extract the similarities and differences functions, to select the best process for microbiome analysis and help beginners learn more effectively.

This paper attempts to sort and run the 324 common R packages (Fig. S1), especially the integrated R packages for microbiome analysis, and complete the following three parts: (i) compare different R package analysis processes according to the functional categories of microbiome analysis, analyze the results, and summarize example code; (ii) organize the content of six integrated R packages according to the functional categories of microbiome analysis, compare the analysis results, and generate example code; (iii) based on all R packages, select the optimal analysis approach using R language and provide example code for reference and learning to researchers.

## Preparing microbiome data analysis

Downstream analysis of microbiome requires the preparation of five data files, including a feature table, a feature annotation file, a sample metadata file, a phylogenetic tree, and representative sequences. For beginners, it is important to understand the format and basic data structure of these files and learn how to import these files into R language. Furthermore, different analytical contents often have different requirements for data, and it is necessary to learn some data manipulation skills to meet the demands of various functions. Finally, it is necessary to learn the basics of R plotting to facilitate the presentation of results.

### Data preparation and cleaning

After the process of sequence data preprocessing, quantification, and annotation, we need to further analysis the output files, including importing these files, cleaning data, and converting format, which required for subsequent microbiome analysis in R. Before statistical analysis, we must master the basic procedure of R language to cope with the data input requirements of different packages. This section includes: importing, organizing, filtering, basic calculations, conversion, normalization, and modification of data. Five data forms are frequently used from raw data processing, including feature tables (file formats are .csv/.txt/.xlsx/.biom, typically used taxonomic and functional tables, including OTU/ASV/taxonomy/gene/module/pathway tables), feature annotation (.csv/.txt/.xlsx/.biom), sample metadata (.csv/.txt), evolutionary/phylogenetic trees (.nwk/.tree), representative sequences (.fasta/.fas/.fa). All the data cleaning-related packages show in Fig. 1C. Tabular data input for microbial community is primarily accomplished using functions such as *read.table*(), *read.delim*(), and *read.csv*() in the utils package (Code 1A, script in GitHub github.com/taowenmicro/EasyMicrobiomeR). The reading of evolutionary tree files depends on functions like *read.tree*() in the ape/ggtree/treeio package, or *read_tree*() in the phyloseq package. For reading representative sequence files in microbiome, the *readDNAStringSet*() in the Biostrings package (Pages et al., 2016) is typically used. Currently, big data integration of microbiome has become a trend, and leading to the emergence of R packages for integrated data from multiple studies, likes curatedMetagenomicData (Pasolli et al., 2017). The package only needs to import the package and could re-analysis the curated data, rather than input in raw sequencing data.

The basic idea of data organization can be summarized as three steps: splitting the data, processing with functions, and combining the output results into the desired format. The functions of basic packages in R can be combined to meet most requirements of the microbiome data operations. For example, the “for loop” combined with the basic statistical functions [*sum*(), *mean*(), *sd*(), etc.] can be used to perform basic statistical analysis and data transformations for microbial relative abundance (Code 1B); the base package provides the apply family of functions, including *apply*(), *sapply*(), *lapply*(), *tapply*(), *aggregate*(), etc., which can be applied to quickly complete the three stages of data processing. The apply family of functions provides a framework that acts as an alternative to “for loop” and is much faster than the basic “for loop” function in R (Code 1B). A similar purr package can be used in place of “for loop” to perform efficient operations.

The plyr (Wickham, 2011b) package was upgraded from package of base with a variety of data sorting processes for kinds of data frames, lists, etc. The plyr package provides three data processing stages “Split–Apply–Combine” in one function, and the plyr package implements grouping transformations between R types (vector, list, and data frame) and basically replaces the apply family of functions in the base package. It can easily handle grouping calculations, for example, microbial abundance at different taxonomy levels (Code 1C). The reshape2 (Wickham, 2007) package provides the long-wide format transformation during data processing, and since ggplot2 (Wickham, 2011a) plotting functions and most modeling functions, such as *lm*(), *glm*(), *gam*(), often use long data, microbiome data are general showed as wide form, so the transformation of microbiome data for plotting can be done using reshape2 (Code 1D), which provides the long-wide format transformation during data processing.

The dplyr package is a member of the tidyverse family, innovatively abandoning the common form of data preservation in R rather than using the tibble format (more powerful than data.frame format) for data processing, which can more efficiently complete the data frame selection, merging and statistics within row and column, and data frame length and width format changes, the “%>%” pipeline symbol can be used to complete more complex data processing. The tibble format can store data during the analysis and modeling process, which is important for data analysis. For example, we demonstrated the use of dplyr and pipeline to run random forest modeling and the selection process of important variables (Code 1E).

### Visualization in R language

In most cases, we are used to plotting standard graphs in microbiome data display such as alpha/beta diversity, taxonomic composition. All the visualization-related packages show in Fig. 1C. Due to the widespread use of ggplot2 (Code 2A), many extension packages have emerged to extend based on ggplot2 with a high capacity of plotting styles, colors, and themes. These packages mainly include ggtern plotting ternary graphs in Code 2B (Hamilton and Ferry, 2018), ggraph plotting network graphs in Code 2C (Si et al., 2022), ggtree plotting evolutionary tree or cladogram in Code 2D (Xu et al., 2022), the ggalluvial package, the ggVennDiagram package (Code 2E), the ggstatsplot package plotting pie chart, and the ggpubr package providing many various themes and colors of output. In addition, the pheatmap and ComplexHeatmap package (Gu, 2022) based on the grid mapping system plots the relative abundance of features in different samples (Code 2F), the VennDiagram package (Chen and Boutros, 2011) could show the number of features in different samples. The UpSetR package (Conway et al., 2017), which draws Upset view is a new form plotting similar to Venn diagram. The base-based plotting system is complex and difficult to learn, while it is a good choice for complex graph drawing, such as the circlize (Gu et al., 2014) package (Code 2G), which draws chord diagrams composed of microbiota.

Additionally, there is often a lot of microbiome mapping work that involves a combination of graphics. At present, many tools in R can combine graphics, such as cowplot, patchwork, and aplot. The patchwork package has the most powerful functions and supports modular splicing graphics (Code 2H).

## Microbial community analysis

We have categorized the analysis of microbiome data into the following six major types in Fig. 1D: diversity analysis, difference analysis, biomarkers identification, correlation and network analysis, functional prediction, and other microbiome analyses (including source tracking analysis, community assembly processes, and analysis of associations between microbiota and environmental factors). Then, we would have organized, compared, and summarized all relevant R packages.

### Diversity analysis

Microbial community diversity mainly includes alpha diversity (Richness, Shannon, Simpson, Chao1, ACE, etc.), rarefaction curve, beta diversity (ordination and clustering analysis), taxonomic or functional composition. Here must introduce the package vegan (Oksanen et al., 2007), an abbreviation for Vegetation Analysis, written by nine quantitative ecologists, including Oksanen from Finland, which is initially used for specifical dealing with data on community ecology. The package provides a variety of methods for data standardization and transformation. For example, data used for alpha diversity analysis can be normalized at the same sequencing depth with *rrarefy*(), and data for ordination analysis can be normalized with the *decostant*() (Code 3A). After the sequencing data are sampling normalization, diversity calculation can be more reasonable. In addition, alpha diversity metrics calculation can also be carried out with the ade4 (Dray and Dufour, 2007), adespatial (Dray et al., 2018), and picante packages (Kembel et al., 2010). For example, phylogenetic diversity can be calculated using the *pd*() in the picante package (Code 3A). Vegan not only allows for alpha diversity analysis, but also provides functions such as *rda*() for conducting principal components analysis (PCA) and redundancy analysis (RDA), *cca*() for conducting correspondence analysis (CA) and canonical correspondence analysis (CCA), *decorana*() for conducting decision curve analysis (DCA), and *metaMDS*() for conducting non-metric multidimensional scaling (NMDS) for microbiome ordination analysis (Code 3B). The *prcom*() in stats package can be used for principal component analysis (PCA), which is a kind of dimension reduction analysis. The *mca*() provided by the MASS package and the *MCA*() provided by the FactoMineR package can be used for multiple CA (Code 3B); the ape package provides the *pcoa*() function for principal coordinate analysis (PCoA); the MASS package provides *lda*() for linear discriminant analysis (LDA, Code 3C). Before running many ordination operations, it is often necessary for community clustering. The *vegdist*() in the vegan package can calculate Euclidean, Manhattan, Bray, Canberra, and other distances (Code 3B). In addition, distance calculation can also be done using *dist*() of stats package. The distance matrix can be used for clustering analysis in addition to ordination analysis. The *hclust*() in the stats package can be used for clustering analysis, a similar function can be achieved with the facteoextra, kmeans packages (Code 3D). Microbial composition analysis mainly used to display the abundance of microbes, and the dplyr package is needed to organize the data then display with ggplot2 subsequently.

### Difference analysis

Difference analysis is divided into community-level analysis and feature-level (any hierarchy of taxonomy and function) analysis. Community-level difference analysis is mainly performed with functions including *adonis*(), *anosim*(), and *mrpp*() in vegan package, and *mantel.test*() in ape package (Code 4A). The R package for compositional data difference analysis in the feature level can utilize the *wilcox.test*() (Code 4B) and *t.test*() (Code 4C) in the stats package. Subsequently, data correction algorithms were developed specifically for sequencing data, such as the upper quartile (UQ), trimmed mean of *M*-values (TMM) (Code 4C), and relative log expression (RLE) harbored in the edgeR package (Robinson et al., 2009) (Code 4D). Median of ratios method (MED) in DESeq2 package (Love et al., 2014) (Code 4E), and cumulative-sum scaling (CSS) algorithm in metagenomeSeq package (Code 4F). Furthermore, the ALDEx2 package provides polynomial models which can be used to infer feature abundance and calculate feature differences with non-parametric tests, *t*-tests, or generalized linear models (Code 4G). The ANCOM-BC package attempts to address sample heterogeneity by correcting bias with a log-linear model. In addition, other R packages for microbiome data correction and difference tests include limma (Code 4H), DR, ANCOM (Lin and Peddada, 2020) (Code 4I), corncob (Code 4J), Maaslin2 (Code 4K), etc. Nearing et al. (2022) showed that they compared these difference analysis methods and proposed that ALDEx2 and ANCOM-II (anchom_v2.1.R, Code 4L) were the best performers in the difference analysis of microbial communities. As for the significance test, different packages use different methods for significance testing. For example, Fisher test was used in edgeR package; Wald test was used in DESeq2 and corncob package; *t*-test was used in limma package. There were other methods for significance test, likes Wilcoxon rank-sum test (ALDEx2 and ANCOM-II), ANOVA (Maaslin2) etc.

### Biomarker identification

Characteristic microbial consortia were explored to explain certain questions, such as the biomarkers of the gut in obese or hypertensive populations, or of soil in Fusarium wilt develops, etc. Microbes selected through difference analysis are often unable to determine whether they represent the main differences of concern. Therefore, weight analysis or machine learning methods are used to further distinguish the feature microbes.

The main ones commonly used for weighted analysis are linear discriminant analysis effect size (LEfSe), PCA, etc (Code 5A). LEfSe is developed specifically for microbiome data, and the core functionality is implemented using the packages LDA (Fisher, 1936) and MASS (Ripley et al., 2013). By extracting the loading matrix of PCA ordination, the microbiome with the greatest impact on the sample variation are found as biomarkers (Code 5B).

In terms of machine learning, the random forest model, which is widely used in microbiome analysis, is implemented by using the randomforest package (Liaw and Wiener, 2002) (Code 5C). There are many other decision tree-based machine learning models, such as the mboost (Hofner et al., 2014) package provides boosting-based algorithms, the e1071 (Dimitriadou et al., 2008) package provides support vector machines *svm*() in Code 5D, and plain Bayes *naiveBayes*(). The xgboost package can integrate many tree models together to form a strong classifier, which can prevent overfitting via many strategies, including regularization terms, shrinkage, and column subsampling, etc. In addition, the pROC (Robin et al., 2011) package is used to plot the operating characteristic curve (ROC, Code 5D) to evaluate the efficiency of machine learning models. The Caret package provides cross-validation to determine the number of features (Kuhn, 2008). Currently, Wirbel et al. (2021) developed an open-source R package SIAMCAT, a powerful yet user-friendly computational machine learning toolkit tailored to the characteristics of microbiome data.

### Correlation and network analysis

Microbial co-occurrence network analysis is used to find microbial modules that may have mutualistic relationships. Co-occurrence network analysis mainly includes the calculation of correlations, network visualization, and the calculation of network properties. The common R packages for calculation of correlations are psych (Revelle and Revelle, 2015) (Code 6A), WGCNA (Langfelder and Horvath, 2008) (Code 6B), Hmisc (Harrell Jr and Harrell Jr, 2019) (Code 6C), and SpiecEasi (Kurtz et al., 2015) (Code 6D). Among these R packages, WGCNA has the highest calculation speed, while requiring additional *P*-value correction; psych can calculate correlation with correct *P*-value, but the speed is very low; the SpiecEasi package can use the sparcc method to perform a more suitable method for microbiome data to calculate the correlation matrix, and can call multiple-threads to accelerate the calculation. R packages for network visualization and attribute calculation can use igraph (Code 6E), network, and ggraph packages (Code 6F). These R packages contain many layout algorithms for network visualization. In addition, network packages combined with ggplot2 to visualize the network are easier to modify. Sna and ggraph packages have many visualization layout algorithms to increase the styles of network visualization. With the increasing use of network analysis in the microbiome analysis, more attention is paid to network modularity and the key groups through network modules. The WGCNA package provides a complete framework to quickly complete the correlation calculation, network module calculation, module feature vector calculation, and other network properties exploration. The recent development of the ggClusterNet (Wen et al., 2022) package (Code 6G) provides a unified framework for microbiome networks and designs a variety of unique module-based visualization algorithms to visualize the module relationships in the network.

### Functional prediction

The Tax4Fun (Aßhauer et al., 2015) R package (Code 7A) for functional prediction of 16S rDNA has been developed to more accurately predict changes in microbial community function using amplicon data. The package has been updated to Tax4Fun2 (Wemheuer et al., 2020). Microeco can implement FAPROTAX (Louca et al., 2016) prediction for bacteria/archaea and FUNGuild (Nguyen et al., 2016) prediction for fungi, which is based on the database of taxonomic functional description from curated published papers. Functional prediction enables the prediction of microbial community function and subsequent statistical analysis. Additionally, vegan can be used for diversity analysis, while edgeR, DEseq2, and limma packages can be used for difference analysis. For functional enrichment, the clusterProfiler (Code 7B) package can perform GO, KEGG, GSEA and GSVA enrichment, which considers gene/pathway abundance and is recommended. Furthermore, the clusterProfiler package provides plot functions based on the ggplot syntax, allowing to plot appealing graphics in a simple manner. Gene/Pathway network analysis can be performed using WGCNA for calculation, and ggClusterNet for network parameter calculation and visualization. However, the reliability of functional prediction results, particularly for environmental samples, is currently disputed, and therefore, further verification of analysis results is often required.

### Other microbiome analysis

Analysis for microbial community formation process commonly used in the framework proposed by Stegen et al. (2013) to calculate βNTI and RC-Bray indices with R packages minpack.lm, picante, Hmisc, eulerr, FSA, ape, stats4, and others (Code 8A). Ning et al. (2020) used a phylogenetic binning-based null model analysis to infer quantitative mechanisms underlying community assembly, and developed the R package iCAMP (Code 8B). It allows for the quantitative assessment of the relative importance of different ecological processes (e.g., homogenizing selection, heterogenizing selection, dispersal, and drift) on both the entire community and each phylogenetic bin (which is usually composed of taxa from a single family or order with distinct ecological characteristics). In addition, the package also provides neutral theory models, phylogenetic and taxonomic null model analyses at both the community and clade levels, calculation of niche differences and phylogenetic distances between clades, and tests for phylogenetic signals within individual phylogenetic bins.

Microbial communities were often used to analyze the correlation with environment indicators, for example, *mantel.test*() provided by the vegan package was used to examine the correlation between microbial communities and environment indicators, and using *wascores*(), *mantel.correlog*() to detect the phylogenetic signal between microbial communities and environmental factors (Code 8C). In addition, the ggClusterNet package can be used to calculate the co-occurrence relationships between microbes/microbiome and environmental factors, and generated publish-ready figures (Code 8D).

Knights et al. (2011) proposed the microbiome traceability tool source tracker in R language. Metcalf et al. (2016) predicted the time of death and tracked the source microbes of real cadavers on microbial communities, then microbial traceability analysis gradually popular. Shenhav et al. (2019) proposed a new algorithm in R, FEAST (Code 8E), which makes microbial traceability analysis more efficient, faster, and with low false positives.

## Integrated R packages for microbiome

**Figure 2.**
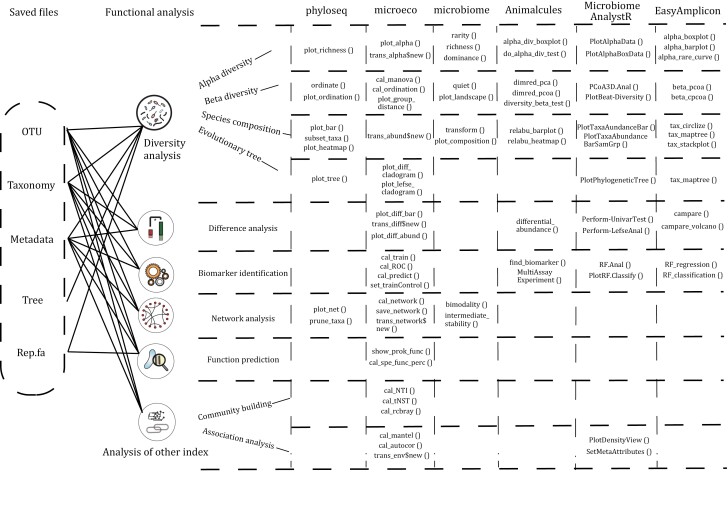
**Introduction to the functions of integrated microbial analysis R packages.** Microbial community analysis can be divided into diversity analysis, difference analysis, biomarker identification, correlation and network analysis, functional prediction, and other microbial community analysis (community building/construction process, association analysis with other indicators).

**Figure 3.**
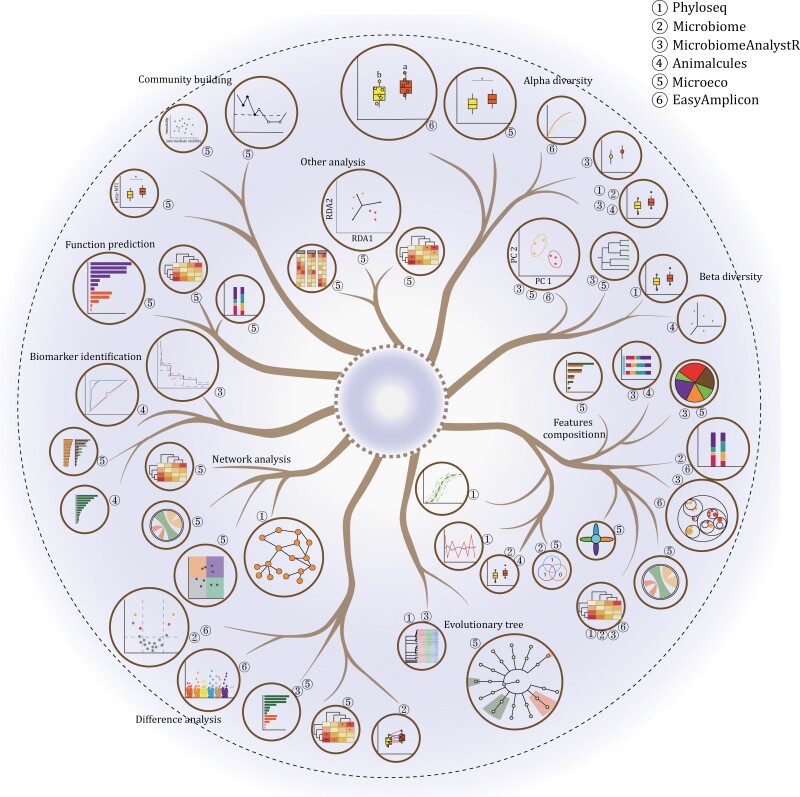
**Typical results of integrated microbial community analysis R packages and comparison of similar results.** Group the analysis results of multiple integrated R packages according to the major categories of microbial community analysis functions. Each main branch in the tree diagram represents a type of microbial community analysis, and there are a total of 10 main branches: feature diversity analysis including (i) alpha diversity analysis, (ii) beta diversity analysis, (iii) features (community taxonomic or functional) composition analysis, (iv) evolutionary or taxonomic tree analysis; (v) difference analysis; (vi) biomarker identification; (vii) correlation and network analysis; (viii) functional prediction; (ix) community building/construction process analysis; (x) other analysis, such as association analysis with other indicators. Each leaf (circle) represents a style of the result displayed in the analysis, and the circle number around the outside of leaf represents the package number of the integrated R package that the analysis result comes from.

As microbiome sequencing becomes more popular, R packages dedicated to microbiome data processing are gradually emerging (Fig. 2). McMurdie and Holmes (2013) developed the phyloseq package: a comprehensive tool for microbiome data (including feature tables, phylogenetic trees, and feature annotation) clustering, integrating data import, storage, analysis, and output. The package utilizes many tools in R for ecological and phylogenetic analyses (vegan, ade4, ape, and picante) and uses ggplot2 to output high-standard figures. The data storage structure uses a S4-like storage system to store all relevant data as a single experiment-level object, thus making it easier to share data and reproduce the analysis. Subsequently, the packages microbiome, the MicrobiomeAnalystR (Chong et al., 2020), microViz (Barnett et al., 2021), and micreobiomeSeq emerged under this framework. Subsequently, the microeco package according to the R6 framework, which provides more analysis functions. With the need for data interactive analysis, Animalcules (Zhao et al., 2021) emerged. EasyMicroPlot also uses an interactive interface for microbiome data exploration, allowing for rapid downstream analysis of the microbiome (Fig. 3; Table 1).

**Table 1.** Comparison of the advantages and limitations of the six integrated R packages.

R package	Function	Advantages	Limitations
Phyloseq	1. Diversity analysis including alpha/beta diversity, community composition, and phylogenetic tree analysis 2. Network analysis	1. Firstly utilize S4 class objects 2. Possess lots of analysis functions based on phyloseq objects 3. The network analysis process is simplified (Fig. S2E) 4. Ordinate analysis can be applied to arrange the order of samples and microbes on heatmap rows and columns (Fig. S2F) 5. Combine evolutionary trees with microbial abundance to display species richness (Fig. S2G) 6. Offer over 30 distance algorithms	1. Introduction to phyloseq objects can be challenging for beginners 2. Statistical tests, including diversity tests and community/feature-level microbial difference analysis, are not well integrated into community analysis 3. Network analysis lacks test, attribute calculation
Microbiome	1. Diversity analysis only including alpha/beta diversity, community composition	1. The alpha diversity index is abundance 2. The t-SNE and CAP ordination algorithms 3. The stacked bar chart for community composition analysis can be sorted by specified microbial features (Fig. S3C) 4. Visualization of individual microbes (Fig. S3D)	1. The t-SNE and CAP ordination analyses frequently encounter errors 2. The statistical tests, including diversity tests, community and feature-level differences tests is not ideal
Microbiome AnalystR	1. Diversity analysis including alpha/beta diversity, community composition, and phylogenetic tree analysis 2. Difference analysis 3. Biomarker identification	1. Various functions ranging from data-cleaning to visualization 2. Multiple algorithms to correct sequencing errors, leading more accurate evaluation of abundance 3. Machine learning can be utilized to extract feature variables (Fig. S4H) 4. Difference analysis using multiple methods, such as LEfSe or metagenomeSeq	1. Difficulties in installing R packages with dependencies 2. Some functions may not work, including network analysis and difference analysis of relative abundance 3. Insufficient explanation of parameters and examples
Animalcules	1. Diversity analysis 2. Difference analysis and biomarker identification	1. SummarizedExperiment package supported 2. Interactively executed in R (Fig. S5A–J) 3. A 3D clustering plot can be generated	1. Unable to save vector graphics and completed tables 2. Insufficient functionality
Microeco	1. Diversity analysis 2. Difference analysis 3. Biomarker identification 4. Network, correlation analysis with other indicators 5. Functional prediction	1. R6 class more expansibility than phyloseq objects 2. Simple function calling 3. Rich plots of diversity and difference analysis (Fig. S6A–H) 4. Unique correlation analysis of other indicators 5. Network analysis functionality (Fig. S6K) 6. FAPROTAX and FUNGuild function prediction	1. New data structures increase the cost of learning time 2. So many functions and dependency caused frequent some malfunctioning
EasyAmplicon	1. Diversity analysis 2. Provide script for preparing STAMP, LEfSe, PICRUSt 1&2, BugBase, FAPROTAX, iTOL 3. Provide slide tutorial for many analyses, such as QIIIME 2	1. It can be used in both command-line mode and interactive mode within RStudio 2. It offers multiple visualization styles, allowing for easy generation of publication-quality figures (Fig. S7) 3. Its open-source code facilitates reproducible analysis and allows for personalized modifications	1. Need using the most popular tools, STAMP, LEfSe, PICRUSt 1&2, BugBase, FAPROTAX, and iTOL 2. Some functions need to be development

### Microbiome data analysis using phyloseq

Phyloseq, using the S4 class object, is more suitable for object-oriented programming and has had a great impact on microbiome data analysis (Figs. 2, 3 and S2A–G, Pipeline 1. phyloseq.Rmd). Through the S4 class object, phyloseq allows the five parts of data (the feature table, feature annotation, metadata, representative sequences, and evolutionary tree) to maintain correspondence under the same framework, and provides a variety of multiple filtering functions on microbial features and samples, allowing the five parts of data to be filtered consistently without considering different among data. It also provides microbiome analysis through microbial data filtering and normalization, diversity calculation (Fig. S2A and S2B), microbial composition visualization (Fig. S2C and S2D), evolutionary tree visualization, and network analysis (Fig. S2E). The beta diversity function provides more than 30 distance algorithms, far more than those provided by packages such as vegan. Secondly, the phyloseq package uses ggplot for graphical visualization (Fig. S2F), which is easier to generate and modify figures. Additionally, phyloseq can integrate the evolutionary tree and feature taxonomic and abundance on tree branches and leaves (Fig. S2G), which makes the tree informative and beautiful.

### Microbiome data analysis using microbiome

The microbiome package also uses S4 class objects, like phyloseq, and can also perform most of the analysis of microbiomes (Figs. 2, 3 and S3A–G, Pipeline 2. Microbiome.Rmd). It includes microbial diversity analysis (Fig. S3A–E), and difference analysis (Fig. S3F and S3G). Compared with phyloseq, the microbiome package is richer in alpha diversity indicators, which provides more than 30 alpha diversity indicators. Secondly, it provides core microbial calculation and visualization functions. In general, it can be used as a complement to phyloseq or in conjunction with it.

### Microbiome data analysis using MicrobiomeAnalystR

MicrobiomeAnalystR is an R package version according to the MicrobiomeAnalyst webserver (Figs. 2, 3 and S4A–J, Pipeline 3. MicrobiomeAnalystR.Rmd). These functions include diversity analysis (Fig. S4A–F), difference analysis (Fig. S4G), biomarker identification (Fig. S4H and S4I), sample sequencing library size overview (Fig. S4J), which are more powerful than the previous two packages. The visualization combines basic packages, ggplot plotting, and interactive plotting. In terms of network analysis, it provides the process of calculating and plotting SparCC networks that are more suitable for microbiome data. However, the package depends on many R packages from CRAN, Bioconductor, and GitHub, so a complete installation of MicrobiomeAnalystR requires a lot of effort.

### Microbiome data analysis using Animalcules

The Animalcules package is an alternative way to analysis in an interactive platform (Figs. 2, 3 and S5A–J, Pipeline 4. Animalcules.Rmd). It is possible to calculate and plot sample statistics in bar plot (Fig. S5A) or interactive pie charts (Fig. S5B), calculate, and visualize alpha diversity dot plot (Fig. S5C), group microbial taxonomic or functional composition heatmap and stack plot (Fig. S5D and S5E), feature abundance in boxplot (Fig. S5F), genus bray distance heatmap (Fig. S5G), ordination analysis (Fig. S5H and S5I), using randomforest, logistic regression to select biomarkers (Fig. S5J), and other analyses. The results of these analyses can often be reanalyzed by interactively modifying parameters, and the images can be interactively zoomed in and out, clicked to see details, and other operations performed by the mouse for better pattern discovery. However, the results cannot be exported as vector format, which do not meet the requirements for publication. Secondly, the analysis content is too little, especially the microbiome network analysis, the correlation analysis between the microbiome and other indicators.

### Microbiome data analysis using microeco

The **microeco** package is very powerful, using R6 class data structure (Figs. 2, 3 and S6A–L, Pipeline 5. microeco.Rmd). It includes microbial diversity (Fig. S6A and S6B) taxonomic composition (Fig. S6C–E), difference (Fig. S6F–H), biomarker (Fig. S6I and S6J), network (Fig. S6K), integrated community structure with environmental factor (Fig. S6L), and phylogenetic diversity analysis. It can complete almost all the current microbiome analysis contents. However, it is not suitable for novices because there is a certain threshold for using R6 class objects. In addition, due to too many functions, the requirements for input data are different, causing some functions are hard to use.

### Microbiome data analysis using amplicon

The package amplicon is an analysis and plotting tool (Figs. 2, 3 and S7A–I, Pipeline 6. Amplicon.Rmd) within the microbiome analysis toolkit EasyMicrobiome (Liu et al., 2023). It enables various diversity analyses, including alpha diversity (Fig. S7A), rarefaction curve (Fig. S7B), clustering distance heatmap (Fig. S7C) and PCoA (Fig. S7D), NMDS, LDA and PCA, taxonomic composition (Fig. S7E and S7F), difference analysis (Fig. S7G and S7H). Then, it can easily generate high-quality figures such as boxplots, scatter plots for diversity analysis, stacked bar plots, circlize plots, and map trees for taxonomic or functional composition. One of its notable features is its ability to finely adjust the presentation of figures, resulting in published-ready figures. Additionally, several tools within the amplicon package are available for microbiome data transformation, facilitating subsequent analysis using tools such as LEfSe and STAMP. However, at the current version, the amplicon package does not provide some functions for network analysis, analysis of microbiome–environment interactions, and analysis of community formation processes. The authors provide some scripts in EasyAmplicon pipeline to do this, mentioned in the published paper plan to finish these functions in the future.

## The best practice for microbiome data analysis in R

The abundance of R packages can hinder microbiome researchers from efficiently selecting appropriate R packages for microbiome-related analyses. Therefore, we organized and selected efficient, commonly used, and user-friendly functions for microbiome data analysis in six categories (Fig. S8): (i) diversity analysis (Figs. S9A–I and S10A–E), (ii) difference analysis (Figs. S10F–I, S11A and S11B), (iii) biomarker identification (Fig. S11C and S11D), (iv) correlation and network analysis (Figs. S11E–I), (v) functional prediction, 6 other microbiome analyses (Fig. S12A–I). All the script can be found in the file Pipeline.BestPractice.Rmd. This led to develop a better microbiome data analysis pipeline.

**Figure 4.**
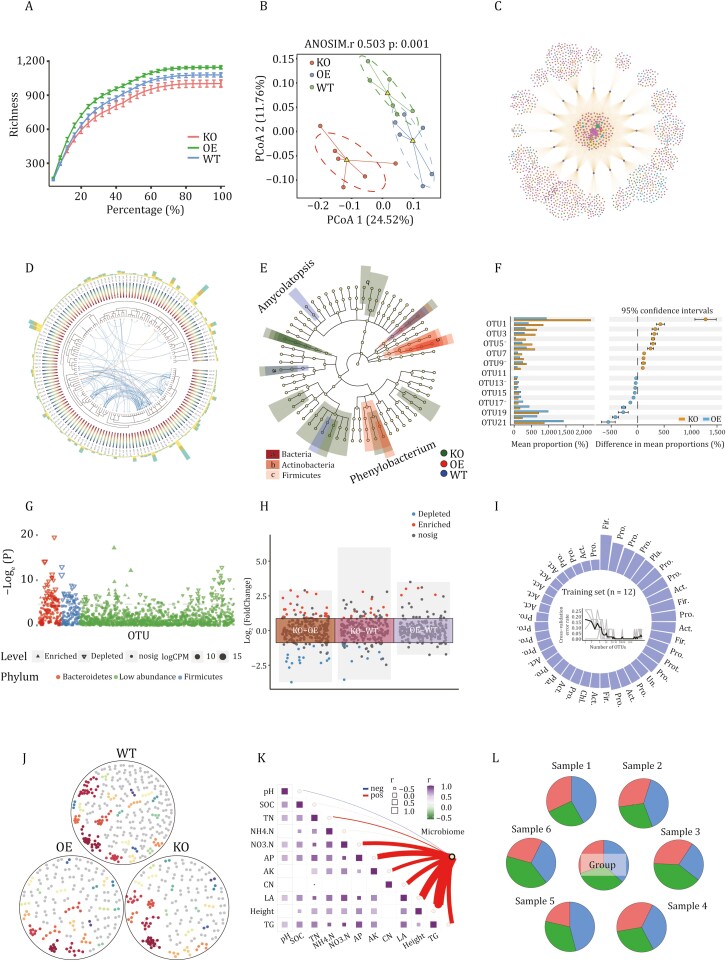
**Examples of the best practice results of microbial community analysis in R language.** The selected results include rarefaction curve (A), principal coordinate analysis scatter plot (B), Venn network graph (C), evolutionary tree (D), LEfSe cladogram (E), difference analysis extended error bar plot in STAMP style (F), difference analysis Manhattan plot (G), difference analysis multi-group volcano plot (H), biomarker selection ring-column chart (I), network graph (J), correlation connection combination graph (K), source tracing analysis pie chart (L).

In this pipeline, we used the amplicon package for alpha diversity rarefaction curve (Figs. 4A and S9A) and PCoA analysis (Figs. 4B and S9B), ggplot2 package for visualization of microbial community composition, ggClusterNet for constructing Venn network (Chen et al., 2021) (Fig. 4C), ggtree and ggtrextre for building evolutionary trees (Fig. 4D), and LEfSe for generating cladograms (Fig. 4E). We employed the stst4, ggplot2, and cowplot packages for difference analysis and generated STAMP plots (Fig. 4F), used edgeR for difference analysis and visualized in Manhattan plots (Fig. 4G), and applied DESeq2 for difference analysis and generated multi-group volcano plots (Fig. 4H). We also used the el071, caret, randomforest, ROC packages for various machine learning analyses and generated microbiome weighted plots (Fig. 4I). Furthermore, we used ggClusterNet for microbiome network analysis (Fig. 4J), constructed network graphs and combined plots to explore the associations between environmental factors and microbiome communities (Fig. 4K). Finally, we used the FEAST package to perform community source tracking analysis and constructed pie charts (Fig. 4L). Other analyses included stacked bar charts of microbial community composition (Figs. S9E and S9H), chord diagrams (Fig. S10A), Venn diagrams (Fig. S10C), Upset diagrams (Fig. S10D), difference analysis volcano plots (Fig. S10F), functional prediction etc.

## Perspective and conclusions

In the past 10 years, the R language and numerous R packages have played an important role in the microbiome data analysis. R language is easy to use and get started. It has attracted many researchers to learn about it. However, there are still some contradictions between supply and demand in the microbiome data analysis. For example, it is often difficult to support multi-threading under the Windows system; second, the speed of many R packages running is relatively slow, although some R packages are written in other languages as supplements; third, the application in microbiome still needs further development. For instance, there is a shortage of packages that allow for the exploration of time-series-based microbial compositions, as well as more robust interactive packages for analyzing complex microbial data. Furthermore, ggplot2 lacks the capability to create complex and combined figures, which fails to meet the visualization requirements for relationships between multiple intricate indicators with microbial community data. Therefore, developing new R packages that are more suitable for drawing complex figures and composite figures would be necessary for microbiome data.

With the development of sequencing technology, data analysis methods have advanced along with the development of R packages contributed to the field of microbiome. These R packages range from classic R packages such as vegan, which has been cited more than 10,000 times, to integrated R packages such as phyloseq, which contain many functions in one package and set a unified data processing framework. These R packages have been able to implement most of the functions of microbiome analysis, from microbial diversity, difference, biomarker identification, correlation and network, phylogenetic analysis, etc. However, these R packages have some redundant functions; for example, phyloseq, microbiome, and others can do microbial diversity analysis. The difference is only in the visualization method and scheme. A similar situation has always existed in microbiome analysis R packages, so we hope that in future developments we will try to de-redundantly use the same part of the content or similar content to highlight the advantages of R packages.

Although these R packages can conduct a lot of functions, they don’t well enough in some specific analyses, for example, alpha and beta diversity analysis, and the outgoing graphs often not add difference detection results to visualize the differences from the figures. In addition, there are still some contents that can continue to be developed, such as applying more machine learning methods to microbiome data and its learning method, model, and important variable evaluation. Secondly, metagenomes are becoming more widely used, and the support of species and functional annotation results based on Kraken (Wood and Salzberg, 2014), MEGAN (Huson et al., 2007), MetaPhlAn2 (Truong et al., 2015), HUMAnN2 (Franzosa et al., 2018), eggNOG-mapper (Huerta-Cepas et al., 2017), etc. is becoming more and more important, and these make the data processed by R rise from megabyte (M) to gigabyte (G). Therefore, faster data processing R packages should be used to the microbiome data analysis process, such as data.table, fst, tidyfst, etc.

The use of appropriate data structures can accelerate the microbiome data processing. At first, we used S4 class objects for microbiome data encapsulation, which can complete a variety of analyses comprehensively and efficiently. The emergence of R6 class objects and other objects has greatly impacted microbiome data processing and largely facilitates it. With the development of the tidy family of R languages, tidy-based data structures have recently emerged for microbiome data mining. For example, the MicrobiotaProcess package (Xu et al., 2023). This structure is more suitable for microbiome data mining, machine learning modeling, and other analyses, which can more easily extract the influence of experimental design, time, space, and other factors on microbiome data in analysis, to discover the deep-seated patterns. We expect the R language to make microbiome analysis more efficient and help everyone discover more about its role in humans, animals, plants, and the environment, and use it for our benefit to make the world a better place.

## Supplementary Material

pwad024_suppl_Supplementary_Material

## Data Availability

No new sequencing data generated by this project.

## Abbreviations

ASV: an amplicon sequence variant
CCA: canonical correspondence analysis;
CSS: cumulative-sum scaling
DCA: decision curve analysis
GO: gene ontology
GSEA: gene set enrichment analysis
GSVA: gene set variation analysis
KEGG: kyoto encyclopedia of genes and genomes
LDA: linear discriminant analysis
LEfSe: linear discriminant analysis effect size
NMDS: non-metric multidimensional scaling
OTU: operational taxonomic unit
PCA: principal components analysis
PCoA: principal coordinate analysis
RLE: relative log expression
ROC: receiver operating characteristic curve
TMM: trimmed mean of M-values
UQ: upper quartile
MED: median of ratios method

## Code availability

All the demo data and scripts are available in GitHub github.com/taowenmicro/EasyMicrobiomeR.
