# Supplementary material for: The best practice for microbiome analysis using R
Source: Protein Cell. 2023 May 2;14(10):713–25. doi: 10.1093/procel/pwad024 (PMC10599642; doi:10.1093/procel/pwad024)
Supplement: pwad024_suppl_Supplementary_Material [file pwad024_suppl_supplementary_material.pdf]

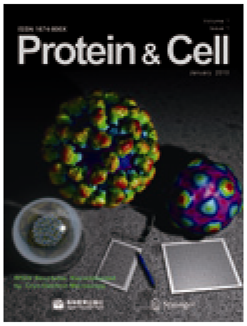

The best practice for microbiome analysis using R

Journal:	*Protein & Cell*
Manuscript ID	P-C-02-2023-0084.R1
Manuscript Type:	Review
Date Submitted by the Author:	28-Mar-2023
Complete List of Authors:	Wen, Tao; Chinese Academy of Agricultural Sciences; Nanjing Agricultural University<br>Niu, Guoqing; Nanjing Agricultural University<br>Chen, Tong; China Academy of Chinese Medical Sciences<br>Shen, Qirong; Nanjing Agricultural University<br>Yuan, Jun; Nanjing Agricultural University,<br>Liu, Yong-Xin; Chinese Academy of Agricultural Sciences
Keywords:	microbiome, R package, data analysis, visualization

SCHOLARONE™
Manuscripts

The best practice for microbiome analysis using R

Tao Wen[1,2,‡], Guoqing Niu[2,‡], Tong Chen[3], Qirong Shen[2], Jun Yuan[2,*], Yong-Xin Liu[1,*]

[1]Shenzhen Branch, Guangdong Laboratory of Lingnan Modern Agriculture, Genome Analysis Laboratory of the Ministry of Agriculture and Rural Affairs, Agricultural Genomics Institute at Shenzhen, Chinese Academy of Agricultural Sciences, Shenzhen, Guangdong 518120, China

[2]The Key Laboratory of Plant Immunity Jiangsu Provincial Key Lab for Organic Solid Waste Utilization Jiangsu Collaborative Innovation Center for Solid Organic Waste Resource Utilization, National Engineering Research Center for Organic-based Fertilizers, Nanjing Agricultural University, Nanjing 210095, China

[3]National Resource Center for Chinese Materia Medica, China Academy of Chinese Medical Sciences, Beijing, China

[‡]These authors contributed equally to this work

[*]Correspondence: junyuan@njau.edu.cn (J. Yuan), liuyongxin@caas.cn (Y.-X. Liu)

For Peer Review

Abstract

With the gradual maturity of sequencing technology, many microbiome studies have published, driving the emergence and advance of related analysis tools. R language is the widely used platform for microbiome data analysis for powerful functions. However, tens of thousands of R packages and numerous similar analysis tools have brought major challenges for many researchers to explore microbiome data. How to choose suitable, efficient, convenient, and easy-to-learn tools from the numerous R packages has become a problem for many microbiome researchers. We have organized 322 common R packages for microbiome analysis and classified them according to application categories (diversity, difference, biomarker, correlation and network analysis, functional prediction, and others), which could help researchers quickly find relevant R packages for microbiome analysis. Furthermore, we systematically sorted the integrated R packages (**phyloseq**, **microbiome**, **MicrobiomeAnalystR**, **Animalcules**, **microeco**, and **amplicon**) for microbiome analysis, and summarized the advantages and limitations, which will help researchers choose the appropriate tools. Finally, we thoroughly reviewed the R packages for microbiome analysis, summarized most of the common analysis content in the microbiome, and formed the most suitable pipeline for microbiome analysis. This paper is accompanied by hundreds of examples with 10,000 lines codes, which can help beginners to learn (C1-2 in GitHub), also help analysts compare and test different tools (C3-4 in GitHub). This paper systematically sorts the application of R in microbiome, providing an important theoretical basis and practical reference for the development of

For Peer Review

better microbiome tools in the future. All the code is available at GitHub: https://github.com/taowenmicro/EasyMicrobiomeR.

Keywords R package, microbiome, data analysis, visualization

Introduction

The metagenomic analysis is used to study microbial diversity, structure, and function by sequencing, quantifying, annotating, and analyzing DNA and/or RNA sequences of microbial communities or microbiota. The commonly used high-throughput sequencing technology in microbiome research is mainly known as amplicon sequencing and shotgun metagenomic sequencing. Amplicon sequencing with the advantages of low cost, mature analysis system, and simple analysis process was widely used in microbiome research. Shotgun metagenomic sequencing provided the functional information of microbes and more accurate information on the microbial composition with the higher sequencing cost and large amount of computational resources needed. The detail pipeline for both sequencing have been systemically summarized in our previous review (Liu et al., 2021b) As an important component of biodiversity, microbial communities play a vital role in biology, ecology, biotechnology, agriculture, and medicine. Various bioinformatics methods are required for microbial community analysis, which mainly includes three parts: 1) data preprocessing, 2) quantification and annotation, and 3) statistics and visualization (Fig. 1A). In the preprocessing step, the raw data is filtered and quality controlled to ensure data quality. In the quantification and annotation step, tools and databases are used to identify microbial representative sequences and annotate microbial taxonomy and

For Peer Review

function. The first two parts of microbial community analysis have been well discussed and could be well done according to our previous papers (Liu et al., 2023). Finally, in the statistics and visualization step, various statistical methods are used to explore microbial community diversity, structure, and potential functions.

With the development of high-throughput sequencing technology, plenty of studies were performed with amplicon-sequencing technology (Thompson et al., 2017; Proctor et al., 2019) and shotgun metagenomes sequencing (Carrión et al., 2019; Paoli et al., 2022), which led to the development of microbiome analysis methodologies, software, and pipelines, e.g., QIIME (Caporaso et al., 2010), Mothur (Schloss et al., 2009), USEARCH (Edgar, 2010), VSEARCH (Rognes et al., 2016), QIIME 2 (Bolyen et al., 2019), Parallel-Meta Suite (Chen et al., 2022), EasyAmplicon (Liu et al., 2023), Kraken (Wood and Salzberg, 2014), MEGAN (Huson et al., 2007), MetaPhlAn2 (Truong et al., 2015), HUMAnN2 (Franzosa et al., 2018) etc. As the most crucial and basic procedure for amplicon sequencing data analysis, OTU (Operational taxonomic unit) clustering method was popular before the year of 2015 while non-clustering methods were gradually developed and widely used recently. Currently, the common non-clustering methods include DADA2 (Callahan et al., 2016), deblur (Amir et al., 2017), unoise3 (Edgar, 2016). One of the most representative non-clustering algorithms among them is DADA2, which was created with R language. It makes the R language (Ihaka and Gentleman, 1996) occupy an important position in raw data processing for amplicon sequencing. Compared with many software that can be used in upstream steps of microbiota sequencing data analysis, the downstream analysis steps rely on the R

For Peer Review

language heavily with various packages. These analyses mainly include: 1) Diversity analysis; 2) Difference analysis; 3) Correlation and network analysis; 4) Biomarker identification; 5) Functional predictions; 6) Integrative analysis of microbial communities with other indicators (including phylogenetic analysis, multi-omics integration, and environmental factor analysis, *etc.*). In addition to the kinds of multivariate statistical analysis that can be done in R, there are diversified data-cleaning packages that allow data to be transformed among different analyses.

R is a free, open-source language and environment for data statistical analysis and visualization, which was created by Ross Ihaka and Robert Gentleman from the University of Auckland in New Zealand and now is responsible by the "R Development Core Team". Compared with other analysis tools, such as SPSS, MINITAB, MATLAB, which are more suitable for the statistics of processed and standardized data, R language can handle processed data as well as raw data. R can easily implement almost all analysis methods, many of the latest methods or algorithms were first exhibited in it. Furthermore, R shows excellent data visualization, particularly for complex data. The powerful and flexible interactive analysis is also an advantage of R, meanwhile enabling visual data exploration. The functionality of the R language relies heavily on thousands of R packages, which provide a wide variety of data processing and analysis strategies, allowing almost any data analysis process to be done in R. The total number of R packages published on CRAN is 18,981, and Bioconductor is 2,183 (by January 31, 2023). These packages demonstrated the powerful data process and analysis performance of R.

For Peer Review

106 In recent years, numerous R packages have been developed on the R platform for the downstream analysis of microbiome, which have made important contributions to the associated-research field. However, the increasing number of downstream analysis R packages has reached a dizzying level (Fig. 1B). In addition, integrated R packages containing a large amount of microbiome analysis content, such as **phyloseq** (McMurdie and Holmes, 2013), **microeco** (Liu et al., 2021a), and **amplicon** (Liu et al., 2023), have gradually emerged. This abundance of R packages provides microbiome analysts with more choices, but also makes it difficult to identify the most suitable tools among many similar analysis tools. Furthermore, this plethora of R packages make it difficult for beginners to embark on a well-organized learning path for microbiome analysis. Therefore, it is urgent to compare similar analysis functions, and extract the similarities and differences functions, to select the best process for microbiome analysis and help beginners learn more effectively.

119 This paper attempts to sort and run the 322 common R packages (Fig. S1), especially the integrated R packages for microbiome analysis, and complete the following three parts: 1) compare different R package analysis processes according to the functional categories of microbiome analysis, analyze the results, and summarize example code; 2) organize the content of six integrated R packages according to the functional categories of microbiome analysis, compare the analysis results, and generate example code; 3) based on all R packages, select the optimal analysis approach using R language and provide example code for reference and learning to researchers.

127 Preparing microbiome data analysis

For Peer Review

Downstream analysis of microbiome requires the preparation of five data files, including a feature table, a feature annotation file, a sample metadata file, a phylogenetic tree, and representative sequences. For beginners, it is important to understand the format and basic data structure of these files and learn how to import these files into R language. Furthermore, different analytical contents often have different requirements for data, and it is necessary to learn some data manipulation skills to meet the demands of various functions. Finally, it is necessary to learn the basics of R plotting to facilitate the presentation of results.

Data preparation and cleaning

After the process of sequence data preprocessing and quantification and annotation, we need to further analysis the output files, including importing these files, cleaning data, and converting format and content which required for subsequent microbiome analysis in R. Before statistical analysis, we must master the basic procedure of R language to cope with the data input requirements of different packages. This section includes: importing, organizing, filtering, basic calculations, conversion, normalization, and modification of data. Five data forms are frequently used from raw data processing, including feature tables (file formats are .csv/.txt/.xlsx/.biom, typically used taxonomic and functional tables, including OTU/ASV/taxonomy/gene/module/pathway tables), feature annotation (.csv/.txt/.xlsx/.biom), sample metadata (.csv/.txt), evolutionary/phylogenetic trees (.nwk/.tree), representative sequences (.fasta/.fas/.fa). All the data cleaning-related packages show in Fig. 1C. Tabular data input for microbial community is primarily accomplished using functions such as read.table(), read.delim(),

For Peer Review

and read.csv() in the **utils** package (Code 1A, script in GitHub). The reading of evolutionary tree files depends on functions like read.tree() in the **ape**/**ggtree**/**treeio** package, or read_tree() in the **phyloseq** package. For reading representative sequence files in microbiome, the readDNAStringSet() in the **Biostrings** package (Pages et al., 2016) is typically used. Currently, big data integration of microbiome has become a trend, and leading to the emergence of R packages for integrated data from multiple studies, likes **curatedMetagenomicData** (Pasolli et al., 2017). The package only needs to import the package and could re-analysis the curated data, rather than input in raw sequencing data.

The basic idea of data organization can be summarized as three steps: splitting the data, processing with functions, and combining the output results into the desired format. The functions of basic packages in R can be combined to meet most requirements of the microbiome data operations. For example, the "for loop" combined with the basic statistical functions [sum(), mean(), sd(), etc.] can be used to perform basic statistical analysis and data transformations for microbial relative abundance (Code 1B); the **base** package provides the apply family of functions, including apply(), sapply(), lapply(), tapply(), aggregate(), etc., which can be applied to quickly complete the three stages of data processing. The apply family of functions provides a framework that acts as an alternative to "for loop" and is much faster than the basic "for loop" function in R (Code 1B). A similar **purr** (https://github.com/tidyverse/purrr) package can be used in place of "for loop" to perform efficient operations.

The **plyr** (Wickham and Wickham, 2020) package was upgraded from package of

For Peer Review

base with a variety of data sorting processes for kinds of data frames, lists, etc. The **plyr** package provides three data processing stages "Split - Apply - Combine" in one function, and the **plyr** package implements grouping transformations between R types (vector, list, and data frame) and basically replaces the apply family of functions in the **base** package. It can easily handle grouping calculations, e.g., microbial abundance at different taxonomy levels (Code 1C). The **reshape2** (Wickham, 2012) package provides the long-wide format transformation during data processing, and since **ggplot2** (Wickham, 2011) plotting functions and most modeling functions, such as lm(), glm(), gam(), often use long data, microbiome data are general showed as wide form, so the transformation of microbiome data for plotting can be done using **reshape2** (Code 1D), which provides the long-wide format transformation during data processing.

The **dplyr** (Wickham et al., 2014) package is a member of the tidyverse family, innovatively abandoning the common form of data preservation in R rather than using the tibble format (more powerful than data.frame format) for data processing, which can more efficiently complete the data frame selection, merging and statistics within row and column, and data frame length and width format changes, the "%>%" pipeline symbol can be used to complete more complex data processing. The tibble format can store data during the analysis and modeling process, which is important for data analysis. For example, we demonstrated the use of **dplyr** and pipeline to run random forest modeling and the selection process of important variables (Code 1E).

Visualization in R language

In most cases, we are used to plotting standard graphs in microbiome data display

For Peer Review

such as alpha/beta diversity, taxonomic composition. All the visualization-related packages show in Fig. 1C. Due to the widespread use of **ggplot2** (Code 2A), many extension packages have emerged to extend based on **ggplot2** with a high capacity of plotting styles, colors, and themes. These packages mainly include **ggtern** plotting ternary graphs in Code 2B (Hamilton and Ferry, 2018), **ggraph** plotting network graphs in Code 2C (Si et al., 2020), **ggtree** plotting evolutionary tree or cladogram in Code 2D (Xu et al., 2022) , the **ggalluvial** package, the **ggVennDiagram** package (Code 2E), the **ggstatsplot** package plotting pie chart, and the **ggpubr** package providing many various themes and colors of output. In addition, the **pheatmap** (Kolde, 2012) and **ComplexHeatmap** package (Gu, 2022) based on the grid mapping system plots the relative abundance of features in different samples (Code 2F), the **VennDiagram** package (Chen and Boutros, 2011) could show the number of features in different samples. The **Upset** package (Conway et al., 2017), which draws Upset view is a new form plotting similar to Venn diagram. The base-based drawing system is complex and difficult to learn, while it is a good choice for complex graph drawing, such as the **circlize** (Gu et al., 2014) package (Code 2G), which draws chord diagrams composed of microbiota.

Additionally, there is often a lot of microbiome mapping work that involves a combination of graphics. At present, many tools in R can combine graphics, such as **cowplot**, **patchwork**, and **aplot**. The **patchwork** package has the most powerful functions and supports modular splicing graphics (Code 2H).

Microbial community analysis

For Peer Review

We have categorized the analysis of microbiome data into the following six major types in Fig. 1D: diversity analysis, difference analysis, biomarkers identification, correlation and network analysis, functional prediction, and other microbiome analyses (including source tracking analysis, community assembly processes, and analysis of associations between microbiota and environmental factors). Then, we would have organized, compared, and summarized all relevant R packages.

Diversity analysis

Microbial community diversity mainly includes alpha diversity (Richness, Shannon, Simpson, Chao1, ACE, etc.), rarefaction curve, beta diversity (ordination and clustering analysis), taxonomic or functional composition. Here must introduce the package **vegan** (Oksanen et al., 2007), an abbreviation for Vegetation Analysis, written by nine quantitative ecologists, including Oksanen from Finland, which is initially used for specifical dealing with data on community ecology. The package provides a variety of methods for data standardization and transformation. For example, data used for alpha diversity analysis can be normalized at the same sequencing depth with *rrarefy()*, and data for ordination analysis can be normalized with the *decostant()* (Code 3A). After the sequencing data are sampling normalization, diversity calculation can be more reasonable. In addition, alpha diversity metrics calculation can also be carried out with the **ade4** (Dray and Dufour, 2007), **adespatial** (Dray et al., 2019), and **picante** packages (Kembel et al., 2010). For example, phylogenetic diversity can be calculated using the pd() in the **picante** package (Code 3A). **Vegan** not only allows for alpha diversity analysis, but also provides functions such as rda() for conducting principal components

For Peer Review

analysis (PCA) and redundancy analysis (RDA), cca() for conducting correspondence analysis (CA) and canonical correspondence analysis (CCA), decorana() for conducting decision curve analysis (DCA), and metaMDS() for conducting non-metric multidimensional scaling (NMDS) for microbiome ordination analysis (Code 3B). The prcom() in **stats** package can be used for principal component analysis (PCA), which is a kind of dimension reduction analysis. The mca() provided by the **MASS** package and the MCA() provided by the **FactoMineR** package can be used for multiple correspondence analysis (Code 3B); the **ape** package provides the pcoa() function for principal coordinate analysis (PCoA); the **MASS** package provides lda() for linear discriminant analysis (LDA, Code 3C). Before running many ordination operations, it is often necessary for community clustering. The vegdist() in the **vegan** package can calculate euclidean, manhattan, bray, canberra, and other distances (Code 3B). In addition, distance calculation can also be done using dist() of **stats** package. The distance matrix can be used for clustering analysis in addition to ordination analysis. The hclust() in the **stats** package can be used for clustering analysis, a similar function can be achieved with the **facteoextra**, **kmeans** packages (Code 3D). Microbial composition analysis mainly used to display the abundance of microbes, and the **dplyr** package is needed to organize the data then display with **ggplot2** subsequently.

Difference analysis

Difference analysis is divided into community-level analysis and feature-level (any hierarchy of taxonomy and function) analysis. Community-level difference analysis is mainly performed with functions including *adonis()*, *anosim()*, and *mrpp()*

For Peer Review

in **vegan** package, and *mantel.test()* in **ape** package (Code 4A). The R package for compositional data difference analysis in the feature level can utilize the *wilcox.test()* (Code 4B) and *t.test()* (Code 4C) in the **stats** package. Subsequently, data correction algorithms were developed specifically for sequencing data, such as the upper quartile (UQ), trimmed mean of M-values (TMM) (Code 4C), and relative log expression (RLE) harbored in the **edgeR** package (Chen et al., 2014) (Code 4D). Median of ratios method (MED) in **DESeq2** package (Love et al., 2014) (Code 4E), and cumulative-sum scaling (CSS) algorithm in **metagenomeSeq** (https://github.com/sirusb/metagenomeSeq) package (Code 4F). Furthermore, the **ALDEx2** package provides polynomial models which can be used to infer feature abundance and calculate feature differences with non-parametric tests, t-tests, or generalized linear models (Code 4G). The **ANCOM-BC** package attempts to address sample heterogeneity by correcting bias with a log-linear model. In addition, other R packages for microbiome data correction and difference tests include **limma** (Smyth, 2005) (Code 4H), **DR**, **ANCOM** (Lin and Peddada, 2020) (Code 4I), **corncob** (Code 4J), **Maaslin2** (Code 4K), etc. Nearing et al. (2022) showed that they compared these difference analysis methods and proposed that **ALDEx2** and **ANCOM-II** (anchom_v2.1.R, Code 4L) were the best performers in the difference analysis of microbial communities. As for the significance test, different packages use different methods for significance testing. For example, Fisher test was used in **edgeR** package; Wald test was used in **DESeq2 and corncob** package; t-test was used in **limma** package. There was other method for significance test, likes Wilcoxon rank-sum test (**ALDEx2** and **ANCOM-II**), ANOVA (**Maaslin2**) etc.

For Peer Review

Biomarker identification

Characteristic microbial consortia were explored to explain certain questions, such as the biomarkers of the gut in obese or hypertensive populations, or of soil in Fusarium wilt develops, etc. Microbes selected through difference analysis are often unable to determine whether they represent the main differences of concern. Therefore, weight analysis or machine learning methods are used to further distinguish the feature microbes.

The main ones commonly used for weighted analysis are linear discriminant analysis effect size (LEfSe), PCA, etc (Code 5A). LEfSe is developed specifically for microbiome data, and the core functionality is implemented using the packages **LDA** (Fisher, 1936) and **MASS** (Ripley et al., 2013). By extracting the loading matrix of PCA ordination, the microbiome with the greatest impact on the sample variation are found as biomarkers. (Code 5B)

In terms of machine learning, the random forest model, which is widely used in microbiome analysis, is implemented by using the **randomforest** package (Liaw and Wiener, 2002) (Code 5C). There are many other decision tree-based machine learning models, such as the **mboost** (Hofner et al., 2014) package provides boosting-based algorithms, the **e1071** (Dimitriadou et al., 2008) package provides support vector machines *svm()* in Code 5D, and plain Bayes *naiveBayes()*. The **xgboost** package can integrate many tree models together to form a strong classifier, which can prevent overfitting via many strategies, including regularization terms, shrinkage, and column subsampling, etc. In addition, the **pROC** (Robin et al., 2011) package is used to plot the operating characteristic curve (ROC, Code 5D) to evaluate the efficiency of

For Peer Review

machine learning models. The **Caret** package provides cross-validation to determine the number of features (Kuhn, 2009). Currently, Jakob et al (2021) developed an open-source R package **SIAMCAT,** a powerful yet user-friendly computational machine learning toolkit tailored to the characteristics of microbiome data.

Correlation and network analysis

Microbial co-occurrence network analysis is used to find microbial modules that may have mutualistic relationships. Co-occurrence network analysis mainly includes the calculation of correlations, network visualization, and the calculation of network properties. The common R packages for calculation of correlations are **psych** (Revelle and Revelle, 2015) (Code 6A), **WGCNA** (Langfelder and Horvath, 2008) (Code 6B), **Hmisc** (Harrell Jr and Harrell Jr, 2019) (Code 6C), and **SpiecEasi** (Kurtz et al., 2015) (Code 6D). Among these R packages, **WGCNA** has the highest calculation speed, while requiring additional p-value correction; **psych** can calculate correlation with correct p-value, but the speed is very low; the **SpiecEasi** package can use the sparcc method to perform a more suitable method for microbiome data to calculate the correlation matrix, and can call multiple-threads to accelerate the calculation. R packages for network visualization and attribute calculation can use **igraph** (Code 6E), **network**, and **ggraph** packages (Code 6F). These R packages contain many layout algorithms for network visualization. In addition, **network** packages combined with **ggplot2** to visualize the network are easier to modify. **Sna** and **ggraph** packages have many visualization layout algorithms to increase the styles of network visualization. With the increasing use of network analysis in the microbiome analysis, more attention

For Peer Review

is paid to network modularity and the key groups through network modules. The **WGCNA** package provides a complete framework to quickly complete the correlation calculation, network module calculation, module feature vector calculation, and other network properties exploration. The recent development of the **ggClusterNet** (Wen et al., 2022) package (Code 6G) provides a unified framework for microbiome networks and designs a variety of unique module-based visualization algorithms to visualize the module relationships in the network.

Functional prediction

The **Tax4Fun** (Aßhauer et al., 2015) R package (Code 7A) for functional prediction of 16S rDNA has been developed to more accurately predict changes in microbial community function using amplicon data. The package has been updated to **Tax4Fun2** (Wemheuer et al., 2020). **Microeco** can implement FAPROTAX (Louca et al., 2016) prediction for bacteria/archaea and FUNGuild (Nguyen et al., 2016) prediction for fungi, which is based on the database of taxonomic functional description from curated published papers. Functional prediction enables the prediction of microbial community function and subsequent statistical analysis. Additionally, **vegan** can be used for diversity analysis, while **edgeR**, **DEseq2**, and **limma** packages can be used for difference analysis. For functional enrichment, the **clusterProfiler** (Code 7B) package can perform GO, KEGG, GSEA and GSVA enrichment, which considers gene/pathway abundance and is recommended. Furthermore, the **clusterProfiler** package provides plot functions based on the **ggplot** syntax, allowing to plot appealing graphics in a simple manner. Gene/pathway network analysis can be performed using **WGCNA** for calculation, and **ggClusterNet** for network parameter calculation and

For Peer Review

visualization. However, the reliability of functional prediction results, particularly for environmental samples, is currently disputed (Wemheuer et al., 2020), and therefore, further verification of analysis results is often required.

Other microbiome analysis

Analysis for microbial community formation process commonly used in the framework proposed by Stegen et al. (2013) to calculate βNTI and RC-Bray indices with R packages **minpack.lm**, **picante**, **Hmisc**, **eulerr**, **FSA**, **ape**, **stats4**, and others (Code 8A). Ning et al. (2020) used a phylogenetic binning-based null model analysis to infer quantitative mechanisms underlying community assembly, and developed the R package **iCAMP** (Code 8B). It allows for the quantitative assessment of the relative importance of different ecological processes (e.g., homogenizing selection, heterogenizing selection, dispersal, and drift) on both the entire community and each phylogenetic bin (which is usually composed of taxa from a single family or order with distinct ecological characteristics). In addition, the package also provides neutral theory models, phylogenetic and taxonomic null model analyses at both the community and clade levels, calculation of niche differences and phylogenetic distances between clades, and tests for phylogenetic signals within individual phylogenetic bins.

Microbial communities were often used to analyze the correlation with environment indicators, for example, *mantel.test()* provided by the **vegan** package was used to examine the correlation between microbial communities and environment indicators, and using *wascores()*, *mantel.correlog()* to detect the phylogenetic signal between microbial communities and environmental factors (Code 8C). In addition, the **ggClusterNet** package can be used to calculate the co-occurrence relationships

For Peer Review

between microbes/microbiome and environmental factors, and generated publish-ready figures (Code 8D).

Knights et al. (2011) proposed the microbiome traceability tool source tracker in R language. Metcalf et al. (2016) predicted the time of death and tracked the source microbes of real cadavers on microbial communities, then microbial traceability analysis gradually popular. Shenhav et al. (2019) proposed a new algorithm in R, **FEAST** (Code 8E), which makes microbial traceability analysis more efficient, faster, and with low false positives.

Integrated R packages for microbiome

As microbiome sequencing becomes more popular, R packages dedicated to microbiome data processing are gradually emerging (Fig. 2). McMurdie and Holmes (2013) developed the **phyloseq** package: a comprehensive tool for microbiome data (including feature tables, phylogenetic trees, and feature annotation) clustering, integrating data import, storage, analysis, and output. The package utilizes many tools in R for ecological and phylogenetic analyses (**vegan**, **ade4**, **ape**, and **picante**) and uses **ggplot2** to output high-standard figures. The data storage structure uses a S4-like storage system to store all relevant data as a single experiment-level object, thus making it easier to share data and reproduce the analysis. Subsequently, the packages **microbiome** (https://github.com/microbiome/microbiome), the **MicrobiomeAnalystR**(Chong et al., 2020), **microViz** (Barnett et al., 2021), and **micreobiomeSeq** emerged under this framework. Subsequently, the **microeco** package according to the S6 framework, which provides more analysis functions. With the need

For Peer Review

for data interactive analysis, **Animalcules** (Zhao et al., 2021) emerged. **EasyMicroPlot** (https://github.com/xielab2017/EasyMicroPlot) also uses an interactive interface for microbiome data exploration, allowing for rapid downstream analysis of the microbiome (Fig. 3; Table1).

Microbiome data analysis using phyloseq

Phyloseq, using the S4 class object, is more suitable for object-oriented programming and has had a great impact on microbiome data analysis (Figs. 2/3, Fig. S2A-I, Pipeline 1. phyloseq.Rmd). Through the S4 class object, **phyloseq** allows the five parts of data (the feature table, feature annotation, metadata, representative sequences, and evolutionary tree) to maintain correspondence under the same framework, and provides a variety of multiple filtering functions on microbial features and samples, allowing the five parts of data to be filtered consistently without considering different among data. It also provides microbiome analysis through microbial data filtering and normalization, diversity calculation (Fig. S2A-B), microbial composition visualization (Fig. S2C-D), evolutionary tree visualization, and network analysis (Fig. S2E). The beta diversity function provides more than 30 distance algorithms, far more than those provided by packages such as **vegan**. Secondly, the **phyloseq** package uses **ggplot** for graphical visualization (Fig. S2F), which is easier to generate and modify figures. Additionally, **phyloseq** can integrate the evolutionary tree and feature taxonomic and abundance on tree branches and leaves (Fig. S2G), which makes the tree informative and beautiful.

Microbiome data analysis using microbiome

For Peer Review

The **microbiome** package also uses S4 class objects, like **phyloseq**, and can also perform most of the analysis of microbiomes (Figs. 2/3, Fig. S3, Pipeline 2. Microbiome.Rmd). Compared with **phyloseq**, the **microbiome** package is richer in alpha diversity indicators, which provides more than 30 alpha diversity indicators. Secondly, it provides core microbial calculation and visualization functions. In general, it can be used as a complement to **phyloseq** or in conjunction with it.

Microbiome data analysis using MicrobiomeAnalystR

MicrobiomeAnalystR is an R package version according to the MicrobiomeAnalyst webserver (Figs. 2/3, Fig. S4A-I, Pipeline 3. MicrobiomeAnalystR.Rmd). These functions include diversity (Fig. S4A-E), difference (Fig. S4F), the evolutionary tree, LEfSe, machine learning (Fig. S4G-H), network analysis, etc., which are more powerful than the previous two packages. The visualization combines basic packages, **ggplot** plotting, and interactive plotting. In terms of network analysis, it provides the process of calculating and plotting SparCC networks that are more suitable for microbiome data. However, the package depends on many R packages from CRAN, Bioconductor, and GitHub, so a complete installation of **MicrobiomeAnalystR** requires a lot of effort.

Microbiome data analysis using Animalcules

The **Animalcules** package is an alternative way to analysis in an interactive platform (Figs. 2/3, Fig.S5A-I, Pipeline 4. Animalcules.Rmd). It is possible to calculate and plot sequence statistics (Fig. S5A) and output interactive pie charts (Fig. S5B), calculate, and visualize alpha diversity boxplot, group microbial taxonomic or

For Peer Review

functional composition stacked histogram plotting (Fig. S5C-G), ordination analysis (Fig. S5H), cluster analysis and heatmap, difference analysis by **DESep2**, **limma**, using randomforest, logistic regression to select biomarkers, and other analyses (Fig. S5J). The results of these analyses can often be reanalyzed by interactively modifying parameters, and the images can be interactively zoomed in and out, clicked to see details, and other operations performed by the mouse for better pattern discovery. However, the results cannot be exported as vector format, which do not meet the requirements for publication. Secondly, the analysis content is too little, especially the microbiome network analysis, the correlation analysis between the microbiome and other indicators.

Microbiome data analysis using microeco

The **microeco** package is very powerful, using R6 class data structure (Figs. 2/3, Fig.S6A-I, Pipeline 5. microeco.Rmd). It includes microbial diversity (Fig. S6A-G), difference (Fig. S6H-I), network (Fig. S6J), biomarker (Fig. S6K), integrated microbial and environmental factor (Fig. S6L), and phylogenetic diversity analysis. It can complete almost all the current microbiome analysis contents. However, it is not suitable for novices because there is a certain threshold for using S6 class objects. In addition, due to too many functions, the requirements for input data are different, causing some functions are hard to use.

Microbiome data analysis using amplicon

The package **amplicon** is an analysis and plotting tool within the microbiome analysis toolkit EasyMicrobiome (Liu et al., 2023). It enables various diversity analyses, including alpha diversity, rarefaction curve, PCoA, NMDS, LDA and PCA, taxonomic

For Peer Review

composition. Then, it can easily generate high-quality figures such as boxplots, scatter plots for diversity analysis, stacked bar plots, circlize plots, and map trees for taxonomic or functional composition (Figs. 2/3, Fig.S7A-I, Pipeline 6. Amplicon.Rmd). One of its notable features is its ability to finely adjust the presentation of figures, resulting in published-ready figures. Additionally, several tools within the amplicon package are available for microbiome data transformation, facilitating subsequent analysis using tools such as LEfSe and STAMP. However, at the current version, the amplicon package does not provide some functions for network analysis, analysis of microbiome-environment interactions, and analysis of community formation processes. The authors provide some scripts in EasyAmplicon pipeline to do this, mentioned in the published paper plan to finish these functions in the future.

The best practice for microbiome data analysis in R

The abundance of R packages can hinder microbiome researchers from efficiently selecting appropriate R packages for microbiome-related analyses. Therefore, we organized and selected efficient, commonly used, and user-friendly functions for microbiome data analysis in six categories (Fig. S8): 1) diversity analysis (Figs. S9A-I; Figs.S10A-E), 2) difference analysis (Figs. S10F-I; Figs. S11A-B), 3) biomarker identification (Figs. S11C-D), 4) correlation and network analysis (Figs. S11E-I), 5) functional prediction, 6 other microbiome analyses (Figs.S12A-I). All the script can be found in the file Pipeline.BestPractice.Rmd. This led to develop a better microbiome data analysis pipeline.

In this pipeline, we used the **amplicon** package for alpha diversity rarefaction

For Peer Review

483 curve (Fig. 4A; Fig. S9A) and PCoA analysis (Fig. 4B; Fig. S9B), **ggplot2** package for
484 visualization of microbial community composition, **ggClusterNet** for constructing
485 Venn network (Fig. 4C), **ggtree** and **ggtrextre** for building evolutionary trees (Fig. 4D),
486 and LEfSe for generating cladograms (Fig. 4E). We employed the **stst4**, **ggplot2**, and
487 **cowplot** packages for difference analysis and generated STAMP plots (Fig. 4F), used
488 **edgeR** for difference analysis and visualized in Manhattan plots (Fig. 4G), and applied
489 **DESep2** for difference analysis and generated multi-group volcano plots (Fig. 4H). We
490 also used the **el071**, **caret**, **randomforest**, **ROC** packages for various machine learning
491 analyses and generated microbiome weighted plots (Fig. 4I). Furthermore, we used
492 **ggClusterNet** for microbiome network analysis (Fig. 4J), constructed network graphs
493 and combined plots to explore the associations between environmental factors and
494 microbiome communities (Fig. 4K). Finally, we used the **FEAST** package to perform
495 community source tracking analysis and constructed pie charts (Fig. 4L). Other
496 analyses included stacked bar charts of microbial community composition (Figs.
497 S9E/H), chord diagrams (Fig. S10A), Venn diagrams (Fig. S10C), Upset diagrams (Fig.
498 S10D), difference analysis volcano plots (Fig. S10E), functional prediction etc.

499 Perspective and conclusions

500 In the past ten years, the R language and numerous R packages have played an
501 important role in the microbiome data analysis. R language is easy to use and get started.
502 It has attracted many researchers to learn about it. However, there are still some
503 contradictions between supply and demand in the microbiome data analysis. For
504 example, it is often difficult to support multi-threading under the Windows system;

For Peer Review

secondly, the speed of many R packages running is relatively slow, although some R packages are written in other languages as supplements; third, the application in microbiome still needs further development. For instance, there is a shortage of packages that allow for the exploration of time-series-based microbial compositions, as well as more robust interactive packages for analyzing complex microbial data. Furthermore, **ggplot2** lacks the capability to create complex and combined figures, which fails to meet the visualization requirements for relationships between multiple intricate indicators with microbial community data. Therefore, developing new R packages that are more suitable for drawing complex figures and composite figures would be necessary for microbiome data.

With the development of sequencing technology, data analysis methods have advanced along with the development of R packages contributed to the field of microbiome. These R packages range from classic R packages such as **vegan**, which has been cited more than 10,000 times, to integrated R packages such as **phyloseq**, which contain many functions in one package and set a unified data processing framework. These R packages have been able to implement most of the functions of microbiome analysis, from microbial diversity, difference, biomarker identification, correlation and network, phylogenetic analysis, etc. However, these R packages have some redundant functions; for example, **phyloseq**, **microbiome**, and others can do microbial diversity analysis. The difference is only in the visualization method and scheme. A similar situation has always existed in microbiome analysis R packages, so we hope that in future developments we will try to de-redundantly use the same part of

For Peer Review

the content or similar content to highlight the advantages of R packages.

Although these R packages can conduct a lot of functions, they don't do well enough in some specific analyses, for example, alpha and beta diversity analysis, and the outgoing graphs often do not add difference detection results to visualize the differences from the figures. In addition, there are still some contents that can continue to be developed, such as applying more machine learning methods to microbiome data and its learning method, model, and important variable evaluation. Secondly, metagenomes are becoming more widely used, and the support of species and functional annotation results based on Kraken (Wood and Salzberg, 2014), MEGAN (Huson et al., 2007), MetaPhlAn2 (Truong et al., 2015), HUMAnN2 (Franzosa et al., 2018), eggNOG-mapper (Huerta-Cepas et al., 2017), etc. is becoming more and more important, and these make the data processed by R rise from megabyte (M) to gigabyte (G). Therefore, Faster data processing R packages should be used to the microbiome data analysis process, such as **data.table**, **fst**, **tidyfst** etc.

The use of appropriate data structures can accelerate the microbiome data processing process. At first, we used S4 class objects for microbiome data encapsulation, which can complete a variety of analyses comprehensively and efficiently. The emergence of S6 class objects and other objects has greatly impacted microbiome data processing and largely facilitates it. With the development of the tidy family of R languages, tidy-based data structures have recently emerged for microbiome data mining. For example, the **MicrobiotaProcess** package (Xu et al., 2023). This structure is more suitable for microbiome data mining, machine learning

For Peer Review

modeling, and other analyses, which can more easily extract the influence of experimental design, time, space, and other factors on microbiome data in analysis, to discover the deep-seated patterns. We expect the R language to make microbiome analysis more efficient and help everyone discover more about its role in humans, animals, plants, and the environment, and use it for our benefit to make the world a better place.

Supplementary information

The online version contains supplementary figure 1-12 available at https://doi.org/10.1093/xxx.

Declarations

The authors declare no competing interests related to the content of this paper.

Author contributions

TW, G.N, J.Y and Y.L: conceived the study, and wrote the paper; JY and Y.L, conceived the study, and supervised the study; Y.L T.C and Q.S: provided critical comments on the study, and helped write the paper.

Acknowledgments

We thank all the people start and fork this project in GitHub and feedback the useful comments for the scripts.

Abbreviations

PCA, principal components analysis; NMDS, non-metric multidimensional scaling; DCA, decision curve analysis; CCA, canonical correspondence analysis; LDA, linear

For Peer Review

discriminant analysis; TMM, trimmed mean of M-values; RLE, relative log expression; UQ, upper quartile; MED, Median of ratios method; CSS, cumulative-sum scaling.

Funding

This study was financially supported by the Natural Science Foundation of China (42277297, 42090060), Jiangsu Funding Program for Excellent Postdoctoral Talent (2022ZB325), Scientific and technology innovation project of China Academy of Chinese Medical Sciences (C12021A04115), the Fundamental Research Funds for the Central public welfare research institutes (ZZ13-YQ-095).

For Peer Review

Data availability

No new sequencing data generated by this project. All the demo data and scripts are available in GitHub: https://github.com/taowenmicro/EasyMicrobiomeR.

References

Amir, A., McDonald, D., Navas-Molina, J.A., Kopylova, E., Morton, J.T., Zech Xu, Z., Kightley, E.P., Thompson, L.R., Hyde, E.R., and Gonzalez, A. (2017). Deblur rapidly resolves single-nucleotide community sequence patterns. MSystems 2, e00191-00116.

Aßhauer, K.P., Wemheuer, B., Daniel, R., and Meinicke, P. (2015). Tax4Fun: predicting functional profiles from metagenomic 16S rRNA data. Bioinformatics 31, 2882-2884.

Barnett, D.J., Arts, I.C., and Penders, J. (2021). microViz: an R package for microbiome data visualization and statistics. Journal of Open Source Software 6, 3201.

Bolyen, E., Rideout, J.R., Dillon, M.R., Bokulich, N.A., Abnet, C.C., Al-Ghalith, G.A., Alexander, H., Alm, E.J., Arumugam, M., and Asnicar, F. (2019). Reproducible, interactive, scalable and extensible microbiome data science using QIIME 2. Nature biotechnology 37, 852-857.

593 Callahan, B.J., McMurdie, P.J., Rosen, M.J., Han, A.W., Johnson, A.J.A., and Holmes, S.P.
594 (2016). DADA2: High-resolution sample inference from Illumina amplicon data. Nature methods
595 13, 581-583.
596 Caporaso, J.G., Kuczynski, J., Stombaugh, J., Bittinger, K., Bushman, F.D., Costello, E.K.,
597 Fierer, N., Peña, A.G., Goodrich, J.K., and Gordon, J.I. (2010). QIIME allows analysis of high-
598 throughput community sequencing data. Nature methods 7, 335-336.
599 Carrión, V.J., Perez-Jaramillo, J., Cordovez, V., Tracanna, V., De Hollander, M., Ruiz-Buck, D.,
600 Mendes, L.W., van Ijcken, W.F., Gomez-Exposito, R., and Elsayed, S.S. (2019). Pathogen-
601 induced activation of disease-suppressive functions in the endophytic root microbiome. Science
602 366, 606-612.
603 Chen, H., and Boutros, P.C. (2011). VennDiagram: a package for the generation of highly-
604 customizable Venn and Euler diagrams in R. BMC bioinformatics 12, 1-7.
605 Chen, Y., Li, J., Zhang, Y., Zhang, M., Sun, Z., Jing, G., Huang, S., and Su, X. (2022). Parallel-
606 Meta Suite: Interactive and rapid microbiome data analysis on multiple platforms. IMeta 1, e1.
607 Chen, Y., McCarthy, D., Robinson, M., and Smyth, G.K. (2014). edgeR: differential expression
608 analysis of digital gene expression data User's Guide. Bioconductor User's Guide.
609 Chong, J., Liu, P., Zhou, G., and Xia, J. (2020). Using MicrobiomeAnalyst for comprehensive
610 statistical, functional, and meta-analysis of microbiome data. Nature Protocols 15, 799-821.
611 Conway, J.R., Lex, A., and Gehlenborg, N. (2017). UpSetR: an R package for the visualization
612 of intersecting sets and their properties. Bioinformatics.
613 Dimitriadou, E., Hornik, K., Leisch, F., Meyer, D., and Weingessel, A. (2008). Misc functions of
614 the Department of Statistics (e1071), TU Wien. R package 1, 5-24.

For Peer Review

Dray, S., Bauman, D., Blanchet, G., Borcard, D., Clappe, S., Guenard, G., Jombart, T., Larocque, G., Legendre, P., and Madi, N. (2019). adespatial: Multivariate multiscale spatial analysis. R package version 0.3-7. Ecological Monographs 82.

Dray, S., and Dufour, A.-B. (2007). The ade4 package: implementing the duality diagram for ecologists. Journal of statistical software 22, 1-20.

Edgar, R.C. (2010). Search and clustering orders of magnitude faster than BLAST. Bioinformatics 26, 2460-2461.

Edgar, R.C. (2016). UNOISE2: improved error-correction for Illumina 16S and ITS amplicon sequencing. BioRxiv, 081257.

Fisher, R.A. (1936). The use of multiple measurements in taxonomic problems. Annals of eugenics 7, 179-188.

Franzosa, E.A., McIver, L.J., Rahnavard, G., Thompson, L.R., Schirmer, M., Weingart, G., Lipson, K.S., Knight, R., Caporaso, J.G., and Segata, N. (2018). Species-level functional profiling of metagenomes and metatranscriptomes. Nature methods 15, 962-968.

Gu, Z. (2022). Complex heatmap visualization. iMeta 1, e43.

Gu, Z., Gu, L., Eils, R., Schlesner, M., and Brors, B. (2014). circlize implements and enhances circular visualization in R. Bioinformatics 30, 2811-2812.

Hamilton, N.E., and Ferry, M. (2018). ggtern: Ternary diagrams using ggplot2. Journal of Statistical Software 87, 1-17.

Harrell Jr, F.E., and Harrell Jr, M.F.E. (2019). Package 'hmisc'. CRAN2018 2019, 235-236.

Hofner, B., Mayr, A., Robinzonov, N., and Schmid, M. (2014). Model-based boosting in R: a hands-on tutorial using the R package mboost. Computational statistics 29, 3-35.

For Peer Review

Huerta-Cepas, J., Forslund, K., Coelho, L.P., Szklarczyk, D., Jensen, L.J., Von Mering, C., and Bork, P. (2017). Fast genome-wide functional annotation through orthology assignment by eggNOG-mapper. Molecular biology evolution 34, 2115-2122.

Huson, D.H., Auch, A.F., Qi, J., and Schuster, S.C. (2007). MEGAN analysis of metagenomic data. Genome research 17, 377-386.

Ihaka, R., and Gentleman, R. (1996). R: a language for data analysis and graphics. Journal of computational graphical statistics 5, 299-314.

Kembel, S.W., Cowan, P.D., Helmus, M.R., Cornwell, W.K., Morlon, H., Ackerly, D.D., Blomberg, S.P., and Webb, C.O. (2010). Picante: R tools for integrating phylogenies and ecology. Bioinformatics 26, 1463-1464.

Knights, D., Kuczynski, J., Charlson, E.S., Zaneveld, J., Mozer, M.C., Collman, R.G., Bushman, F.D., Knight, R., and Kelley, S.T. (2011). Bayesian community-wide culture-independent microbial source tracking. Nature methods 8, 761-763.

Kolde, R. (2012). Pheatmap: pretty heatmaps. R package version 1, 726.

Kuhn, M. (2009). The caret package. Journal of Statistical Software 28.

Kurtz, Z.D., Müller, C.L., Miraldi, E.R., Littman, D.R., Blaser, M.J., and Bonneau, R.A. (2015). Sparse and compositionally robust inference of microbial ecological networks. PLoS computational biology 11, e1004226.

Langfelder, P., and Horvath, S. (2008). WGCNA: an R package for weighted correlation network analysis. BMC Bioinformatics 9, 1-13.

Liaw, A., and Wiener, M. (2002). Classification and regression by randomForest. R news 2,

For Peer Review

18-22.

Lin, H., and Peddada, S.D. (2020). Analysis of microbial compositions: a review of normalization and differential abundance analysis. NPJ biofilms and microbiomes 6, 1-13.

Liu, C., Cui, Y., Li, X., and Yao, M. (2021a). microeco: an R package for data mining in microbial community ecology. FEMS microbiology ecology 97, fiaa255.

Liu, Y., Qin, Y., Chen, T., Lu, M., Qian, X., Guo, X., and Bai, Y. (2021b). A practical guide to amplicon and metagenomic analysis of microbiome data. Protein & cell 12, 315-330.

Liu, Y.X., Chen, L., Ma, T., Li, X., Zheng, M., Zhou, X., Chen, L., Qian, X., Xi, J., and Lu, H. (2023). EasyAmplicon: An easy-to-use, open-source, reproducible, and community-based pipeline for amplicon data analysis in microbiome research. iMeta, e83.

Louca, S., Parfrey, L.W., and Doebeli, M. (2016). Decoupling function and taxonomy in the global ocean microbiome. Science 353, 1272-1277.

Love, M.I., Huber, W., and Anders, S. (2014). Moderated estimation of fold change and dispersion for RNA-seq data with DESeq2. Genome biology 15, 1-21.

McMurdie, P.J., and Holmes, S. (2013). phyloseq: an R package for reproducible interactive analysis and graphics of microbiome census data. PloS one 8, e61217.

Metcalf, J.L., Xu, Z.Z., Weiss, S., Lax, S., Van Treuren, W., Hyde, E.R., Song, S.J., Amir, A., Larsen, P., and Sangwan, N. (2016). Microbial community assembly and metabolic function during mammalian corpse decomposition. Science 351, 158-162.

Nearing, J.T., Douglas, G.M., Hayes, M.G., MacDonald, J., Desai, D.K., Allward, N., Jones, C.M.A., Wright, R.J., Dhanani, A.S., Comeau, A.M., *et al.* (2022). Microbiome differential abundance methods produce different results across 38 datasets. Nature Communications 13,

For Peer Review

681 342.

682 Nguyen, N.H., Song, Z., Bates, S.T., Branco, S., Tedersoo, L., Menke, J., Schilling, J.S., and
683 Kennedy, P.G. (2016). FUNGuild: an open annotation tool for parsing fungal community
684 datasets by ecological guild. Fungal Ecology 20, 241-248.

685 Ning, D., Yuan, M., Wu, L., Zhang, Y., Guo, X., Zhou, X., Yang, Y., Arkin, A.P., Firestone, M.K.,
686 and Zhou, J. (2020). A quantitative framework reveals ecological drivers of grassland microbial
687 community assembly in response to warming. Nature communications 11, 4717.

688 Oksanen, J., Kindt, R., Legendre, P., O'Hara, B., Stevens, M.H.H., Oksanen, M.J., and
689 Suggests, M. (2007). The vegan package. Community ecology package 10, 719.

690 Pages, H., Aboyoun, P., Gentleman, R., and DebRoy, S. (2016). Biostrings: String objects
691 representing biological sequences, and matching algorithms. R package version 2, 10.18129.

692 Paoli, L., Ruscheweyh, H.-J., Forneris, C.C., Hubrich, F., Kautsar, S., Bhushan, A., Lotti, A.,
693 Clayssen, Q., Salazar, G., Milanese, A., *et al.* (2022). Biosynthetic potential of the global ocean
694 microbiome. Nature 607, 111-118.

695 Pasolli, E., Schiffer, L., Manghi, P., Renson, A., Obenchain, V., Truong, D.T., Beghini, F., Malik,
696 F., Ramos, M., Dowd, J.B., *et al.* (2017). Accessible, curated metagenomic data through
697 ExperimentHub. Nature Methods 14, 1023-1024.

698 Proctor, L.M., Creasy, H.H., Fettweis, J.M., Lloyd-Price, J., Mahurkar, A., Zhou, W., Buck, G.A.,
699 Snyder, M.P., Strauss, J.F., Weinstock, G.M., *et al.* (2019). The Integrative Human Microbiome
700 Project. Nature 569, 641-648.

701 Revelle, W., and Revelle, M.W. (2015). Package 'psych'. The comprehensive R archive
702 network 337, 338.

For Peer Review

703 Ripley, B., Venables, B., Bates, D.M., Hornik, K., Gebhardt, A., Firth, D., and Ripley, M.B.
704 (2013). Package 'mass'. Cran r 538, 113-120.
705 Robin, X., Turck, N., Hainard, A., Tiberti, N., Lisacek, F., Sanchez, J.-C., and Müller, M. (2011).
706 pROC: an open-source package for R and S+ to analyze and compare ROC curves. BMC
707 bioinformatics 12, 1-8.
708 Rognes, T., Flouri, T., Nichols, B., Quince, C., and Mahé, F. (2016). VSEARCH: a versatile
709 open source tool for metagenomics. PeerJ 4, e2584.
710 Schloss, P.D., Westcott, S.L., Ryabin, T., Hall, J.R., Hartmann, M., Hollister, E.B., Lesniewski,
711 R.A., Oakley, B.B., Parks, D.H., and Robinson, C.J. (2009). Introducing mothur: open-source,
712 platform-independent, community-supported software for describing and comparing microbial
713 communities. Applied and environmental microbiology 75, 7537-7541.
714 Shenhav, L., Thompson, M., Joseph, T.A., Briscoe, L., Furman, O., Bogumil, D., Mizrahi, I.,
715 Pe'er, I., and Halperin, E. (2019). FEAST: fast expectation-maximization for microbial source
716 tracking. Nature methods 16, 627-632.
717 Si, B., Liang, Y., Zhao, J., Zhang, Y., Liao, X., Jin, H., Liu, H., and Gu, L. (2020). Ggraph: An
718 efficient structure-aware approach for iterative graph processing. IEEE Transactions on Big
719 Data.
720 Smyth, G.K. (2005). Limma: linear models for microarray data. In Bioinformatics and
721 computational biology solutions using R and Bioconductor (Springer), pp. 397-420.
722 Stegen, J.C., Lin, X., Fredrickson, J.K., Chen, X., Kennedy, D.W., Murray, C.J., Rockhold, M.L.,
723 and Konopka, A. (2013). Quantifying community assembly processes and identifying features
724 that impose them. The ISME journal 7, 2069-2079.

For Peer Review

Thompson, L.R., Sanders, J.G., McDonald, D., Amir, A., Ladau, J., Locey, K.J., Prill, R.J., Tripathi, A., Gibbons, S.M., Ackermann, G., *et al.* (2017). A communal catalogue reveals Earth's multiscale microbial diversity. Nature 551, 457-463.

Truong, D.T., Franzosa, E.A., Tickle, T.L., Scholz, M., Weingart, G., Pasolli, E., Tett, A., Huttenhower, C., and Segata, N. (2015). MetaPhlAn2 for enhanced metagenomic taxonomic profiling. Nature methods 12, 902-903.

Wemheuer, F., Taylor, J.A., Daniel, R., Johnston, E., Meinicke, P., Thomas, T., and Wemheuer, B. (2020). Tax4Fun2: prediction of habitat-specific functional profiles and functional redundancy based on 16S rRNA gene sequences. Environmental Microbiome 15, 1-12.

Wen, T., Xie, P., Yang, S., Niu, G., Liu, X., Ding, Z., Xue, C., Liu, Y.X., Shen, Q., and Yuan, J. (2022). ggClusterNet: An R package for microbiome network analysis and modularity-based multiple network layouts. iMeta 1, e32.

Wickham, H. (2011). ggplot2. Wiley interdisciplinary reviews: computational statistics 3, 180-185.

Wickham, H. (2012). reshape2: Flexibly reshape data: a reboot of the reshape package. R package version 1.

Wickham, H., Francois, R., Henry, L., and Müller, K. (2014). dplyr. Paper presented at: useR! Conference.

Wickham, H., and Wickham, M.H. (2020). Package 'plyr'. A Grammar of Data Manipulation R package version 8.

Wirbel, J., Zych, K., Essex, M., Karcher, N., Kartal, E., Salazar, G., Bork, P., Sunagawa, S., and Zeller, G. (2021). Microbiome meta-analysis and cross-disease comparison enabled by the

For Peer Review

747 SIAMCAT machine learning toolbox. Genome Biology 22, 93.

748 Wood, D.E., and Salzberg, S.L. (2014). Kraken: ultrafast metagenomic sequence classification
749 using exact alignments. Genome biology 15, 1-12.

750 Xu, S., Li, L., Luo, X., Chen, M., Tang, W., Zhan, L., Dai, Z., Lam, T.T., Guan, Y., and Yu, G.
751 (2022). Ggtree: A serialized data object for visualization of a phylogenetic tree and annotation
752 data. iMeta 1, e56.

753 Xu, S., Zhan, L., Tang, W., Wang, Q., Dai, Z., Zhou, L., Feng, T., Chen, M., Wu, T., and Hu, E.
754 (2023). MicrobiotaProcess: A comprehensive R package for deep mining microbiome. The
755 Innovation.

756 Zhao, Y., Federico, A., Faits, T., Manimaran, S., Segrè, D., Monti, S., and Johnson, W.E. (2021).
757 animalcules: interactive microbiome analytics and visualization in R. Microbiome 9, 1-16.

For Peer Review

Table.1 Comparison of the advantages and limitations of the six integrated R packages

R package	Function	Advantages	Limitations
phyloseq	1. Diversity analysis including alpha / beta diversity, community composition, and phylogenetic tree analysis.<br>2. Network analysis.	1. Firstly utilize S4 class objects.<br>2. Possess a set of data processing and analysis functions based on phyloseq objects.<br>3. Combine evolutionary trees with microbial abundance to display species richness (Fig.S2G).<br>4. Ordinate analysis can be applied to arrange the order of samples and microbes on heatmap rows and columns (Fig.S2F).<br>5. The network analysis process is simplified (Fig.S2E).<br>6. Offer over 30 distance algorithms.	1. Introduction to phyloseq objects can be challenging for beginners.<br>2. Statistical tests, including diversity tests and community/feature-level microbial difference analysis, are not well integrated into community analysis.<br>3. Network analysis lacks test, attribute calculation.
microbiome	1. Diversity analysis only including alpha / beta diversity, community composition).	1. The alpha diversity index is abundance.<br>2. The t-SNE and CAP ordination algorithms.<br>3. The stacked bar chart for community composition analysis can be sorted by specified microbial features (Fig.S3C).<br>4. Visualization of individual microbes (Fig.S2D).	1. The t-SNE and CAP ordination analyses frequently encounter errors.<br>2. The statistical tests, including diversity tests, community and feature-level differences tests is not ideal.
MicrobiomeAnalystR	1. Diversity analysis including alpha/beta diversity, community composition, and phylogenetic tree analysis.<br>2. Difference analysis.<br>3. Biomarker-based diagnosis.	1. Comprehensive workflow with various functions ranging from data cleaning to visualization.<br>2. Multiple algorithms to correct sequencing errors, leading more accurate evaluation of abundance.<br>3. Various analyses can be performed at different taxonomic levels (Fig.S2E).<br>4. Machine learning can be utilized to search for and extract feature variables (Fig.S2G).<br>5. Difference analysis can be conducted using multiple methods	1. Difficulties in installing R packages with dependencies.<br>2. Some functions may not work, including network analysis and difference analysis of relative abundance.<br>3. Insufficient explanation of parameters and examples.

For Peer Review

		such as LEfSe and metagenomeSeq.	
Animalcules	1. Sequence statistics visualization.<br>2. Diversity analysis including alpha/ beta diversity, community composition.<br>3. Difference analysis and biomarker identification.	1. The commonly used objects in omics analysis, such as SummarizedExperiment, can be utilized.<br>2. It can be interactively executed in R.<br>3. A 3D clustering plot can be generated.	1. Unable to save vector graphics and completed tables.<br>2. Insufficient functionality.
microeco	1. Diversity analysis including alpha / beta diversity, community composition, and phylogenetic tree analysis.<br>2. Difference analysis.<br>3. Biomarker identification.<br>4. Network analysis.<br>5. Correlation analysis with other indicators.<br>6. Functional prediction.	1. R6 class more expansibility than phyloseq objects.<br>2. Simple function calling.<br>3. Rich graphical representation of diversity and difference analysis results (Fig.S6A-G).<br>4. Unique correlation analysis of other indicators.<br>5. Abundant network analysis algorithms with comprehensive functionality (Fig.S6J).<br>6. FAPROTAX and FUNGuild function prediction.	1. New data structures increase the cost of learning time.<br>2. So many functions and dependency caused frequent some malfunctioning.
EasyAmplicon	1. Diversity analysis<br>2. Provide script for preparing STAMP, LEfSe, PICRUSt 1&2, BugBase, FAPROTAX, iTOL<br>3. Provide slide tutorial for each analysis and QIIME 2 pipeline	1. It can be used in both command-line mode and interactive mode within RStudio.<br>2. It offers multiple visualization styles, allowing for easy generation of publication-quality figures (Fig.S7).<br>3. Its open-source code facilitates reproducible analysis and allows for personalized modifications	1. Need using the most popular tools, STAMP, LEfSe, PICRUSt 1&2, BugBase, FAPROTAX, iTOL.<br>2. Some functions need to be development.

For Peer Review

Figures & Legends

Figure 1. Microbial community data analysis workflow and related R packages.

(A) Overview of microbial community data analysis workflow. Core files are feature table (OTU), Taxonomy, sample metadata (Metadata), phylogenetic tree (Tree), and representative sequences (Ref.fa). (B) Detail of microbial community analysis workflow. First, the raw data can be processed by using USEARCH/VSEARCH, QIIME 2, DADA2 packages. Then, the important files are saved and used for downstream analysis in R language and RStudio software. Many microbial analysis methods rely on numerous R packages developed with R language. The font size in the word cloud represents the number of citations of R packages. (C) Commonly used R packages for data manipulation and visualization. (D) Classification of R packages for six categories in microbial community analysis.

Figure 2. Introduction to the functions of integrated microbial analysis R packages.

Microbial community analysis can be divided into diversity analysis, difference analysis, biomarker identification, correlation and network analysis, functional prediction, and other microbial community analysis (community construction process, association analysis with other indicators).

Figure 3. Typical results of integrated microbial community analysis R packages and comparison of similar results.

Group the analysis results of multiple integrated R packages according to the major categories of microbial community analysis functions. Each main branch in the tree diagram represents a type of microbial community analysis, and there are a total of 10

For Peer Review

main branches: feature diversity analysis including 1 alpha diversity analysis, 2 beta diversity analysis, 3 community taxonomic or functional composition analysis, 4 evolutionary or taxonomic tree analysis; 5 difference analysis; 6 biomarker identification; 7 correlation and network analysis; 8 functional prediction; 9 community construction process analysis; 10 association analysis with other indicators. Each leaf (circle) represents a style of the result displayed in the analysis, and the circle number around the outside of leaf represents the package number of the integrated R package that the analysis result comes from.

For Peer Review

Figure 4. Examples of the best practice results of microbial community analysis in R language.

The selected results include rarefaction curve (A), Principal coordinate analysis scatter plot (B), Venn network graph (C), evolutionary tree (D), LEfSe cladogram (E), difference analysis STAMP style extended error bar plot (F), difference analysis Manhattan plot (G), difference analysis multi-group volcano plot (H), biomarker selection ring-column chart (I), network graph (J), correlation connection combination graph (K), source tracing analysis pie chart (L).

Figure 1

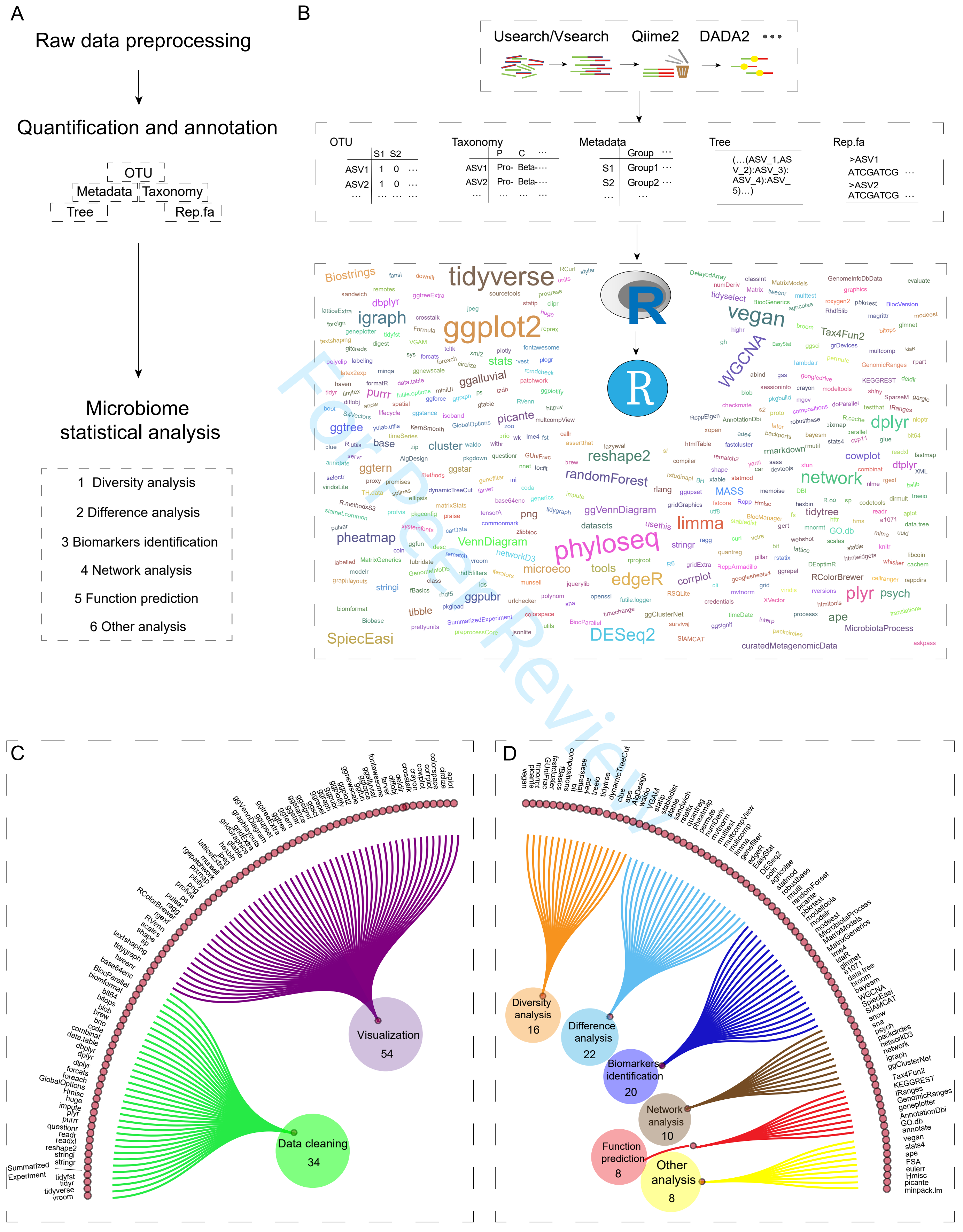

For Peer Review

Figure 2

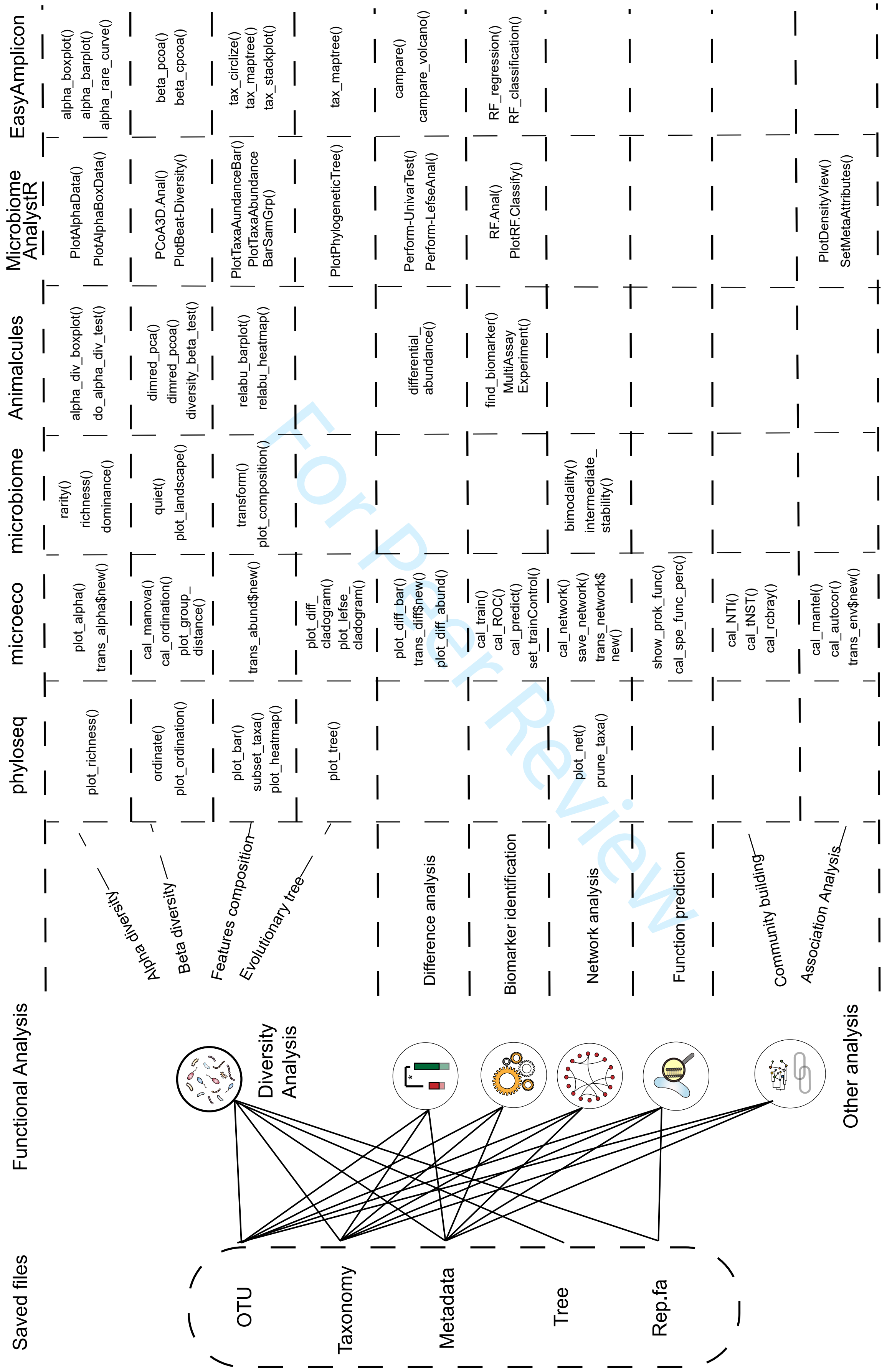

For Peer Review

Figure 3

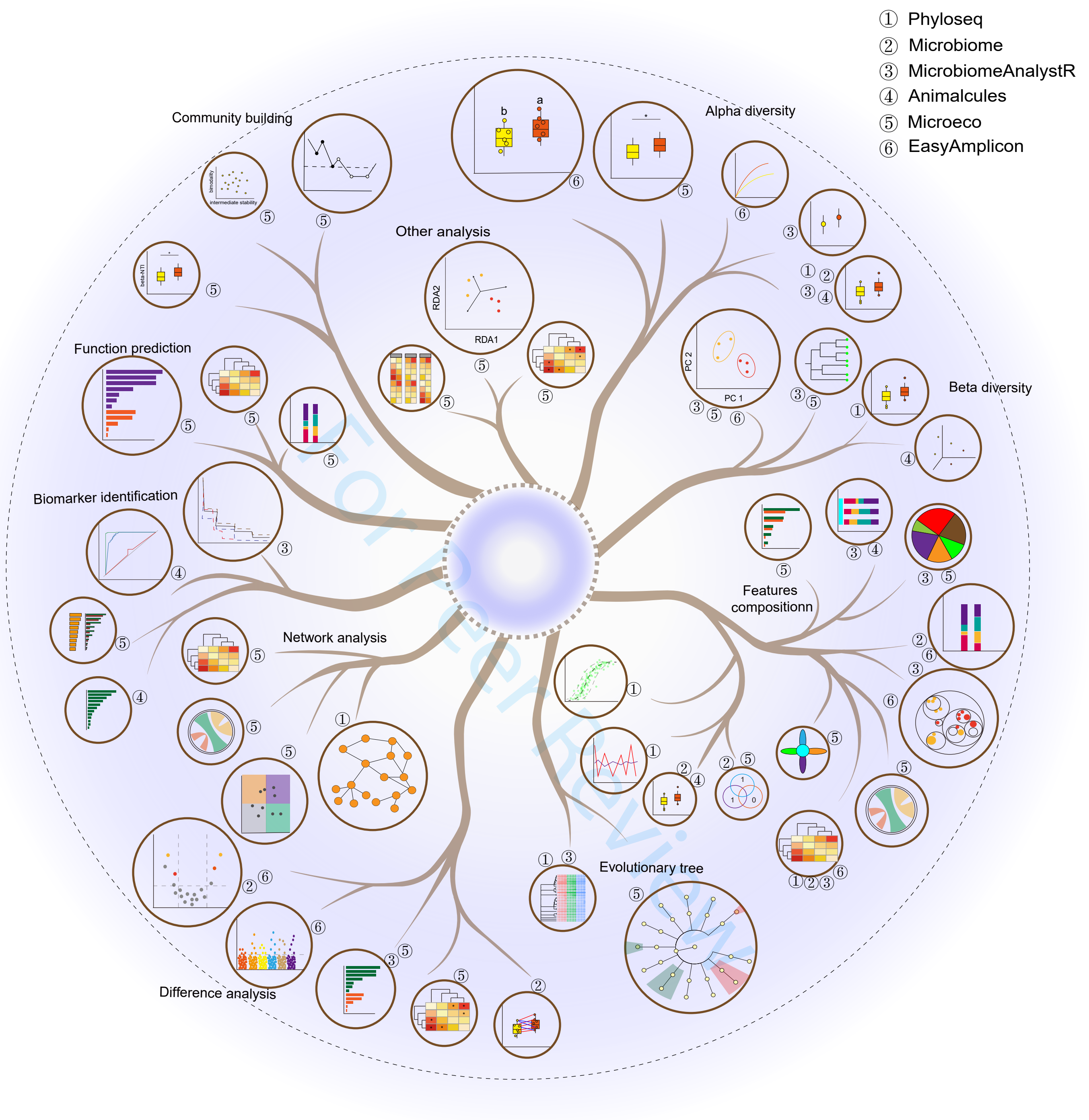

For Peer Review

Figure 4

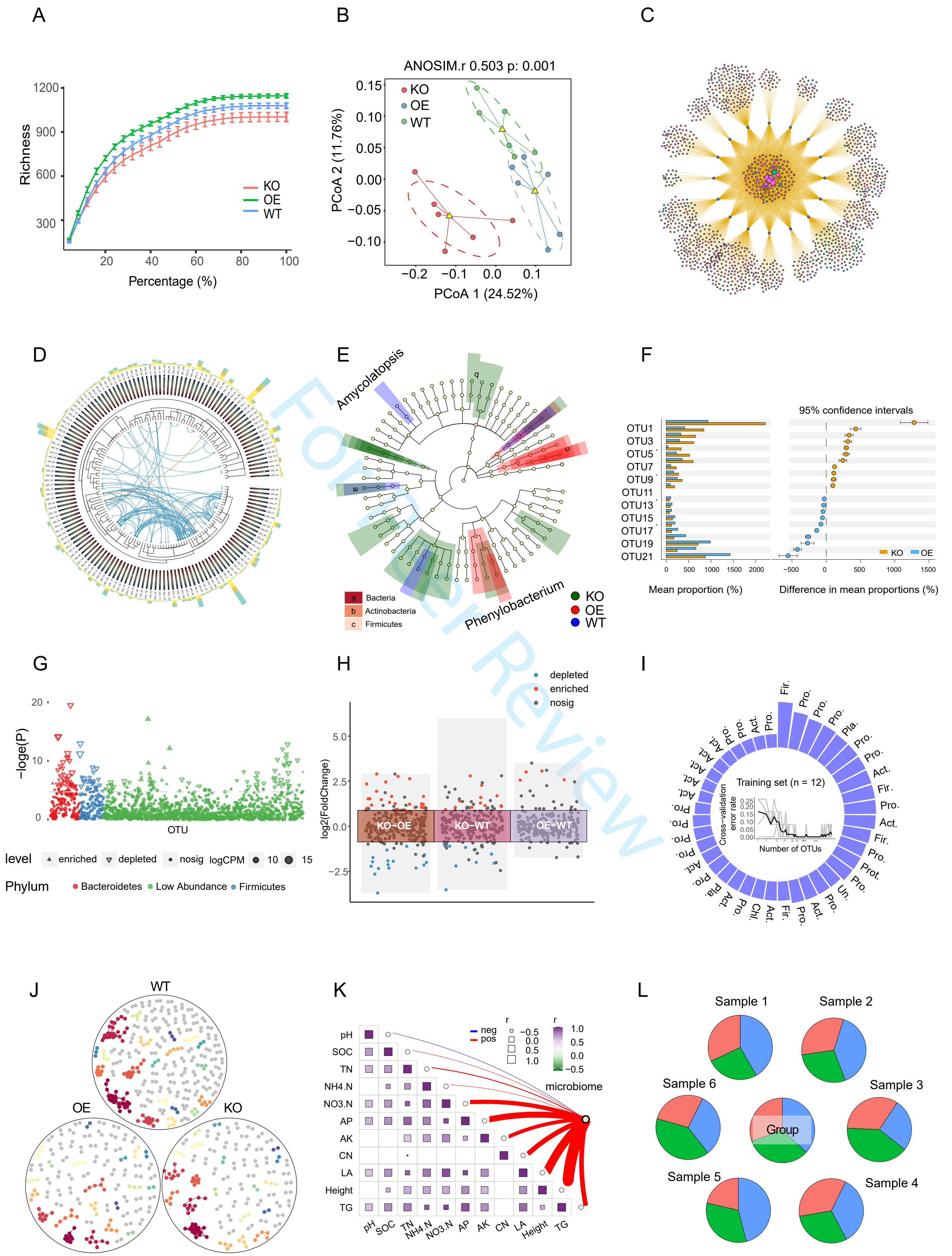

For Peer Review

Fig. S1 Showcases 9 specific categories of 324 R packages required for microbiome analysis. These packages have been classified into the following categories: dependent, data cleaning, visualization, diversity analysis, difference analysis, biomarker identification, correlation and network analysis, functional prediction, and other analysis (community construction process, association analysis with other indicators).

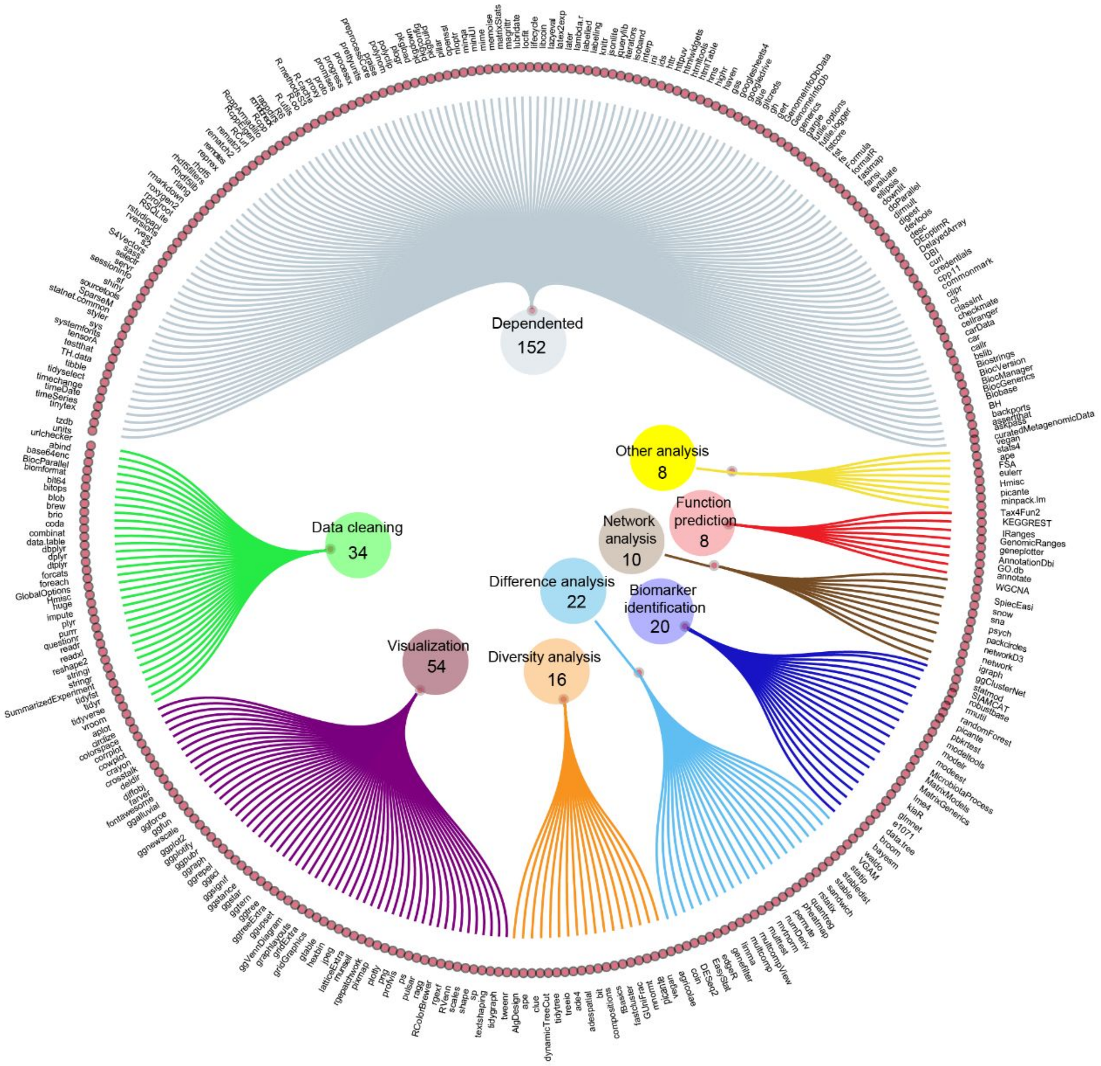

Fig. S2 Partial display of results from integrated R package phyloseq

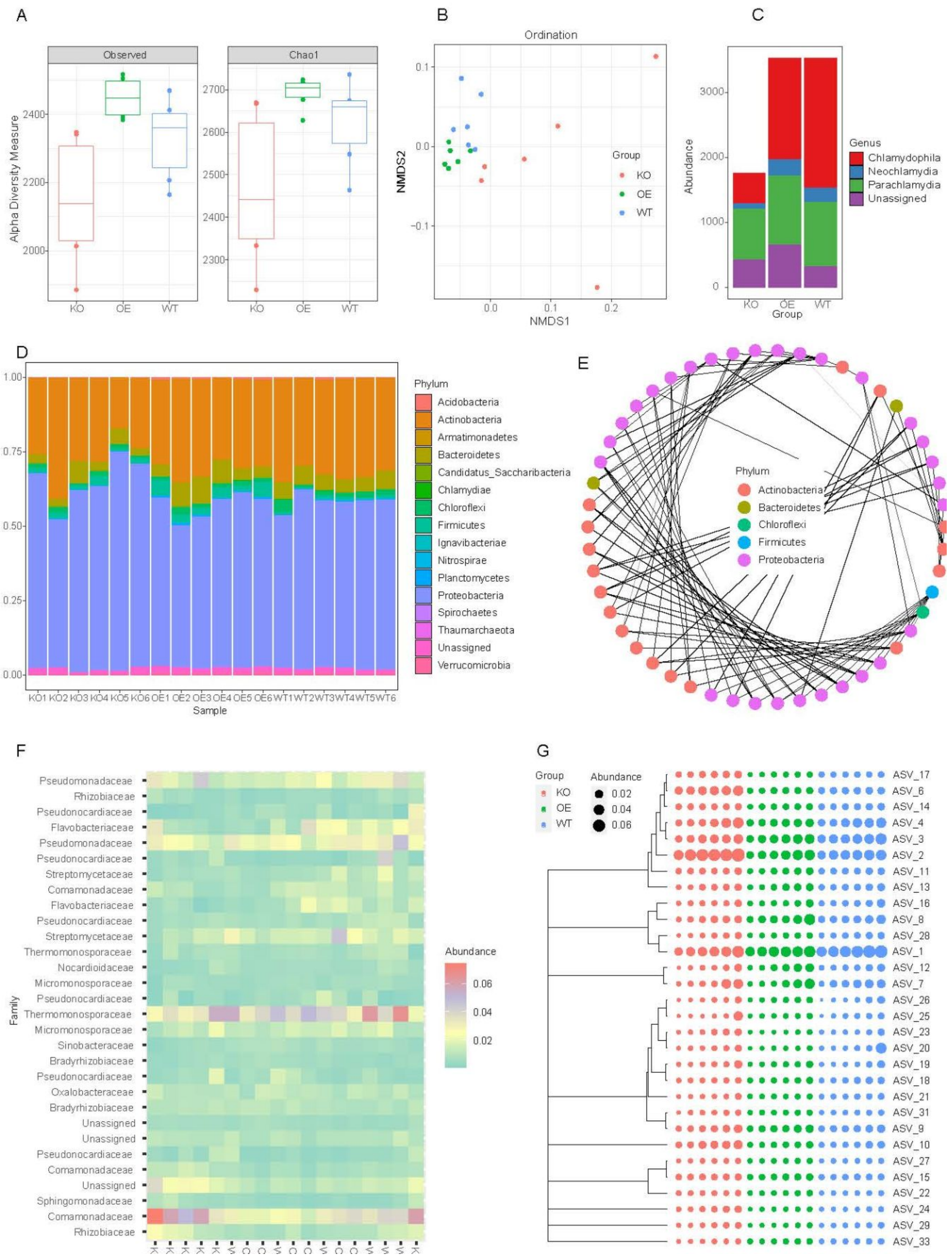

Fig. S3 Partial display of results from integrated R package microbiome

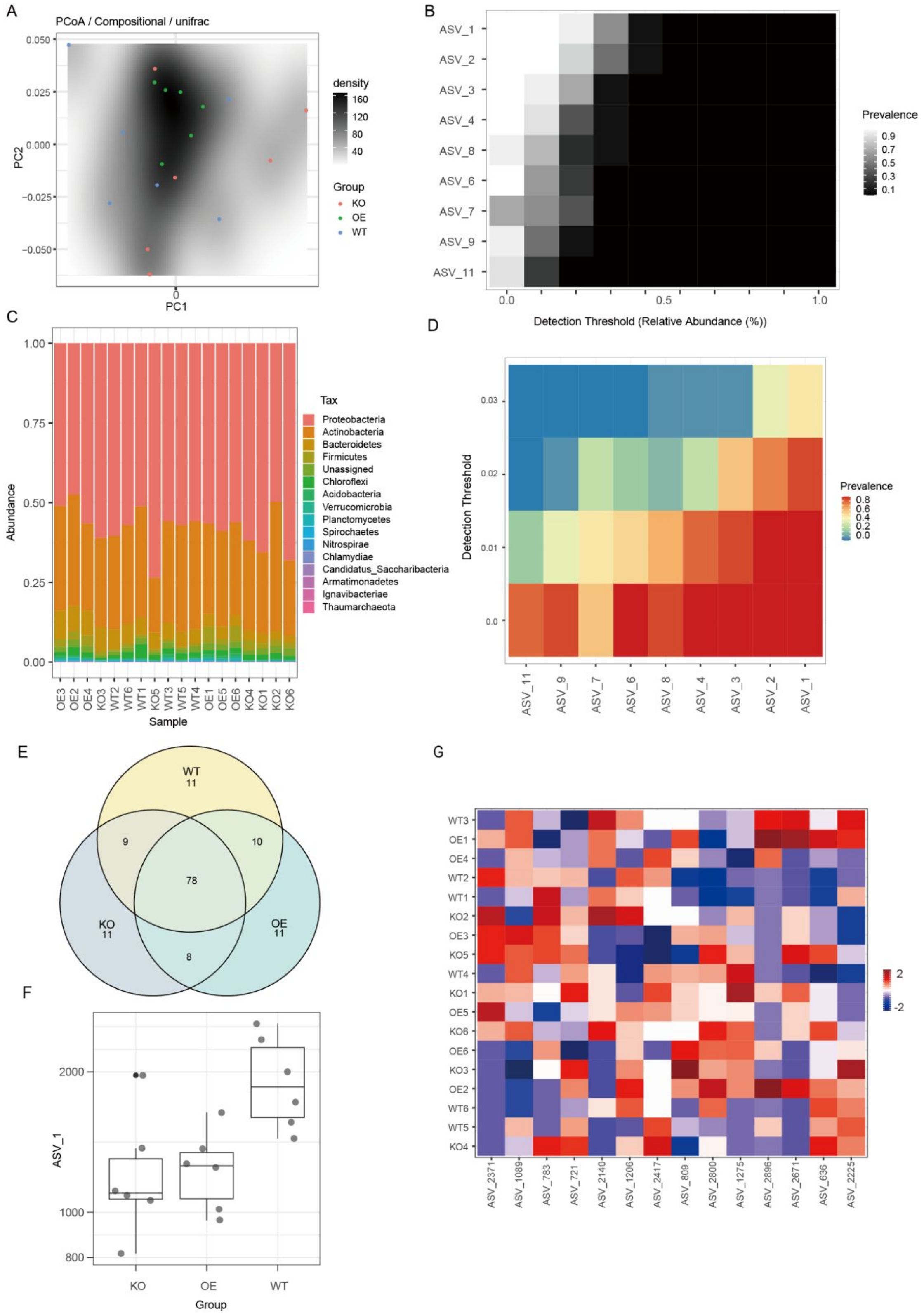

Fig. S4 Partial display of results from integrated R package MicrobiomeAnalystR

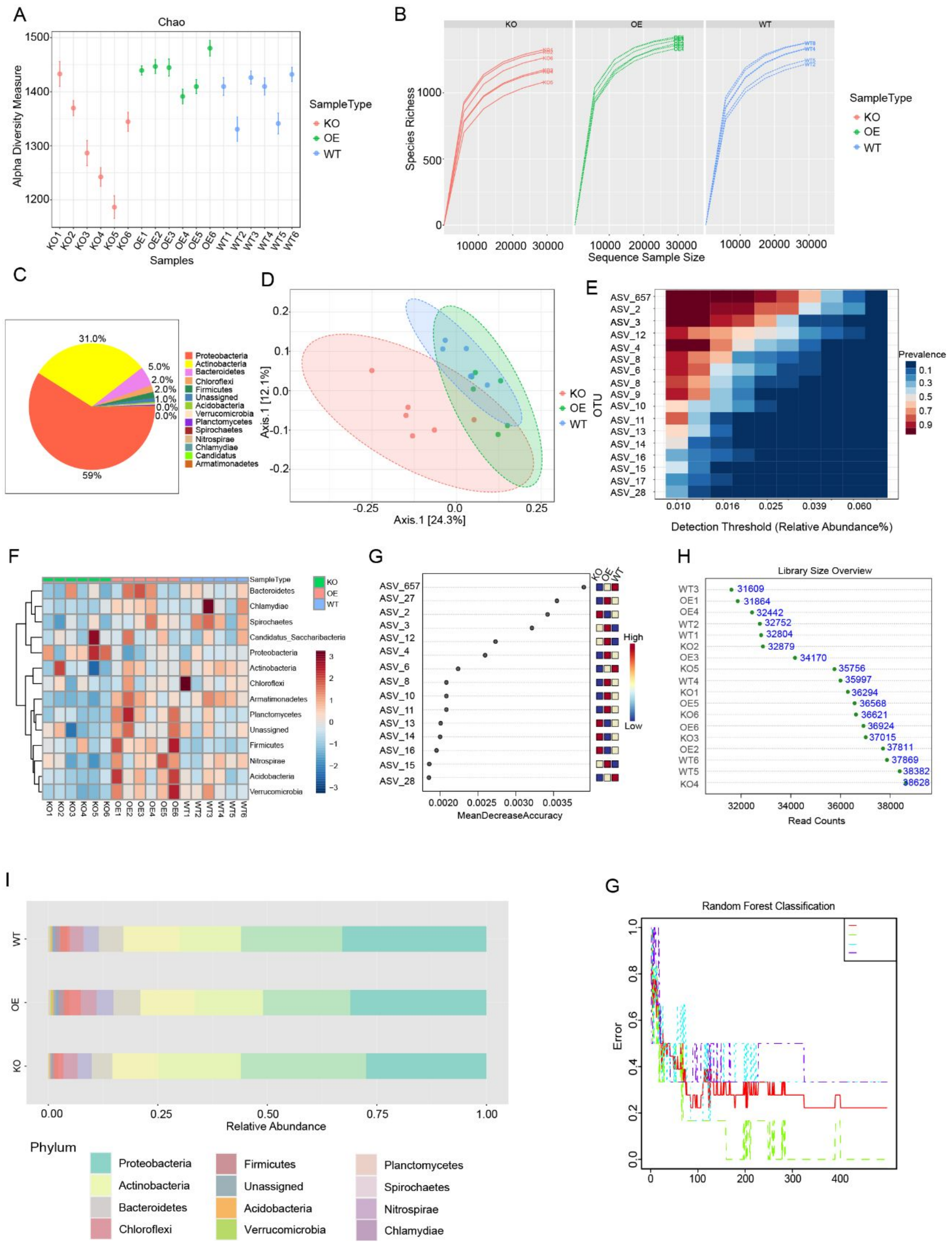

Fig. S5 Partial display of results from integrated R package Animalcules

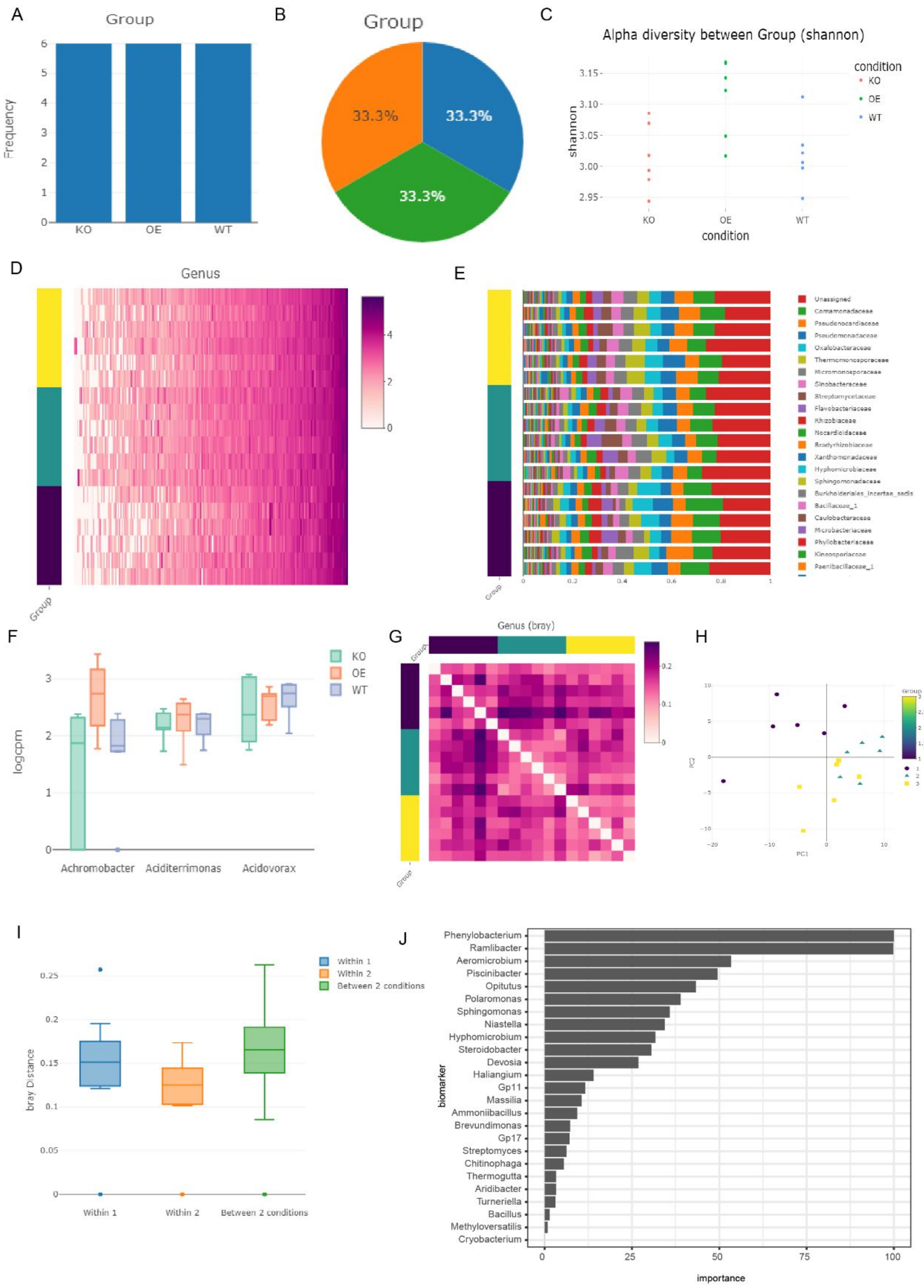

Fig. S6 Partial display of results from integrated R package microeco

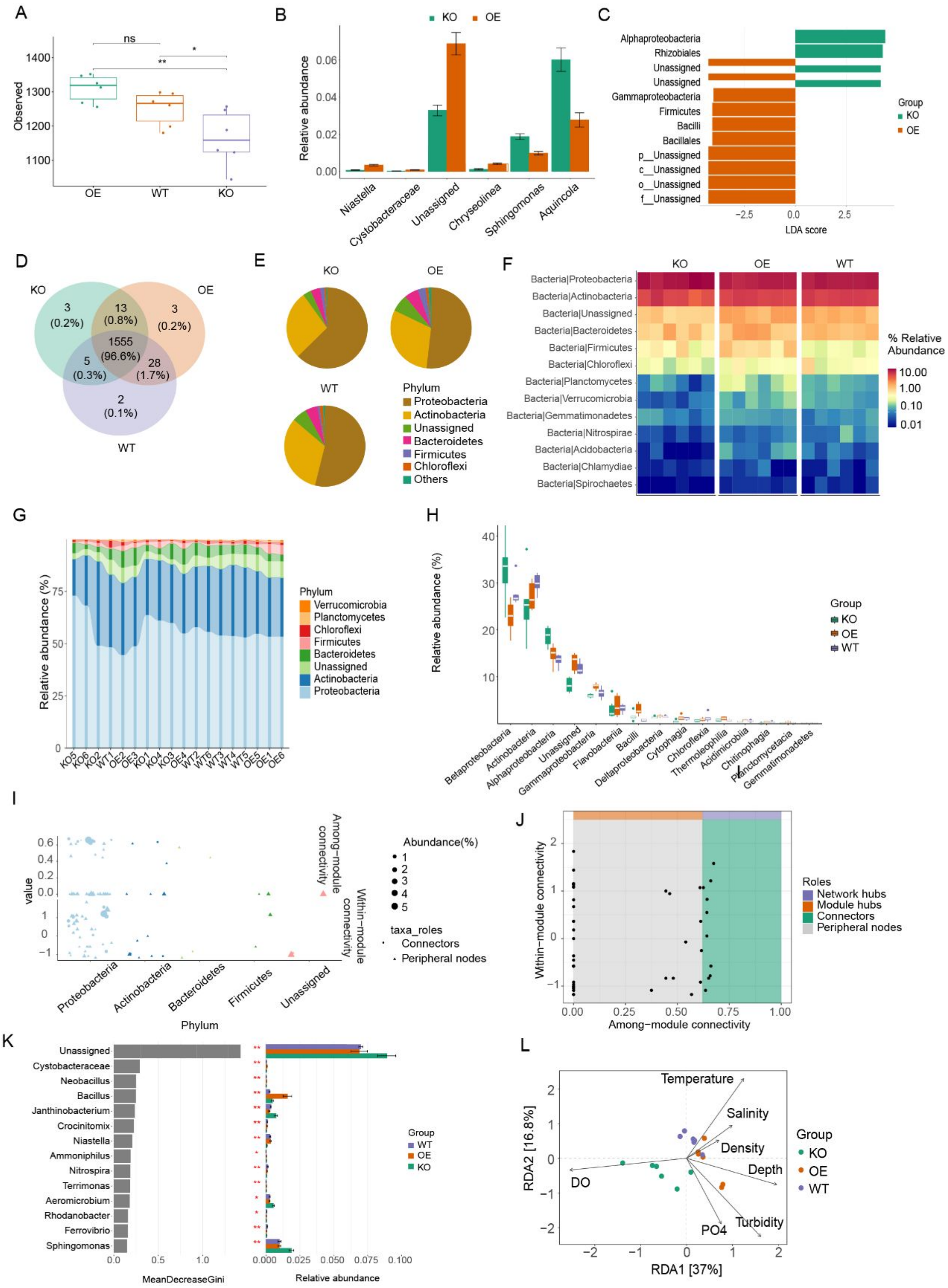

Fig. S7 Partial display of results from integrated R package amplicon

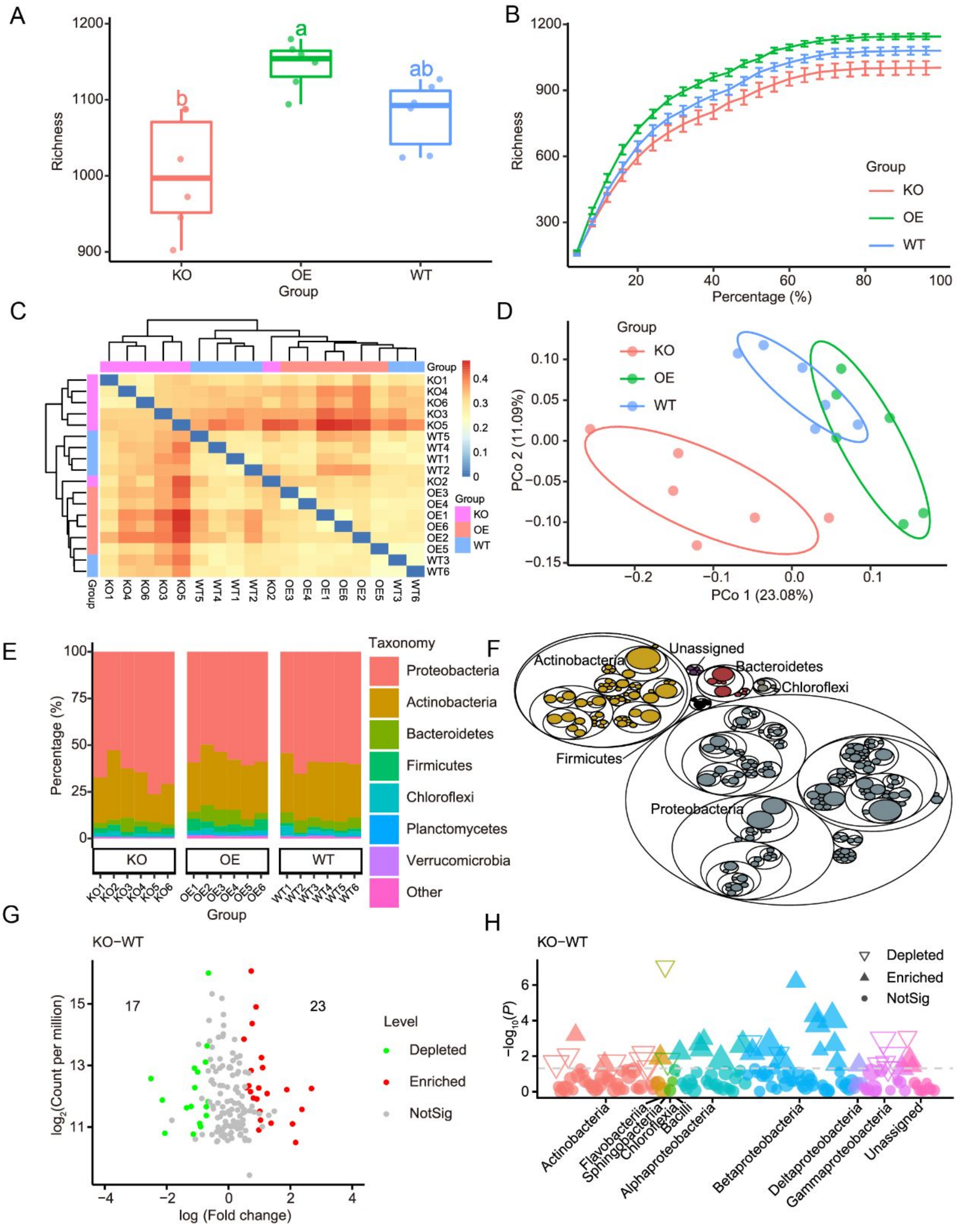

Fig. S8 Best practice analysis flow chart for microbiome data analysis

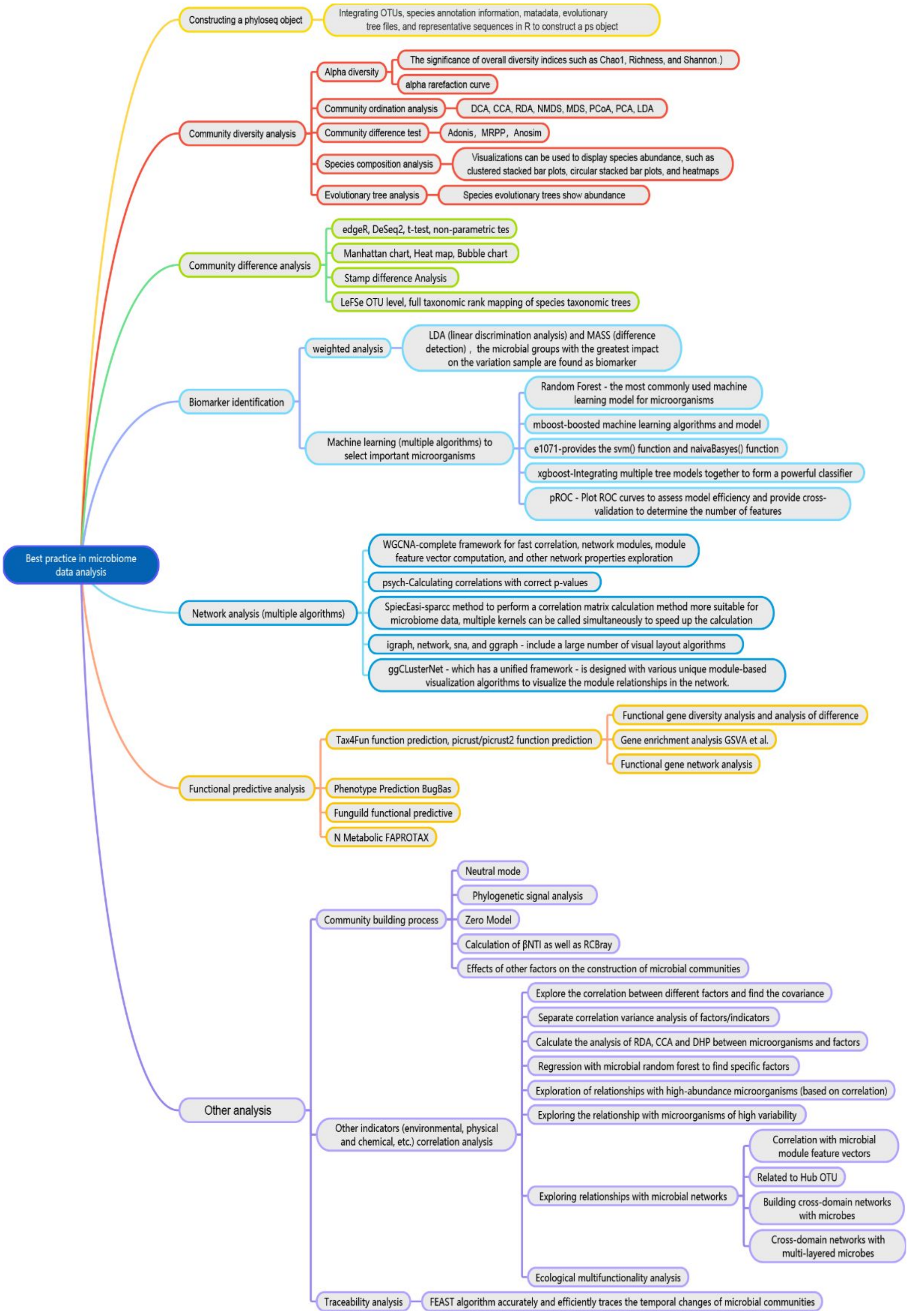

Fig. S9 Partial display of results from best practices in microbiome data analysis. (A-I) diversity analysis

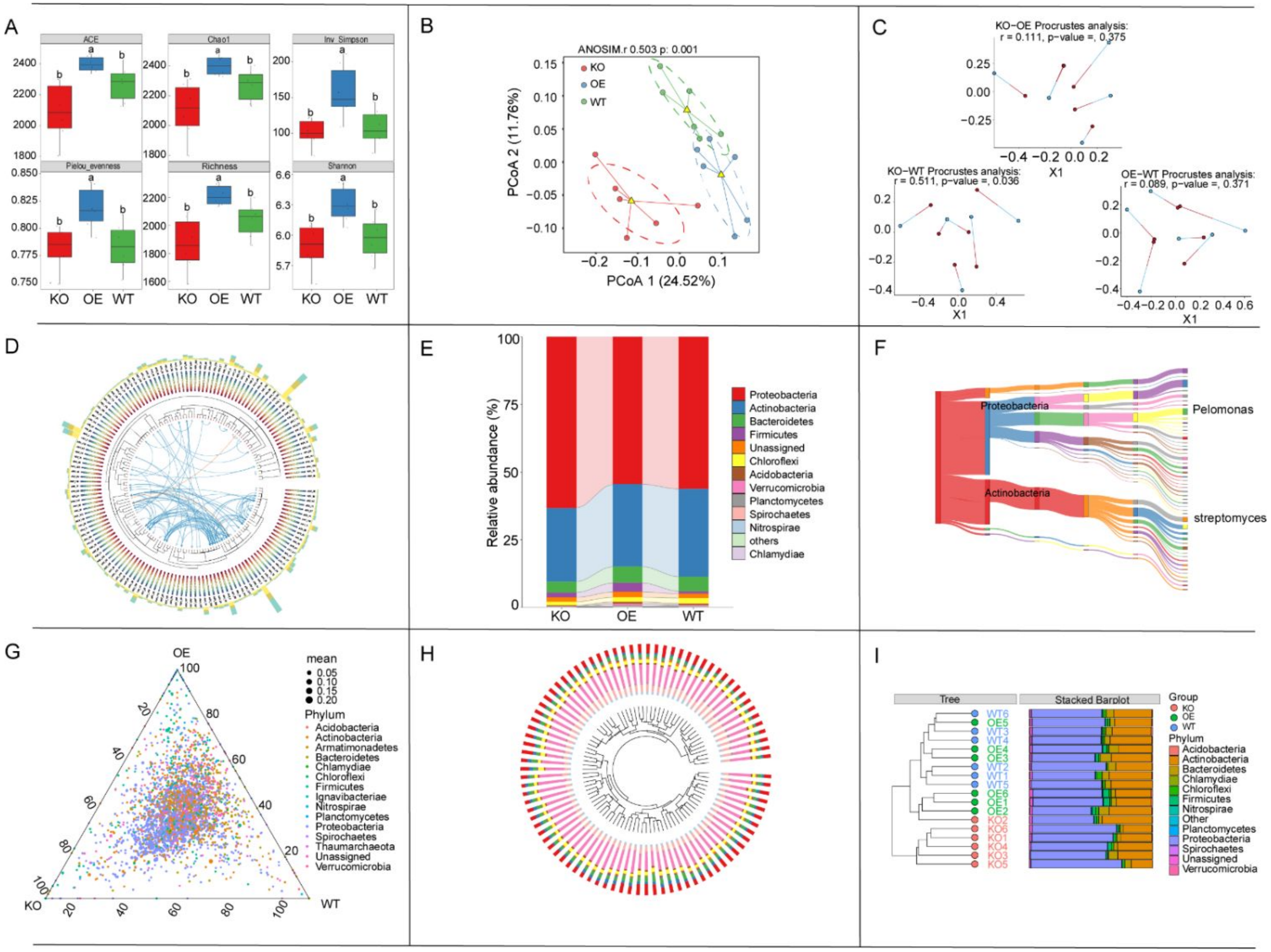

Fig. S10 Partial display of results from best practices in microbiome data analysis. (A-E) diversity analysis, (F-I) difference analysis

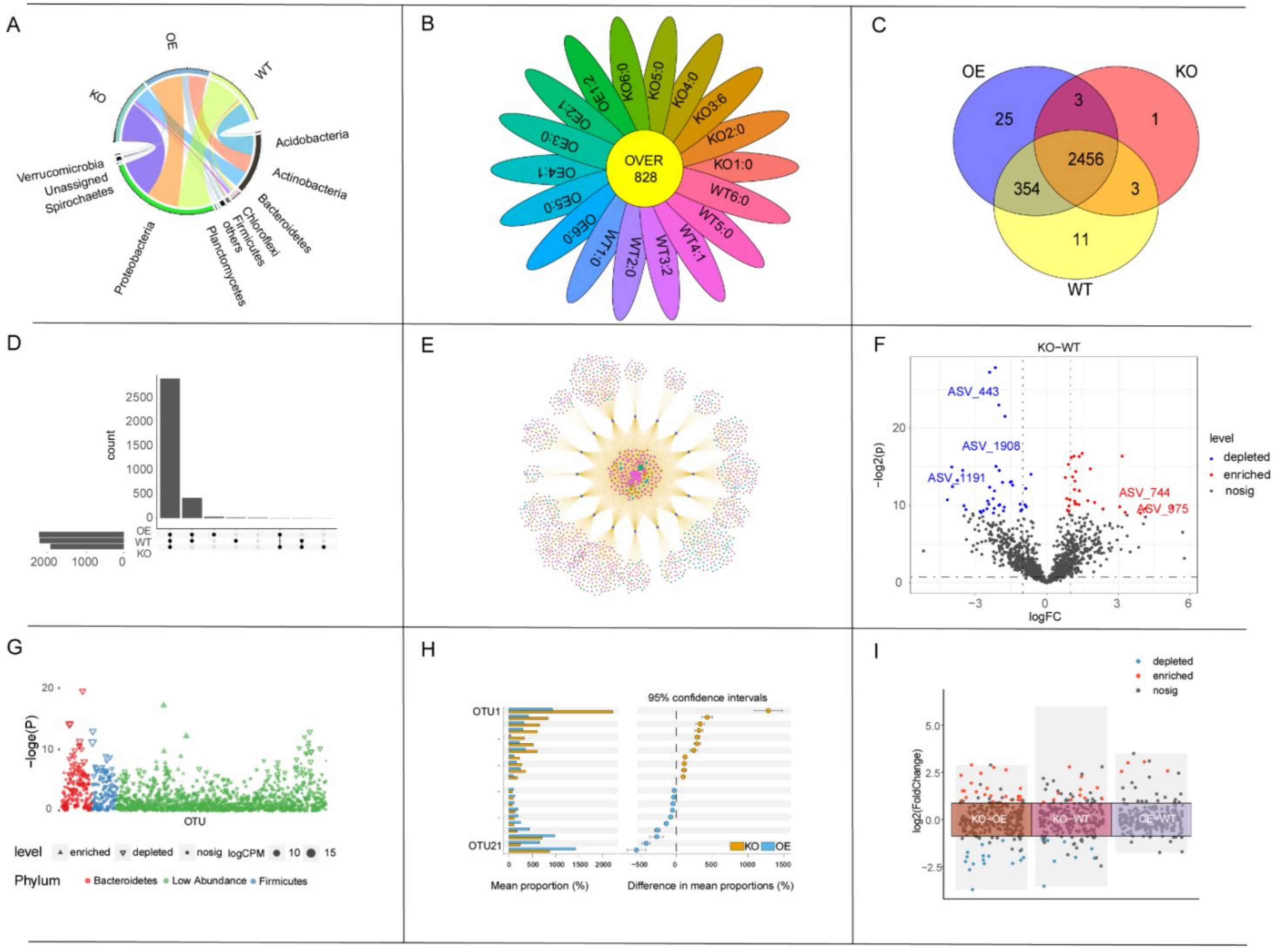

Fig. S11 Partial display of results from best practices in microbiome data analysis. (A-B) difference analysis, (C-D) biomarker identification, (E-I) network analysis

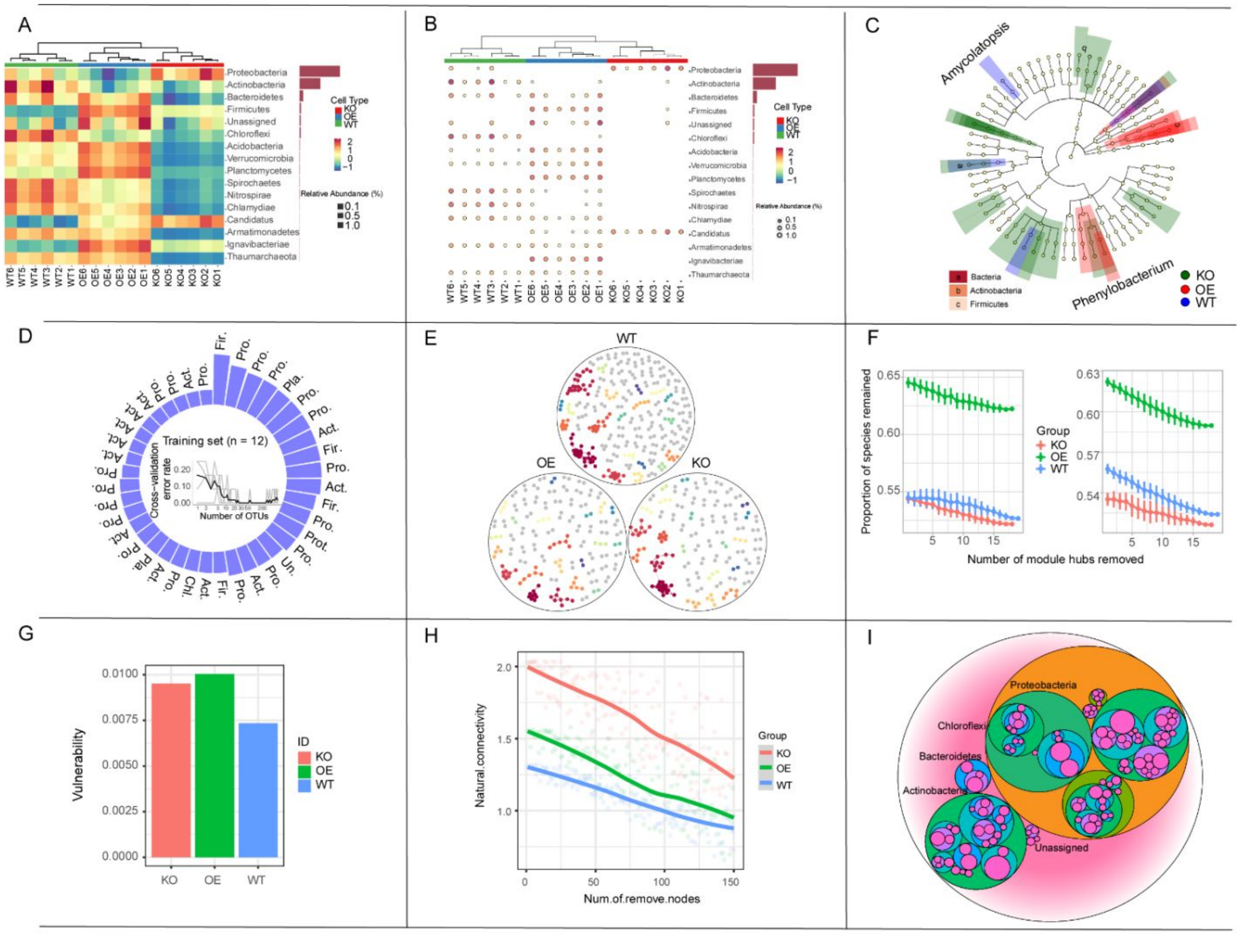

Fig. S12 Partial display of results from best practices in microbiome data analysis. (A-I) other microbiome analyses

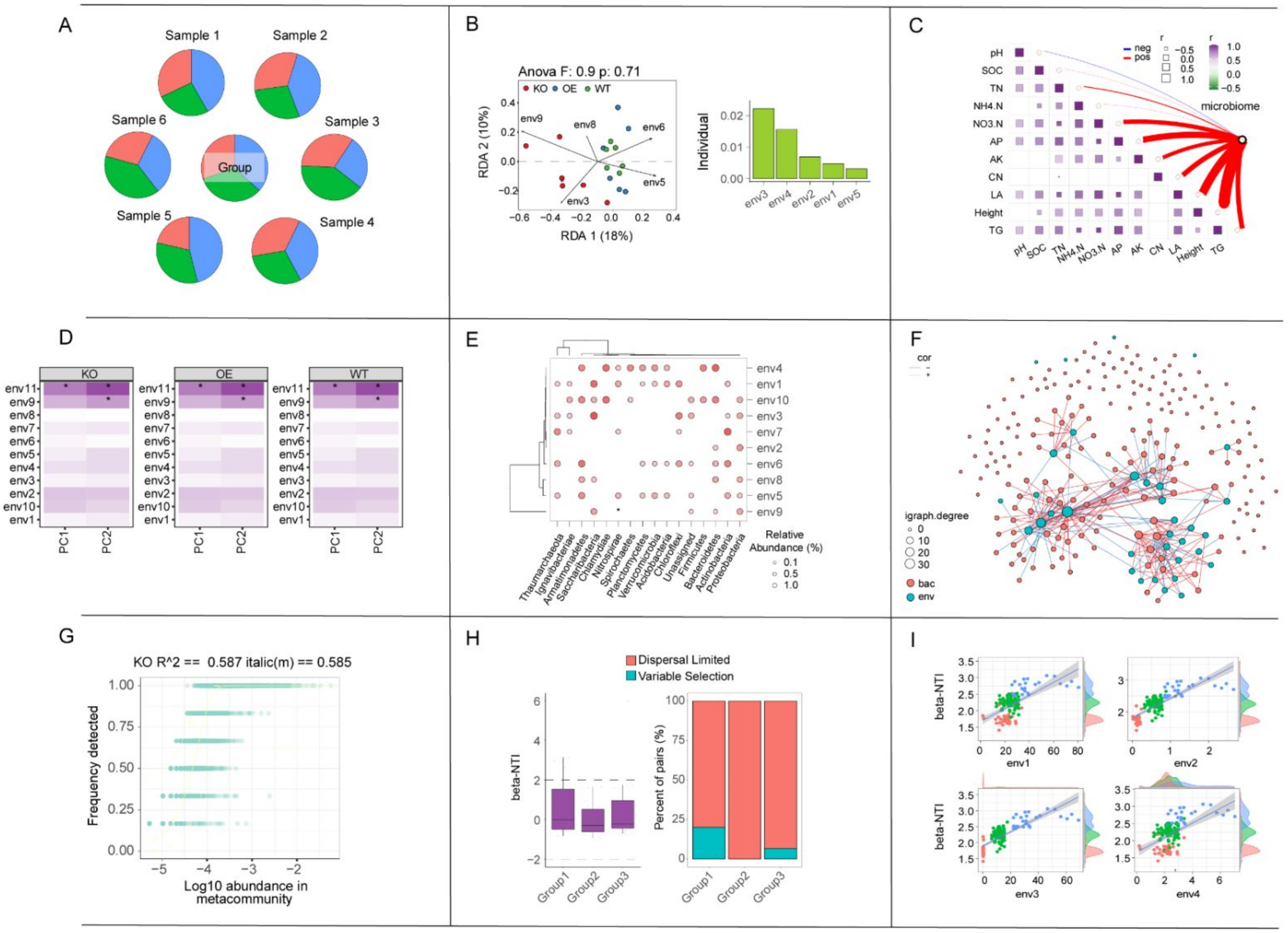

Sup. Table. Specific classification of 324 R packages in microbiome analysis.

Package	Classification
abind	Data cleaning
ade4	Diversity analysis
agricolae	Difference analysis
AlgDesign	Diversity analysis
annotate	Function prediction
AnnotationDbi	Function prediction
ape	Diversity analysis
aplot	Visualization
askpass	Dependented
assertthat	Dependented
backports	Dependented
base64enc	Data cleaning
bayesm	Biomarker identification
BH	Dependented
Biobase	Dependented
BiocGenerics	Dependented
BiocManager	Dependented
BiocParallel	Data cleaning
BiocVersion	Dependented
biomformat	Data cleaning
Biostrings	Dependented
bit	Diversity analysis
bit64	Data cleaning
bitops	Data cleaning
blob	Data cleaning
brew	Data cleaning
brio	Data cleaning
broom	Biomarker identification
bslib	Dependented
cachem	Dependented
callr	Dependented
car	Dependented
carData	Dependented
cellranger	Dependented
checkmate	Dependented
circlize	Visualization
classInt	Dependented
cli	Dependented
clipr	Dependented

For Peer Review

clue	Diversity analysis
coda	Data cleaning
coin	Difference analysis
colorspace	Visualization
combinat	Data cleaning
commonmark	Dependented
compositions	Diversity analysis
corrplot	Visualization
cowplot	Visualization
cpp11	Dependented
crayon	Visualization
credentials	Dependented
crosstalk	Visualization
curl	Dependented
data.table	Data cleaning
data.tree	Biomarker identification
DBI	Dependented
dbplyr	Data cleaning
DelayedArray	Dependented
deldir	Visualization
DEoptimR	Dependented
desc	Dependented
DESeq2	Difference analysis
devtools	Dependented
diffobj	Visualization
digest	Dependented
dirmult	Dependented
doParallel	Dependented
downlit	Dependented
dplyr	Data cleaning
dtplyr	Data cleaning
dynamicTreeCut	Diversity analysis
e1071	Biomarker identification
EasyStat	Difference analysis
edgeR	Difference analysis
ellipsis	Dependented
evaluate	Dependented
fansi	Dependented
farver	Visualization
fastcluster	Diversity analysis
fastmap	Dependented
fBasics	Diversity analysis
fontawesome	Visualization

forcats	Data cleaning
foreach	Data cleaning
formatR	Dependented
Formula	Dependented
fs	Dependented
fst	Dependented
fstcore	Dependented
futile.logger	Dependented
futile.options	Dependented
gargle	Dependented
genefilter	Difference analysis
geneplotter	Function prediction
generics	Dependented
GenomeInfoDb	Dependented
GenomeInfoDbData	Dependented
GenomicRanges	Function prediction
gert	Dependented
ggalluvial	Visualization
ggClusterNet	Network analysis
ggforce	Visualization
ggfun	Visualization
ggnewscale	Visualization
ggplot2	Visualization
ggplotify	Visualization
ggpubr	Visualization
ggraph	Visualization
ggrepel	Visualization
ggsci	Visualization
ggsignif	Visualization
ggstance	Visualization
ggstar	Visualization
ggtern	Visualization
ggtree	Visualization
ggtreeExtra	Visualization
ggupset	Visualization
ggVennDiagram	Visualization
gh	Dependented
gitcreds	Dependented
glmnet	Biomarker identification
GlobalOptions	Data cleaning
glue	Dependented
GO.db	Function prediction
googledrive	Dependented

For Peer Review

googlesheets4	Dependented
graphlayouts	Visualization
gridExtra	Visualization
gridGraphics	Visualization
gss	Dependented
gtable	Visualization
GUniFrac	Diversity analysis
haven	Dependented
hexbin	Visualization
highr	Dependented
Hmisc	Data cleaning
hms	Dependented
htmlTable	Dependented
htmltools	Dependented
htmlwidgets	Dependented
httpuv	Dependented
httr	Dependented
huge	Data cleaning
ids	Dependented
igraph	Network analysis
impute	Data cleaning
ini	Dependented
interp	Dependented
IRanges	Function prediction
isoband	Dependented
iterators	Dependented
jpeg	Visualization
jquerylib	Dependented
jsonlite	Dependented
KEGGREST	Function prediction
klaR	Biomarker identification
knitr	Dependented
labeling	Dependented
labelled	Dependented
lambda.r	Dependented
later	Dependented
latex2exp	Dependented
latticeExtra	Visualization
lazyeval	Dependented
libcoin	Dependented
lifecycle	Dependented
limma	Difference analysis
lme4	Biomarker identification

For Peer Review

locfit	Dependented
lubridate	Dependented
magrittr	Dependented
MatrixGenerics	Biomarker identification
MatrixModels	Biomarker identification
matrixStats	Dependented
memoise	Dependented
curatedMetagenomicData	Dependented
MicrobiotaProcess	Biomarker identification
mime	Dependented
miniUI	Dependented
minqa	Dependented
mnormt	Diversity analysis
modeest	Biomarker identification
modelr	Biomarker identification
modeltools	Biomarker identification
multcomp	Difference analysis
multcompView	Difference analysis
multtest	Difference analysis
munsell	Visualization
mvtnorm	Difference analysis
network	Network analysis
networkD3	Network analysis
nloptr	Dependented
numDeriv	Difference analysis
openssl	Dependented
packcircles	Network analysis
patchwork	Visualization
pbkrtest	Biomarker identification
permute	Difference analysis
pheatmap	Difference analysis
picante	Biomarker identification
pillar	Dependented
pixmap	Visualization
pkgbuild	Dependented
pkgconfig	Dependented
pkgdown	Dependented
pkgload	Dependented
plogr	Dependented
plotly	Visualization
plyr	Data cleaning
png	Visualization
polyclip	Dependented

For Peer Review

polynom	Dependented
praise	Dependented
preprocessCore	Dependented
prettyunits	Dependented
processx	Dependented
profvis	Visualization
progress	Dependented
promises	Dependented
proto	Dependented
proxy	Dependented
ps	Visualization
psych	Network analysis
pulsar	Visualization
purrr	Data cleaning
quantreg	Difference analysis
questionr	Data cleaning
R.cache	Dependented
R.methodsS3	Dependented
R.oo	Dependented
R.utils	Dependented
R6	Dependented
ragg	Visualization
randomForest	Biomarker identification
SIAMCAT	Biomarker identification
rappdirs	Dependented
rcmdcheck	Dependented
RColorBrewer	Visualization
Rcpp	Dependented
RcppArmadillo	Dependented
RcppEigen	Dependented
RCurl	Dependented
readr	Data cleaning
readxl	Data cleaning
rematch	Dependented
rematch2	Dependented
remotes	Dependented
reprex	Dependented
reshape2	Data cleaning
rgexf	Visualization
rhdf5	Dependented
rhdf5filters	Dependented
Rhdf5lib	Dependented
rlang	Dependented

For Peer Review

rmarkdown	Dependented
rmutil	Biomarker identification
robustbase	Biomarker identification
roxygen2	Dependented
rprojroot	Dependented
RSQLite	Dependented
rstatix	Difference analysis
rstudioapi	Dependented
RVenn	Visualization
rversions	Dependented
rvest	Dependented
s2	Dependented
S4Vectors	Dependented
sandwich	Difference analysis
sass	Dependented
scales	Visualization
selectr	Dependented
servr	Dependented
sessioninfo	Dependented
sf	Dependented
shape	Visualization
shiny	Dependented
sna	Network analysis
snow	Network analysis
sourcetools	Dependented
sp	Visualization
SparseM	Dependented
SpiecEasi	Network analysis
stable	Difference analysis
stabledist	Difference analysis
statip	Difference analysis
statmod	Biomarker identification
statnet.common	Dependented
stringi	Data cleaning
stringr	Data cleaning
styler	Dependented
SummarizedExperiment	Data cleaning
sys	Dependented
systemfonts	Dependented
Tax4Fun2	Function prediction
tensorA	Dependented
testthat	Dependented
textshaping	Visualization

For Peer Review

TH.data	Dependented
tibble	Dependented
tidyfst	Data cleaning
tidygraph	Visualization
tidyr	Data cleaning
tidyselect	Dependented
tidytree	Diversity analysis
tidyverse	Data cleaning
timechange	Dependented
timeDate	Dependented
timeSeries	Dependented
tinytex	Dependented
treeio	Diversity analysis
tweenr	Visualization
tzdb	Dependented
units	Dependented
urlchecker	Dependented
vegan	Diversity analysis
VGAM	Difference analysis
vroom	Data cleaning
waldo	Difference analysis
WGCNA	Network analysis
adespatial	Diversity analysis
minpack.lm	Other analysis
eulerr	Other analysis
FSA	Other analysis
stats4	Other analysis

For Peer Review

Running title: Using R language in microbiome analysis

REVIEW

The best practice for microbiome analysis using R

Tao Wen[1,2, ‡], Guoqing Niu[2, ‡], Tong Chen[3], Qirong Shen[2], Jun Yuan[2,*], Yong-Xin Liu[1,*]

[1] Shenzhen Branch, Guangdong Laboratory of Lingnan Modern Agriculture, Genome Analysis Laboratory of the Ministry of Agriculture and Rural Affairs, Agricultural Genomics Institute at Shenzhen, Chinese Academy of Agricultural Sciences, Shenzhen 518120, China

[2] The Key Laboratory of Plant Immunity Jiangsu Provincial Key Lab for Organic Solid Waste Utilization Jiangsu Collaborative Innovation Center for Solid Organic Waste Resource Utilization, National Engineering Research Center for Organic-based Fertilizers, Nanjing Agricultural University, Nanjing 210095, China

[3] National Resource Center for Chinese Materia Medica, China Academy of Chinese Medical Sciences, Beijing 100700, China

[‡]These authors contributed equally to this work

[*]Correspondence: junyuan@njau.edu.cn (J. Yuan), liuyongxin@caas.cn (Y.-X. Liu)

ORCIDs

Tao Wen: https://orcid.org/0000-0003-0102-0487
Guoqing Niu: https://orcid.org/0009-0007-4100-8470
Tong Chen: https://orcid.org/0000-0003-3134-3113
Qirong Shen: https://orcid.org/0000-0002-5662-9620
Jun Yuan: https://orcid.org/0000-0002-8265-0239
Yong-Xin Liu: https://orcid.org/0000-0003-1832-9835

For Peer Review

Abstract

With the gradual maturity of sequencing technology, many microbiome studies have published, driving the emergence and advance of related analysis tools. R language is the widely used platform for microbiome data analysis for powerful functions. However, tens of thousands of R packages and numerous similar analysis tools have brought major challenges for many researchers to explore microbiome data. How to choose suitable, efficient, convenient, and easy-to-learn tools from the numerous R packages has become a problem for many microbiome researchers. We have organized 324 common R packages for microbiome analysis and classified them according to application categories (diversity, difference, biomarker, correlation and network, functional prediction, and others), which could help researchers quickly find relevant R packages for microbiome analysis. Furthermore, we systematically sorted

the integrated R packages (phyloseq, microbiome, MicrobiomeAnalystR, Animalcules, microeco, and amplicon) for microbiome analysis, and summarized the advantages and limitations, which will help researchers choose the appropriate tools. Finally, we thoroughly reviewed the R packages for microbiome analysis, summarized most of the common analysis content in the microbiome, and formed the most suitable pipeline for microbiome analysis. This paper is accompanied by hundreds of examples with 10,000 lines codes in GitHub, which can help beginners to learn, also help analysts compare and test different tools. This paper systematically sorts the application of R in microbiome, providing an important theoretical basis and practical reference for the development of better microbiome tools in the future. All the code is available at GitHub.

Keywords R package, microbiome, data analysis, visualization, amplicon, metagenome

Introduction

For Peer Review

The metagenomic analysis is used to study microbial diversity, structure, and function by sequencing, quantifying, annotating, and analyzing DNA and/or RNA sequences of microbial communities or microbiota. The commonly used high-throughput sequencing technology in microbiome research is mainly known as amplicon sequencing and shotgun metagenomic sequencing. Amplicon sequencing with the advantages of low cost, mature analysis system, and simple analysis process was widely used in microbiome research. Shotgun metagenomic sequencing provided the functional information of microbes and more accurate information on the microbial composition with the higher sequencing cost and large amount of computational resources needed. The detail pipeline for both sequencing have been systemically summarized in our previous review (Liu et al., 2021). As an important component of biodiversity, microbial communities play a vital role in biology, ecology, biotechnology, agriculture, and medicine. Various bioinformatics methods are required for microbial community analysis, which mainly includes three parts: 1) data preprocessing, 2) quantification and annotation, and 3) statistics and visualization (Fig. 1A). In the preprocessing step, the raw data is filtered and quality controlled to ensure data quality. In the quantification and annotation step, tools and databases are used to identify microbial representative sequences and annotate microbial taxonomy and function. The first two parts of microbial community analysis have been well discussed and could be well done according to our previous papers (Liu et al., 2023). Finally, in the statistics and visualization step, various statistical methods are used to explore microbial community diversity, structure, and potential functions.

With the development of high-throughput sequencing technology, plenty of studies were performed with amplicon-sequencing technology (Thompson et al., 2017; Proctor et al., 2019) and shotgun metagenomes sequencing (Carrión et al., 2019; Li et al., 2022; Paoli et al., 2022), which led to the development of microbiome analysis methodologies, software, and pipelines, e.g., QIIME (Caporaso et al., 2010), Mothur (Schloss et al., 2009), USEARCH (Edgar, 2010), VSEARCH (Rognes et al., 2016), QIIME 2 (Bolyen et al., 2019) , Parallel-Meta Suite (Chen et al., 2022),

EasyAmplicon (Liu et al., 2023), Kraken (Wood and Salzberg, 2014), MEGAN (Huson et al., 2007), MetaPhlAn2 (Truong et al., 2015), HUMAnN2 (Franzosa et al., 2018) etc. As the most crucial and basic procedure for amplicon sequencing data analysis, OTU (Operational taxonomic unit) clustering method was popular before the year of 2015 while non-clustering methods were gradually developed and widely used recently. Currently, the common non-clustering methods include DADA2 (Callahan et al., 2016), deblur (Amir et al., 2017), unoise3 (Edgar and Flyvbjerg, 2015). One of the most representative non-clustering algorithms among them is DADA2, which was created with R language. It makes the R language (Ihaka and Gentleman, 1996) occupy an important position in raw data processing for amplicon sequencing. Compared with many software that can be used in upstream steps of microbiota sequencing data analysis, the downstream analysis steps rely on the R language heavily with various packages. These analyses mainly include: 1) Diversity analysis; 2) Difference analysis; 3) Correlation and network analysis; 4) Biomarker identification; 5) Functional predictions; 6) Integrative analysis of microbial communities with other indicators (including phylogenetic analysis, multi-omics integration, and environmental factor analysis, etc.). In addition to the kinds of multivariate statistical analysis that can be done in R, there are diversified data-cleaning packages that allow data to be transformed among different analyses.

R is a free, open-source language and environment for data statistical analysis and visualization, which was created by Ross Ihaka and Robert Gentleman from the University of Auckland in New Zealand and now is responsible by the "R Development Core Team". Compared with other analysis tools, such as SPSS, MINITAB, MATLAB, which are more suitable for the statistics of processed and standardized data, R language can handle processed data as well as raw data. R can easily implement almost all analysis methods, many of the latest methods or algorithms were first exhibited in it. Furthermore, R shows excellent data visualization, particularly for complex data. The powerful and flexible interactive analysis is also an advantage of R, meanwhile enabling visual data exploration. The functionality of the R language relies heavily on thousands of R packages, which provide a wide variety of data processing and analysis strategies, allowing almost any data analysis process to be done in R. The total number of R packages published on CRAN is 18,981, and Bioconductor is 2,183 (by January 31, 2023). These packages demonstrated the powerful data process and analysis performance of R.

In recent years, numerous R packages have been developed on the R platform for the downstream analysis of microbiome, which have made important contributions to the associated-research field. However, the increasing number of downstream analysis R packages has reached a dizzying level (Fig. 1B). In addition, integrated R packages containing a large amount of microbiome analysis content, such as phyloseq (McMurdie and Holmes, 2013), microeco (Liu et al., 2020), and amplicon (Liu et al., 2023), have gradually emerged. This abundance of R packages provides microbiome analysts with more choices, but also makes it difficult to identify the most suitable tools among many similar analysis tools. Furthermore, this plethora of R packages make it difficult for beginners to embark on a well-organized learning path for

For Peer Review

microbiome analysis. Therefore, it is urgent to compare similar analysis functions, and extract the similarities and differences functions, to select the best process for microbiome analysis and help beginners learn more effectively.

This paper attempts to sort and run the 324 common R packages (Fig. S1), especially the integrated R packages for microbiome analysis, and complete the following three parts: 1) compare different R package analysis processes according to the functional categories of microbiome analysis, analyze the results, and summarize example code; 2) organize the content of six integrated R packages according to the functional categories of microbiome analysis, compare the analysis results, and generate example code; 3) based on all R packages, select the optimal analysis approach using R language and provide example code for reference and learning to researchers.

Preparing microbiome data analysis

Downstream analysis of microbiome requires the preparation of five data files, including a feature table, a feature annotation file, a sample metadata file, a phylogenetic tree, and representative sequences. For beginners, it is important to understand the format and basic data structure of these files and learn how to import these files into R language. Furthermore, different analytical contents often have different requirements for data, and it is necessary to learn some data manipulation skills to meet the demands of various functions. Finally, it is necessary to learn the basics of R plotting to facilitate the presentation of results.

Data preparation and cleaning

After the process of sequence data preprocessing, quantification, and annotation, we need to further analysis the output files, including importing these files, cleaning data, and converting format, which required for subsequent microbiome analysis in R. Before statistical analysis, we must master the basic procedure of R language to cope with the data input requirements of different packages. This section includes: importing, organizing, filtering, basic calculations, conversion, normalization, and modification of data. Five data forms are frequently used from raw data processing, including feature tables (file formats are .csv/.txt/.xlsx/.biom, typically used taxonomic and functional tables, including OTU/ASV/taxonomy/gene/module/pathway tables), feature annotation (.csv/.txt/.xlsx/.biom), sample metadata (.csv/.txt), evolutionary/phylogenetic trees (.nwk/.tree), representative sequences (.fasta/.fas/.fa). All the data cleaning-related packages show in Fig. 1C. Tabular data input for microbial community is primarily accomplished using functions such as *read.table*(), *read.delim*(), and *read.csv*() in the utils package (Code 1A, script in GitHub). The reading of evolutionary tree files depends on functions like *read.tree*() in the ape/ggtree/treeio package, or *read_tree*() in the phyloseq package. For reading representative sequence files in microbiome, the *readDNAStringSet*() in the Biostrings package (Pages et al., 2016) is typically used. Currently, big data integration of microbiome has become a trend, and leading to the

For Peer Review

emergence of R packages for integrated data from multiple studies, likes curatedMetagenomicData (Pasolli et al., 2017). The package only needs to import the package and could re-analysis the curated data, rather than input in raw sequencing data.

The basic idea of data organization can be summarized as three steps: splitting the data, processing with functions, and combining the output results into the desired format. The functions of basic packages in R can be combined to meet most requirements of the microbiome data operations. For example, the "for loop" combined with the basic statistical functions [*sum*(), *mean*(), *sd*(), etc.] can be used to perform basic statistical analysis and data transformations for microbial relative abundance (Code 1B); the base package provides the apply family of functions, including *apply*(), *sapply*(), *lapply*(), *tapply*(), *aggregate*(), etc., which can be applied to quickly complete the three stages of data processing. The apply family of functions provides a framework that acts as an alternative to "for loop" and is much faster than the basic "for loop" function in R (Code 1B). A similar purr package can be used in place of "for loop" to perform efficient operations.

The plyr (Wickham, 2011b) package was upgraded from package of base with a variety of data sorting processes for kinds of data frames, lists, etc. The plyr package provides three data processing stages "Split–Apply–Combine" in one function, and the plyr package implements grouping transformations between R types (vector, list, and data frame) and basically replaces the apply family of functions in the base package. It can easily handle grouping calculations, e.g., microbial abundance at different taxonomy levels (Code 1C). The reshape2 (Wickham, 2007) package provides the long-wide format transformation during data processing, and since ggplot2 (Wickham, 2011a) plotting functions and most modeling functions, such as *lm*(), *glm*(), *gam*(), often use long data, microbiome data are general showed as wide form, so the transformation of microbiome data for plotting can be done using reshape2 (Code 1D), which provides the long-wide format transformation during data processing.

The dplyr package is a member of the tidyverse family, innovatively abandoning the common form of data preservation in R rather than using the tibble format (more powerful than data.frame format) for data processing, which can more efficiently complete the data frame selection, merging and statistics within row and column, and data frame length and width format changes, the "%>%" pipeline symbol can be used to complete more complex data processing. The tibble format can store data during the analysis and modeling process, which is important for data analysis. For example, we demonstrated the use of dplyr and pipeline to run random forest modeling and the selection process of important variables (Code 1E).

Visualization in R language

In most cases, we are used to plotting standard graphs in microbiome data display such as alpha/beta diversity, taxonomic composition. All the visualization-related packages show in Fig. 1C. Due to the widespread use of ggplot2 (Code 2A), many

For Peer Review

extension packages have emerged to extend based on ggplot2 with a high capacity of plotting styles, colors, and themes. These packages mainly include ggtern plotting ternary graphs in Code 2B (Hamilton and Ferry, 2018), ggraph plotting network graphs in Code 2C (Si et al., 2022), ggtree plotting evolutionary tree or cladogram in Code 2D (Xu et al., 2022), the ggalluvial package, the ggVennDiagram package (Code 2E), the ggstatsplot package plotting pie chart, and the ggpubr package providing many various themes and colors of output. In addition, the pheatmap and ComplexHeatmap package (Gu, 2022) based on the grid mapping system plots the relative abundance of features in different samples (Code 2F), the VennDiagram package (Chen and Boutros, 2011) could show the number of features in different samples. The UpSetR package (Conway et al., 2017), which draws Upset view is a new form plotting similar to Venn diagram. The base-based plotting system is complex and difficult to learn, while it is a good choice for complex graph drawing, such as the circlize (Gu et al., 2014) package (Code 2G), which draws chord diagrams composed of microbiota.

Additionally, there is often a lot of microbiome mapping work that involves a combination of graphics. At present, many tools in R can combine graphics, such as cowplot, patchwork, and aplot. The patchwork package has the most powerful functions and supports modular splicing graphics (Code 2H).

Microbial community analysis

We have categorized the analysis of microbiome data into the following six major types in Fig. 1D: diversity analysis, difference analysis, biomarkers identification, correlation and network analysis, functional prediction, and other microbiome analyses (including source tracking analysis, community assembly processes, and analysis of associations between microbiota and environmental factors). Then, we would have organized, compared, and summarized all relevant R packages.

Diversity analysis

Microbial community diversity mainly includes alpha diversity (Richness, Shannon, Simpson, Chao1, ACE, etc.), rarefaction curve, beta diversity (ordination and clustering analysis), taxonomic or functional composition. Here must introduce the package vegan (Oksanen et al., 2007), an abbreviation for Vegetation Analysis, written by nine quantitative ecologists, including Oksanen from Finland, which is initially used for specifical dealing with data on community ecology. The package provides a variety of methods for data standardization and transformation. For example, data used for alpha diversity analysis can be normalized at the same sequencing depth with *rrarefy*(), and data for ordination analysis can be normalized with the *decostant*() (Code 3A). After the sequencing data are sampling normalization, diversity calculation can be more reasonable. In addition, alpha diversity metrics calculation can also be carried out with the ade4 (Dray and Dufour, 2007), adespatial (Dray et al., 2018), and picante packages (Kembel et al., 2010). For example, phylogenetic diversity can be calculated using the *pd*() in the picante

For Peer Review

package (Code 3A). Vegan not only allows for alpha diversity analysis, but also provides functions such as *rda*() for conducting principal components analysis (PCA) and redundancy analysis (RDA), *cca*() for conducting correspondence analysis (CA) and canonical correspondence analysis (CCA), *decorana*() for conducting decision curve analysis (DCA), and *metaMDS*() for conducting non-metric multidimensional scaling (NMDS) for microbiome ordination analysis (Code 3B). The *prcom*() in stats package can be used for principal component analysis (PCA), which is a kind of dimension reduction analysis. The *mca*() provided by the MASS package and the *MCA*() provided by the FactoMineR package can be used for multiple correspondence analysis (Code 3B); the ape package provides the *pcoa*() function for principal coordinate analysis (PCoA); the MASS package provides *lda*() for linear discriminant analysis (LDA, Code 3C). Before running many ordination operations, it is often necessary for community clustering. The *vegdist*() in the vegan package can calculate euclidean, manhattan, bray, canberra, and other distances (Code 3B). In addition, distance calculation can also be done using *dist*() of stats package. The distance matrix can be used for clustering analysis in addition to ordination analysis. The *hclust*() in the stats package can be used for clustering analysis, a similar function can be achieved with the facteoextra, kmeans packages (Code 3D). Microbial composition analysis mainly used to display the abundance of microbes, and the dplyr package is needed to organize the data then display with ggplot2 subsequently.

Difference analysis

Difference analysis is divided into community-level analysis and feature-level (any hierarchy of taxonomy and function) analysis. Community-level difference analysis is mainly performed with functions including *adonis*(), *anosim*(), and *mrpp*() in vegan package, and *mantel.test*() in ape package (Code 4A). The R package for compositional data difference analysis in the feature level can utilize the *wilcox.test*() (Code 4B) and *t.test*() (Code 4C) in the stats package. Subsequently, data correction algorithms were developed specifically for sequencing data, such as the upper quartile (UQ), trimmed mean of M-values (TMM) (Code 4C), and relative log expression (RLE) harbored in the edgeR package (Robinson et al., 2009) (Code 4D). Median of ratios method (MED) in DESeq2 package (Love et al., 2014) (Code 4E), and cumulative-sum scaling (CSS) algorithm in metagenomeSeq ~~(https://github.com/sirusb/metagenomeSeq)~~ package (Code 4F). Furthermore, the ALDEx2 package provides polynomial models which can be used to infer feature abundance and calculate feature differences with non-parametric tests, t-tests, or generalized linear models (Code 4G). The ANCOM-BC package attempts to address sample heterogeneity by correcting bias with a log-linear model. In addition, other R packages for microbiome data correction and difference tests include limma (Code 4H), DR, ANCOM (Lin and Peddada, 2020) (Code 4I), corncob (Code 4J), Maaslin2 (Code 4K), etc. Nearing et al. (2022) showed that they compared these difference analysis methods and proposed that ALDEx2 and ANCOM-II (anchom_v2.1.R, Code 4L) were the best performers in the difference analysis of microbial communities. As for the significance test, different packages use different methods for significance

testing. For example, Fisher test was used in edgeR package; Wald test was used in DESeq2 and corncob package; t-test was used in limma package. There was other method for significance test, likes Wilcoxon rank-sum test (ALDEx2 and ANCOM-II), ANOVA (Maaslin2) etc.

Biomarker identification

Characteristic microbial consortia were explored to explain certain questions, such as the biomarkers of the gut in obese or hypertensive populations, or of soil in Fusarium wilt develops, etc. Microbes selected through difference analysis are often unable to determine whether they represent the main differences of concern. Therefore, weight analysis or machine learning methods are used to further distinguish the feature microbes.

The main ones commonly used for weighted analysis are linear discriminant analysis effect size (LEfSe), PCA, etc (Code 5A). LEfSe is developed specifically for microbiome data, and the core functionality is implemented using the packages LDA (Fisher, 1936) and MASS (Ripley et al., 2013). By extracting the loading matrix of PCA ordination, the microbiome with the greatest impact on the sample variation are found as biomarkers (Code 5B).

In terms of machine learning, the random forest model, which is widely used in microbiome analysis, is implemented by using the randomforest package (Liaw and Wiener, 2002) (Code 5C). There are many other decision tree-based machine learning models, such as the mboost (Hofner et al., 2014) package provides boosting-based algorithms, the e1071 (Dimitriadou et al., 2008) package provides support vector machines *svm*() in Code 5D, and plain Bayes *naiveBayes*(). The xgboost package can integrate many tree models together to form a strong classifier, which can prevent overfitting via many strategies, including regularization terms, shrinkage, and column subsampling, etc. In addition, the pROC (Robin et al., 2011) package is used to plot the operating characteristic curve (ROC, Code 5D) to evaluate the efficiency of machine learning models. The Caret package provides cross-validation to determine the number of features (Kuhn, 2008). Currently, Jakob et al (2021) developed an open-source R package SIAMCAT, a powerful yet user-friendly computational machine learning toolkit tailored to the characteristics of microbiome data.

Correlation and network analysis

Microbial co-occurrence network analysis is used to find microbial modules that may have mutualistic relationships. Co-occurrence network analysis mainly includes the calculation of correlations, network visualization, and the calculation of network properties. The common R packages for calculation of correlations are psych (Revelle and Revelle, 2015) (Code 6A), WGCNA (Langfelder and Horvath, 2008) (Code 6B), Hmisc (Harrell Jr and Harrell Jr, 2019) (Code 6C), and SpiecEasi (Kurtz et al., 2015) (Code 6D). Among these R packages, WGCNA has the highest calculation speed, while requiring additional p-value correction; psych can calculate correlation with correct p-value, but the speed is very low; the SpiecEasi package can use the sparcc method to perform a more suitable method for microbiome data to calculate the

For Peer Review

correlation matrix, and can call multiple-threads to accelerate the calculation. R packages for network visualization and attribute calculation can use igraph (Code 6E), network, and ggraph packages (Code 6F). These R packages contain many layout algorithms for network visualization. In addition, network packages combined with ggplot2 to visualize the network are easier to modify. Sna and ggraph packages have many visualization layout algorithms to increase the styles of network visualization. With the increasing use of network analysis in the microbiome analysis, more attention is paid to network modularity and the key groups through network modules. The WGCNA package provides a complete framework to quickly complete the correlation calculation, network module calculation, module feature vector calculation, and other network properties exploration. The recent development of the ggClusterNet (Wen et al., 2022) package (Code 6G) provides a unified framework for microbiome networks and designs a variety of unique module-based visualization algorithms to visualize the module relationships in the network.

Functional prediction

The Tax4Fun (Aßhauer et al., 2015) R package (Code 7A) for functional prediction of 16S rDNA has been developed to more accurately predict changes in microbial community function using amplicon data. The package has been updated to Tax4Fun2 (Wemheuer et al., 2020). Microeco can implement FAPROTAX (Louca et al., 2016) prediction for bacteria/archaea and FUNGuild (Nguyen et al., 2016) prediction for fungi, which is based on the database of taxonomic functional description from curated published papers. Functional prediction enables the prediction of microbial community function and subsequent statistical analysis. Additionally, vegan can be used for diversity analysis, while edgeR, DEseq2, and limma packages can be used for difference analysis. For functional enrichment, the clusterProfiler (Code 7B) package can perform GO, KEGG, GSEA and GSVA enrichment, which considers gene/pathway abundance and is recommended. Furthermore, the clusterProfiler package provides plot functions based on the ggplot syntax, allowing to plot appealing graphics in a simple manner. Gene/~~pathway~~ Pathway network analysis can be performed using WGCNA for calculation, and ggClusterNet for network parameter calculation and visualization. However, the reliability of functional prediction results, particularly for environmental samples, is currently disputed , and therefore, further verification of analysis results is often required.

Other microbiome analysis

Analysis for microbial community formation process commonly used in the framework proposed by Stegen et al. (2013) to calculate βNTI and RC-Bray indices with R packages minpack.lm, picante, Hmisc, eulerr, FSA, ape, stats4, and others (Code 8A). Ning et al. (2020) used a phylogenetic binning-based null model analysis to infer quantitative mechanisms underlying community assembly, and developed the R package iCAMP (Code 8B). It allows for the quantitative assessment of the relative importance of different ecological processes (e.g., homogenizing selection, heterogenizing selection, dispersal, and drift) on both the entire community and each

For Peer Review

phylogenetic bin (which is usually composed of taxa from a single family or order with distinct ecological characteristics). In addition, the package also provides neutral theory models, phylogenetic and taxonomic null model analyses at both the community and clade levels, calculation of niche differences and phylogenetic distances between clades, and tests for phylogenetic signals within individual phylogenetic bins.

Microbial communities were often used to analyze the correlation with environment indicators, for example, *mantel.test*() provided by the vegan package was used to examine the correlation between microbial communities and environment indicators, and using *wascores*(), *mantel.correlog*() to detect the phylogenetic signal between microbial communities and environmental factors (Code 8C). In addition, the ggClusterNet package can be used to calculate the co-occurrence relationships between microbes/microbiome and environmental factors, and generated publish-ready figures (Code 8D).

Knights et al. (2011) proposed the microbiome traceability tool source tracker in R language. Metcalf et al. (2016) predicted the time of death and tracked the source microbes of real cadavers on microbial communities, then microbial traceability analysis gradually popular. Shenhav et al. (2019) proposed a new algorithm in R, FEAST (Code 8E), which makes microbial traceability analysis more efficient, faster, and with low false positives.

Integrated R packages for microbiome

As microbiome sequencing becomes more popular, R packages dedicated to microbiome data processing are gradually emerging (Fig. 2). McMurdie and Holmes (2013) developed the phyloseq package: a comprehensive tool for microbiome data (including feature tables, phylogenetic trees, and feature annotation) clustering, integrating data import, storage, analysis, and output. The package utilizes many tools in R for ecological and phylogenetic analyses (vegan, ade4, ape, and picante) and uses ggplot2 to output high-standard figures. The data storage structure uses a S4-like storage system to store all relevant data as a single experiment-level object, thus making it easier to share data and reproduce the analysis. Subsequently, the packages microbiome~~ (https://github.com/microbiome/microbiome)~~, the MicrobiomeAnalystR (Chong et al., 2020), microViz (Barnett et al., 2021), and microbiomeSeq emerged under this framework. Subsequently, the microeco package according to the S6 framework, which provides more analysis functions. With the need for data interactive analysis, Animalcules (Zhao et al., 2021) emerged. EasyMicroPlot ~~(https://github.com/xielab2017/EasyMicroPlot) ~~also uses an interactive interface for microbiome data exploration, allowing for rapid downstream analysis of the microbiome (Fig. 3; Table 1).

Microbiome data analysis using phyloseq

Phyloseq, using the S4 class object, is more suitable for object-oriented programming and has had a great impact on microbiome data analysis (Figs. 2~~/~~, 3~~, Fig. ~~ and S2A–

For Peer Review

G, Pipeline 1. phyloseq.Rmd). Through the S4 class object, phyloseq allows the five parts of data (the feature table, feature annotation, metadata, representative sequences, and evolutionary tree) to maintain correspondence under the same framework, and provides a variety of multiple filtering functions on microbial features and samples, allowing the five parts of data to be filtered consistently without considering different among data. It also provides microbiome analysis through microbial data filtering and normalization, diversity calculation (Fig. S2A~~-~~ and S2B), microbial composition visualization (Fig. S2C~~-~~ and S2D), evolutionary tree visualization, and network analysis (Fig. S2E). The beta diversity function provides more than 30 distance algorithms, far more than those provided by packages such as vegan. Secondly, the phyloseq package uses ggplot for graphical visualization (Fig. S2F), which is easier to generate and modify figures. Additionally, phyloseq can integrate the evolutionary tree and feature taxonomic and abundance on tree branches and leaves (Fig. S2G), which makes the tree informative and beautiful.

Microbiome data analysis using microbiome

The microbiome package also uses S4 class objects, like **phyloseq**, and can also perform most of the analysis of microbiomes (Figs. 2~~/~~, 3~~, Fig.~~ and S3A~~-~~–G, Pipeline 2. Microbiome.Rmd). It includes microbial diversity analysis (Fig. S3A~~-~~–E), and difference analysis (Fig. S3F~~-~~ and S3G). Compared with phyloseq, the microbiome package is richer in alpha diversity indicators, which provides more than 30 alpha diversity indicators. Secondly, it provides core microbial calculation and visualization functions. In general, it can be used as a complement to phyloseq or in conjunction with it.

Microbiome data analysis using MicrobiomeAnalystR

MicrobiomeAnalystR is an R package version according to the MicrobiomeAnalyst webserver (Figs. 2~~/~~, 3~~, Fig.~~ and S4A~~-~~–J, Pipeline 3. MicrobiomeAnalystR.Rmd). These functions include diversity analysis (Fig. S4A~~-~~–F), difference analysis (Fig. S4G), biomarker identification (Fig. S4H~~-~~ and S4I), sample sequencing library size overview (Fig. S4J), which are more powerful than the previous two packages. The visualization combines basic packages, ggplot plotting, and interactive plotting. In terms of network analysis, it provides the process of calculating and plotting SparCC networks that are more suitable for microbiome data. However, the package depends on many R packages from CRAN, Bioconductor, and GitHub, so a complete installation of MicrobiomeAnalystR requires a lot of effort.

Microbiome data analysis using Animalcules

The **Animalcules** package is an alternative way to analysis in an interactive platform (Figs. 2~~/~~, 3~~, Fig.~~ and S5A~~-~~–J, Pipeline 4. Animalcules.Rmd). It is possible to

For Peer Review

calculate and plot sample statistics in bar plot (Fig. S5A) or interactive pie charts (Fig. S5B), calculate, and visualize alpha diversity dot plot (Fig. S5C), group microbial taxonomic or functional composition heatmap and stack plot (Fig. S5D~~-~~ and S5E), feature abundance in boxplot (Fig. S5F), genus bray distance heatmap (Fig. S5G), ordination analysis (Fig. S5H~~-~~ and S5I), using randomforest, logistic regression to select biomarkers (Fig. S5J), and other analyses. The results of these analyses can often be reanalyzed by interactively modifying parameters, and the images can be interactively zoomed in and out, clicked to see details, and other operations performed by the mouse for better pattern discovery. However, the results cannot be exported as vector format, which do not meet the requirements for publication. Secondly, the analysis content is too little, especially the microbiome network analysis, the correlation analysis between the microbiome and other indicators.

Microbiome data analysis using microeco

The microeco package is very powerful, using R6 class data structure (Figs. 2~~/~~, 3~~, Fig.~~ and S6A~~-~~–L, Pipeline 5. microeco.Rmd). It includes microbial diversity (Fig. S6A/B) taxonomic composition (Fig. S6C~~-~~–E), difference (Fig. S6F~~-~~–H), biomarker (Fig. S6I~~-~~ and S6J), network (Fig. S6K), integrated community structure with environmental factor (Fig. S6L), and phylogenetic diversity analysis. It can complete almost all the current microbiome analysis contents. However, it is not suitable for novices because there is a certain threshold for using S6 class objects. In addition, due to too many functions, the requirements for input data are different, causing some functions are hard to use.

For Peer Review

Microbiome data analysis using amplicon

The package amplicon is an analysis and plotting tool (Figs. 2~~/~~, 3~~, Fig.~~ and S7A~~-~~–I, Pipeline 6. Amplicon.Rmd) within the microbiome analysis toolkit EasyMicrobiome (Liu et al., 2023). It enables various diversity analyses, including alpha diversity (Fig. S7A), rarefaction curve (Fig. S7B), clustering distance heatmap (Fig. S7C) and PCoA (Fig. S7D), NMDS, LDA and PCA, taxonomic composition (Fig. S7E~~/~~ and S7F), difference analysis (Fig. S7G~~/~~ and S7H). Then, it can easily generate high-quality figures such as boxplots, scatter plots for diversity analysis, stacked bar plots, circlize plots, and map trees for taxonomic or functional composition. One of its notable features is its ability to finely adjust the presentation of figures, resulting in published-ready figures. Additionally, several tools within the amplicon package are available for microbiome data transformation, facilitating subsequent analysis using tools such as LEfSe and STAMP. However, at the current version, the amplicon package does not provide some functions for network analysis, analysis of microbiome-environment interactions, and analysis of community formation processes. The authors provide some scripts in EasyAmplicon pipeline to do this, mentioned in the published paper plan to finish these functions in the future.

The best practice for microbiome data analysis in R

The abundance of R packages can hinder microbiome researchers from efficiently selecting appropriate R packages for microbiome-related analyses. Therefore, we organized and selected efficient, commonly used, and user-friendly functions for microbiome data analysis in six categories (Fig. S8): 1) diversity analysis (Figs. S9A~~-~~–I~~; Figs.~~ and S10A~~-~~–E), 2) difference analysis (Figs. S10F~~-~~–I~~,; Figs. -~~ S11A-and S11B), 3) biomarker identification (Fig~~s~~. S11C- and S11D), 4) correlation and network analysis (Figs. S11E-I), 5) functional prediction, 6 other microbiome analyses (Fig~~s~~. S12A~~-~~–I). All the script can be found in the file Pipeline.BestPractice.Rmd. This led to develop a better microbiome data analysis pipeline.

In this pipeline, we used the amplicon package for alpha diversity rarefaction curve (Figs. 4A~~; Fig.~~ and S9A) and PCoA analysis (Figs. 4B~~; Fig.~~ and S9B), ggplot2 package for visualization of microbial community composition, ggClusterNet for constructing Venn network (Chen et al., 2021) (Fig. 4C), ggtree and ggtrextre for building evolutionary trees (Fig. 4D), and LEfSe for generating cladograms (Fig. 4E). We employed the stst4, ggplot2, and cowplot packages for difference analysis and generated STAMP plots (Fig. 4F), used edgeR for difference analysis and visualized in Manhattan plots (Fig. 4G), and applied DESep2 for difference analysis and generated multi-group volcano plots (Fig. 4H). We also used the el071, caret, randomforest, ROC packages for various machine learning analyses and generated microbiome weighted plots (Fig. 4I). Furthermore, we used ggClusterNet for microbiome network analysis (Fig. 4J), constructed network graphs and combined plots to explore the associations between environmental factors and microbiome communities (Fig. 4K). Finally, we used the FEAST package to perform community source tracking analysis and constructed pie charts (Fig. 4L). Other analyses included stacked bar charts of microbial community composition (Figs. S9E/H), chord diagrams (Fig. S10A), Venn diagrams (Fig. S10C), Upset diagrams (Fig. S10D), difference analysis volcano plots (Fig. S10F), functional prediction etc.

For Peer Review

Perspective and conclusions

In the past ten years, the R language and numerous R packages have played an important role in the microbiome data analysis. R language is easy to use and get started. It has attracted many researchers to learn about it. However, there are still some contradictions between supply and demand in the microbiome data analysis. For example, it is often difficult to support multi-threading under the Windows system; secondly, the speed of many R packages running is relatively slow, although some R packages are written in other languages as supplements; third, the application in microbiome still needs further development. For instance, there is a shortage of

packages that allow for the exploration of time-series-based microbial compositions, as well as more robust interactive packages for analyzing complex microbial data. Furthermore, ggplot2 lacks the capability to create complex and combined figures, which fails to meet the visualization requirements for relationships between multiple intricate indicators with microbial community data. Therefore, developing new R packages that are more suitable for drawing complex figures and composite figures would be necessary for microbiome data.

With the development of sequencing technology, data analysis methods have advanced along with the development of R packages contributed to the field of microbiome. These R packages range from classic R packages such as vegan, which has been cited more than 10,000 times, to integrated R packages such as phyloseq, which contain many functions in one package and set a unified data processing framework. These R packages have been able to implement most of the functions of microbiome analysis, from microbial diversity, difference, biomarker identification, correlation and network, phylogenetic analysis, etc. However, these R packages have some redundant functions; for example, phyloseq, microbiome, and others can do microbial diversity analysis. The difference is only in the visualization method and scheme. A similar situation has always existed in microbiome analysis R packages, so we hope that in future developments we will try to de-redundantly use the same part of the content or similar content to highlight the advantages of R packages.

Although these R packages can conduct a lot of functions, they don't well enough in some specific analyses, for example, alpha and beta diversity analysis, and the outgoing graphs often not add difference detection results to visualize the differences from the figures. In addition, there are still some contents that can continue to be developed, such as applying more machine learning methods to microbiome data and its learning method, model, and important variable evaluation. Secondly, metagenomes are becoming more widely used, and the support of species and functional annotation results based on Kraken (Wood and Salzberg, 2014), MEGAN (Huson et al., 2007), MetaPhlAn2 (Truong et al., 2015), HUMAnN2 (Franzosa et al., 2018), eggNOG-mapper (Huerta-Cepas et al., 2017), etc. is becoming more and more important, and these make the data processed by R rise from megabyte (M) to gigabyte (G). Therefore, Faster data processing R packages should be used to the microbiome data analysis process, such as data.table, fst, tidyfst etc.

The use of appropriate data structures can accelerate the microbiome data processing. At first, we used S4 class objects for microbiome data encapsulation, which can complete a variety of analyses comprehensively and efficiently. The emergence of S6 class objects and other objects has greatly impacted microbiome data processing and largely facilitates it. With the development of the tidy family of R languages, tidy-based data structures have recently emerged for microbiome data mining. For example, the MicrobiotaProcess package (Xu et al., 2023). This structure is more suitable for microbiome data mining, machine learning modeling, and other analyses, which can more easily extract the influence of experimental design, time, space, and other factors on microbiome data in analysis, to discover the deep-seated patterns. We expect the R language to make microbiome analysis more efficient and

For Peer Review

help everyone discover more about its role in humans, animals, plants, and the environment, and use it for our benefit to make the world a better place.

Supplementary information

The online version contains Figure S1–12, and Table S1.

Declarations

Funding

This study was financially supported by the Agricultural Science and Technology Innovation Program (CAAS-ZDRW202308), the Natural Science Foundation of China (42277297, 42090060, U21A20182), Jiangsu Funding Program for Excellent Postdoctoral Talent (2022ZB325), Scientific and technology innovation project of China Academy of Chinese Medical Sciences (C12021A04115), the Fundamental Research Funds for the Central public welfare research institutes (ZZ13-YQ-095).

Competing interests

The authors declare no competing interests related to the content of this paper.

Ethics approval

Not applicable.

Consent to participate

Not applicable.

Consent for publication

All authors agree to publish.

Data availability

No new sequencing data generated by this project.

Code availability

All the demo data and scripts are available in GitHub: https://github.com/taowenmicro/EasyMicrobiomeR.

Author contributions

J.Y. and Y.L. conceived and supervised the project; T.W. and G.N. implement this project and wrote the paper; Y.L., T.C., and Q.S provided critical comments and revised the paper.

Acknowledgments

We thank all the people star and fork this project in GitHub and feedback the useful

For Peer Review

comments.

Abbreviations

ASV, an amplicon sequence variant; CCA, canonical correspondence analysis; CSS, cumulative-sum scaling; DCA, decision curve analysis; GO, gene ontology; GSEA, gene set enrichment analysis; GSVA, gene set variation analysis; KEGG, kyoto encyclopedia of genes and genomes; LDA, linear discriminant analysis; LEfSe, linear discriminant analysis effect size; NMDS, non-metric multidimensional scaling; OTU, operational taxonomic unit; PCA, principal components analysis; PCoA, principal coordinate analysis; RLE, relative log expression; ROC, receiver operating characteristic curve; TMM, trimmed mean of M-values; UQ, upper quartile; MED, median of ratios method.

References

For Peer Review

Amir A, McDonald D, Navas-Molina JA *et al.* Deblur Rapidly Resolves Single-Nucleotide Community Sequence Patterns. *MSystems* 2017;**2**:e00191-00116.

Aßhauer KP, Wemheuer B, Daniel R *et al.* Tax4Fun: predicting functional profiles from metagenomic 16S rRNA data. *Bioinformatics* 2015;**31**:2882-2884.

Barnett DJ, Arts IC, Penders J. microViz: an R package for microbiome data visualization and statistics. *Journal of Open Source Software* 2021;**6**:3201.

Bolyen E, Rideout JR, Dillon MR *et al.* Reproducible, interactive, scalable and extensible microbiome data science using QIIME 2. *Nature Biotechnology* 2019;**37**:852-857.

Callahan BJ, McMurdie PJ, Rosen MJ *et al.* DADA2: High-resolution sample inference from Illumina amplicon data. *Nature Methods* 2016;**13**:581-583.

Caporaso JG, Kuczynski J, Stombaugh J *et al.* QIIME allows analysis of high-throughput community sequencing data. *Nature Methods* 2010;**7**:335-336.

Carrión VJ, Perez-Jaramillo J, Cordovez V *et al.* Pathogen-induced activation of disease-suppressive functions in the endophytic root microbiome. *Science* 2019;**366**:606-612.

Chen H, Boutros PC. VennDiagram: a package for the generation of highly-customizable Venn and Euler diagrams in R. *BMC bioinformatics* 2011;**12**:1-7.

Chen T, Zhang H, Liu Y *et al.* EVenn: Easy to create repeatable and editable Venn diagrams and Venn networks online. *Journal of Genetics and Genomics* 2021;**48**:863-866.

Chen Y, Li J, Zhang Y *et al.* Parallel-Meta Suite: Interactive and rapid microbiome data analysis on multiple platforms. *iMeta* 2022;**1**:e1.

Chong J, Liu P, Zhou G *et al.* Using MicrobiomeAnalyst for comprehensive statistical, functional, and meta-analysis of microbiome data. *Nature Protocols* 2020;**15**:799-821.

Conway JR, Lex A, Gehlenborg N. UpSetR: an R package for the visualization of intersecting sets and their properties. *Bioinformatics* 2017;**33**:2938-2940.

Dimitriadou E, Hornik K, Leisch F *et al.* Misc functions of the Department of Statistics (e1071), TU Wien. *R package* 2008;**1**:5-24.

Dray S, Blanchet G, Borcard D *et al.* Package 'adespatial'. 2018;**2018**:3-8.

Dray S, Dufour A-B. The ade4 package: implementing the duality diagram for ecologists. *Journal of statistical software* 2007;**22**:1-20.

Edgar RC. Search and clustering orders of magnitude faster than BLAST. *Bioinformatics*

2010;**26**:2460-2461.
Edgar RC, Flyvbjerg H. Error filtering, pair assembly and error correction for next-generation sequencing reads. *Bioinformatics* 2015;**31**:3476-3482.
Fisher RA. The use of multiple measurements in taxonomic problems. *Annals of eugenics* 1936;**7**:179-188.
Franzosa EA, McIver LJ, Rahnavard G *et al.* Species-level functional profiling of metagenomes and metatranscriptomes. *Nature methods* 2018;**15**:962-968.
Gu Z. Complex heatmap visualization. *iMeta* 2022;**1**:e43.
Gu Z, Gu L, Eils R *et al.* circlize implements and enhances circular visualization in R. *Bioinformatics* 2014;**30**:2811-2812.
Hamilton NE, Ferry M. ggtern: Ternary diagrams using ggplot2. *Journal of Statistical Software* 2018;**87**:1-17.
Harrell Jr FE, Harrell Jr MFE. Package 'hmisc'. *CRAN2018* 2019;**2019**:235-236.
Hofner B, Mayr A, Robinzonov N *et al.* Model-based boosting in R: a hands-on tutorial using the R package mboost. *Computational statistics* 2014;**29**:3-35.
Huerta-Cepas J, Forslund K, Coelho LP *et al.* Fast genome-wide functional annotation through orthology assignment by eggNOG-mapper. *Molecular biology evolution* 2017;**34**:2115-2122.
Huson DH, Auch AF, Qi J *et al.* MEGAN analysis of metagenomic data. *Genome Res* 2007;**17**:377-386.
Ihaka R, Gentleman R. R: A Language for Data Analysis and Graphics. *Journal of Computational and Graphical Statistics* 1996;**5**:299-314.
Kembel SW, Cowan PD, Helmus MR *et al.* Picante: R tools for integrating phylogenies and ecology. *Bioinformatics* 2010;**26**:1463-1464.
Knights D, Kuczynski J, Charlson ES *et al.* Bayesian community-wide culture-independent microbial source tracking. *Nature methods* 2011;**8**:761-763.
Kuhn M. Building Predictive Models in R Using the caret Package. *Journal of Statistical Software* 2008;**28**:1-26.
Kurtz ZD, Müller CL, Miraldi ER *et al.* Sparse and Compositionally Robust Inference of Microbial Ecological Networks. *PLOS Computational Biology* 2015;**11**:e1004226.
Langfelder P, Horvath S. WGCNA: an R package for weighted correlation network analysis. *BMC bioinformatics* 2008;**9**:1-13.
Li W, Wang L, Li X *et al.* Sequence-based Functional Metagenomics Reveals Novel Natural Diversity of Functioning CopA in Environmental Microbiomes. *Genomics Proteomics Bioinformatics* 2022;**20**:1-12.
Liaw A, Wiener M. Classification and regression by randomForest. *R news* 2002;**2**:18-22.
Lin H, Peddada SD. Analysis of microbial compositions: a review of normalization and differential abundance analysis. *NPJ biofilms and microbiomes* 2020;**6**:1-13.
Liu C, Cui Y, Li X *et al.* microeco: an R package for data mining in microbial community ecology. *FEMS Microbiology Ecology* 2020;**97**:fiaa255.
Liu Y, Qin Y, Chen T *et al.* A practical guide to amplicon and metagenomic analysis of microbiome data. *Protein & cell* 2021;**12**:315-330.
Liu YX, Chen L, Ma T *et al.* EasyAmplicon: An easy-to-use, open-source, reproducible, and community-based pipeline for amplicon data analysis in microbiome research. *iMeta* 2023;**2**:e83.
Louca S, Parfrey LW, Doebeli M. Decoupling function and taxonomy in the global ocean microbiome.

For Peer Review

Science 2016;**353**:1272-1277.

Love MI, Huber W, Anders S. Moderated estimation of fold change and dispersion for RNA-seq data with DESeq2. *Genome biology* 2014;**15**:1-21.

McMurdie PJ, Holmes S. phyloseq: an R package for reproducible interactive analysis and graphics of microbiome census data. *PloS one* 2013;**8**:e61217.

Metcalf JL, Xu ZZ, Weiss S *et al.* Microbial community assembly and metabolic function during mammalian corpse decomposition. *Science* 2016;**351**:158-162.

Nearing JT, Douglas GM, Hayes MG *et al.* Microbiome differential abundance methods produce different results across 38 datasets. *Nature Communications* 2022;**13**:342.

Nguyen NH, Song Z, Bates ST *et al.* FUNGuild: An open annotation tool for parsing fungal community datasets by ecological guild. *Fungal Ecology* 2016;**20**:241-248.

Ning D, Yuan M, Wu L *et al.* A quantitative framework reveals ecological drivers of grassland microbial community assembly in response to warming. *Nature communications* 2020;**11**:4717.

Oksanen J, Kindt R, Legendre P *et al.* The vegan package. *Community ecology package* 2007;**10**:719.

Pages H, Aboyoun P, Gentleman R *et al.* Biostrings: String objects representing biological sequences, and matching algorithms. *R package version* 2016;**2**:10.18129.

Paoli L, Ruscheweyh H-J, Forneris CC *et al.* Biosynthetic potential of the global ocean microbiome. *Nature* 2022;**607**:111-118.

Pasolli E, Schiffer L, Manghi P *et al.* Accessible, curated metagenomic data through ExperimentHub. *Nature Methods* 2017;**14**:1023-1024.

Proctor LM, Creasy HH, Fettweis JM *et al.* The Integrative Human Microbiome Project. *Nature* 2019;**569**:641-648.

Revelle W, Revelle MW. Package 'psych'. *The comprehensive R archive network* 2015;**337**:338.

Ripley B, Venables B, Bates DM *et al.* Package 'mass'. *Cran r* 2013;**538**:113-120.

Robin X, Turck N, Hainard A *et al.* pROC: an open-source package for R and S+ to analyze and compare ROC curves. *BMC bioinformatics* 2011;**12**:1-8.

Robinson MD, McCarthy DJ, Smyth GK. edgeR: a Bioconductor package for differential expression analysis of digital gene expression data. *Bioinformatics* 2009;**26**:139-140.

Rognes T, Flouri T, Nichols B *et al.* VSEARCH: a versatile open source tool for metagenomics. *PeerJ* 2016;**4**:e2584.

Schloss PD, Westcott SL, Ryabin T *et al.* Introducing mothur: open-source, platform-independent, community-supported software for describing and comparing microbial communities. *Applied and environmental microbiology* 2009;**75**:7537-7541.

Shenhav L, Thompson M, Joseph TA *et al.* FEAST: fast expectation-maximization for microbial source tracking. *Nature Methods* 2019;**16**:627-632.

Si B, Liang Y, Zhao J *et al.* GGraph: An Efficient Structure-Aware Approach for Iterative Graph Processing. *IEEE Transactions on Big Data* 2022;**8**:1182-1194.

Stegen JC, Lin X, Fredrickson JK *et al.* Quantifying community assembly processes and identifying features that impose them. *The ISME journal* 2013;**7**:2069-2079.

Thompson LR, Sanders JG, McDonald D *et al.* A communal catalogue reveals Earth's multiscale microbial diversity. *Nature* 2017;**551**:457-463.

Truong DT, Franzosa EA, Tickle TL *et al.* MetaPhlAn2 for enhanced metagenomic taxonomic profiling. *Nature Methods* 2015;**12**:902-903.

Wemheuer F, Taylor JA, Daniel R *et al.* Tax4Fun2: prediction of habitat-specific functional profiles

and functional redundancy based on 16S rRNA gene sequences. *Environmental Microbiome* 2020;**15**:11.

Wen T, Xie P, Yang S *et al.* ggClusterNet: An R package for microbiome network analysis and modularity-based multiple network layouts. *iMeta* 2022;**1**:e32.

Wickham H. Reshaping Data with the reshape Package. *Journal of Statistical Software* 2007;**21**:1-20.

Wickham H. ggplot2. *Wiley interdisciplinary reviews: computational statistics* 2011a;**3**:180-185.

Wickham H. The Split-Apply-Combine Strategy for Data Analysis. *Journal of Statistical Software* 2011b;**40**:1-29.

Wirbel J, Zych K, Essex M *et al.* Microbiome meta-analysis and cross-disease comparison enabled by the SIAMCAT machine learning toolbox. *Genome Biology* 2021;**22**:93.

Wood DE, Salzberg SL. Kraken: ultrafast metagenomic sequence classification using exact alignments. *Genome biology* 2014;**15**:1-12.

Xu S, Li L, Luo X *et al.* Ggtree: A serialized data object for visualization of a phylogenetic tree and annotation data. *iMeta* 2022;**1**:e56.

Xu S, Zhan L, Tang W *et al.* MicrobiotaProcess: A comprehensive R package for deep mining microbiome. *The Innovation* 2023;**4**:100388.

Zhao Y, Federico A, Faits T *et al.* animalcules: interactive microbiome analytics and visualization in R. *Microbiome* 2021;**9**:1-16.

For Peer Review

Table 1. Comparison of the advantages and limitations of the six integrated R packages.

R package	Function	Advantages	Limitations
phyloseq	1. Diversity analysis including alpha-/-beta diversity, community composition, and phylogenetic tree analysis.<br>2. Network analysis.	1. Firstly utilize S4 class objects.<br>2. Possess lots of analysis functions based on phyloseq objects.<br>3. The network analysis process is simplified (Fig. S2E).<br>4. Ordinate analysis can be applied to arrange the order of samples and microbes on heatmap rows and columns (Fig. S2F).<br>5. Combine evolutionary trees with microbial abundance to display species richness (Fig. S2G).<br>6. Offer over 30 distance algorithms.	1. Introduction to phyloseq objects can be challenging for beginners.<br>2. Statistical tests, including diversity tests and community/feature-level microbial difference analysis, are not well integrated into community analysis.<br>3. Network analysis lacks test, attribute calculation.
microbiomeMicrobiome	1. Diversity analysis only including alpha–/–beta diversity, community composition.	1. The alpha diversity index is abundance.<br>2. The t-SNE and CAP ordination algorithms.<br>3. The stacked bar chart for community composition analysis can be sorted by specified microbial features (Fig. S3C).<br>4. Visualization of individual microbes (Fig. S3D).	1. The t-SNE and CAP ordination analyses frequently encounter errors.<br>2. The statistical tests, including diversity tests, community and feature-level differences tests is not ideal.
Microbiome AnalystR	1. Diversity analysis including alpha/beta diversity, community composition, and phylogenetic tree analysis.<br>2. Difference analysis.<br>3. Biomarker identification.	1. Various functions ranging from data cleaning to visualization.<br>2. Multiple algorithms to correct sequencing errors, leading more accurate evaluation of abundance.<br>3. Machine learning can be utilized to extract feature variables (Fig. S4H).<br>4. Difference analysis using multiple methods, such as	1. Difficulties in installing R packages with dependencies.<br>2. Some functions may not work, including network analysis and difference analysis of relative abundance.<br>3. Insufficient explanation of parameters

For Peer Review

		LEfSe or metagenomeSeq.	and examples.
Animalcules	1. Diversity analysis.<br>2. Difference analysis and biomarker identification.	1. SummarizedExperiment package supported.<br>2. Interactively executed in R (Fig. S5A–J).<br>3. A 3D clustering plot can be generated.	1. Unable to save vector graphics and completed tables.<br>2. Insufficient functionality.
microeco	1. Diversity analysis.<br>2. Difference analysis.<br>3. Biomarker identification.<br>4. Network, correlation analysis with other indicators.<br>5. Functional prediction.	1. R6 class more expansibility than phyloseq objects.<br>2. Simple function calling.<br>3. Rich plots of diversity and difference analysis (Fig. S6A–H).<br>4. Unique correlation analysis of other indicators.<br>5. Network analysis functionality (Fig. S6K).<br>6. FAPROTAX and FUNGuild function prediction.	1. New data structures increase the cost of learning time.<br>2. So many functions and dependency caused frequent some malfunctioning.
EasyAmplicon	1. Diversity analysis.<br>2. Provide script for preparing STAMP, LEfSe, PICRUSt 1&2, BugBase, FAPROTAX, iTOL.<br>3. Provide slide tutorial for many analyses, such as QIIME 2.	1. It can be used in both command-line mode and interactive mode within RStudio.<br>2. It offers multiple visualization styles, allowing for easy generation of publication-quality figures (Fig. S7).<br>3. Its open-source code facilitates reproducible analysis and allows for personalized modifications.	1. Need using the most popular tools, STAMP, LEfSe, PICRUSt 1&2, BugBase, FAPROTAX, and iTOL.<br>2. Some functions need to be development.

For Peer Review

Figures & Legends

Figure 1. Microbial community data analysis workflow and related R packages.

(A) Overview of microbial community data analysis workflow. Core files are feature table (OTU), Taxonomy, sample metadata (Metadata), phylogenetic tree (Tree), and representative sequences (Rep.fa). (B) Detail of microbial community analysis workflow. First, the raw data can be processed by using USEARCH/VSEARCH, QIIME 2, DADA2 packages. Then, the important files are saved and used for downstream analysis in R language and RStudio software. Many microbial analysis methods rely on numerous R packages developed with R language. The font size in the word cloud represents the number of citations of R packages. (C) Commonly used R packages for data cleaning/manipulation and visualization. (D) Classification of R packages for six categories in microbial community analysis.

Figure 2. Introduction to the functions of integrated microbial analysis R packages.

Microbial community analysis can be divided into diversity analysis, difference analysis, biomarker identification, correlation and network analysis, functional prediction, and other microbial community analysis (community building/construction process, association analysis with other indicators).

Figure 3. Typical results of integrated microbial community analysis R packages and comparison of similar results.

Group the analysis results of multiple integrated R packages according to the major categories of microbial community analysis functions. Each main branch in the tree diagram represents a type of microbial community analysis, and there are a total of 10 main branches: feature diversity analysis including 1 alpha diversity analysis, 2 beta diversity analysis, 3 features (community taxonomic or functional) composition analysis, 4 evolutionary or taxonomic tree analysis; 5 difference analysis; 6 biomarker identification; 7 correlation and network analysis; 8 functional prediction; 9 community building/construction process analysis; 10 other analysis, such as association analysis with other indicators. Each leaf (circle) represents a style of the result displayed in the analysis, and the circle number around the outside of leaf represents the package number of the integrated R package that the analysis result comes from.

Figure 4. Examples of the best practice results of microbial community analysis in R language.

The selected results include rarefaction curve (A), principal coordinate analysis scatter plot (B), Venn network graph (C), evolutionary tree (D), LEfSe cladogram (E), difference analysis extended error bar plot in STAMP style (F), difference analysis Manhattan plot (G), difference analysis multi-group volcano plot (H), biomarker selection ring-column chart (I), network graph (J), correlation connection combination graph (K), source tracing analysis pie chart (L).

For Peer Review

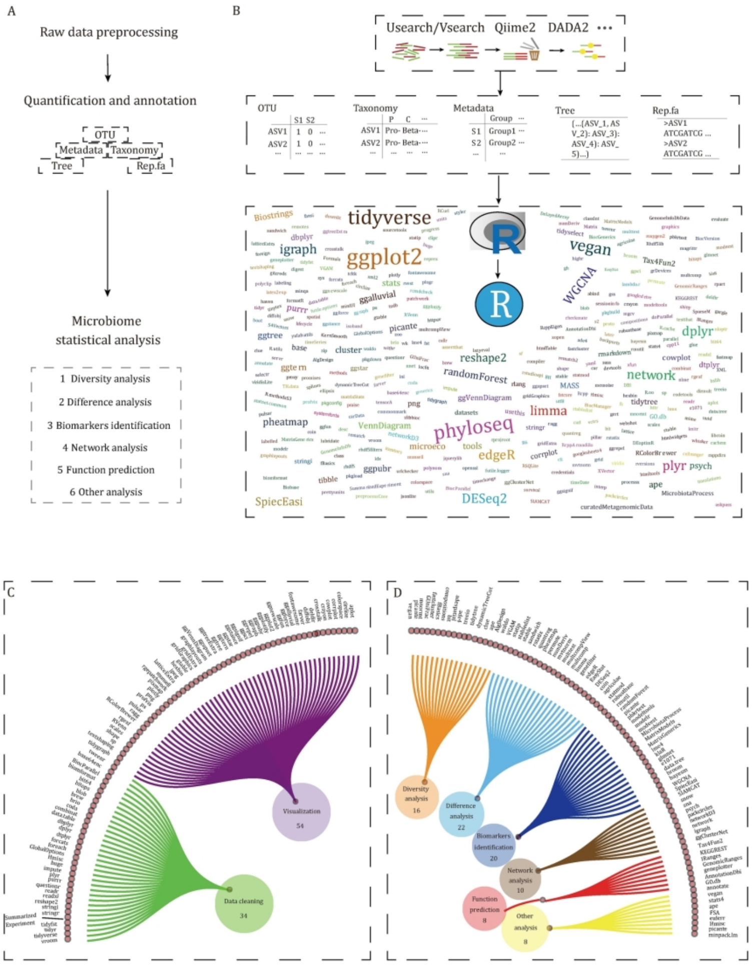

173x249mm (300 x 300 DPI)

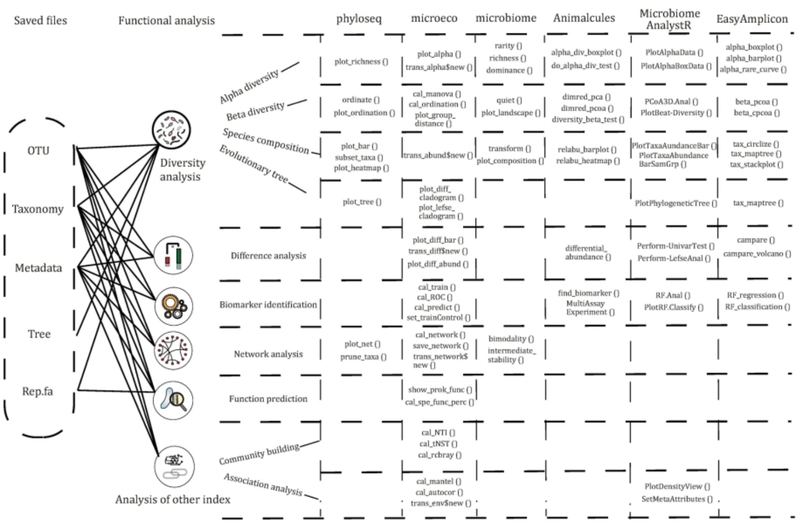

173x249mm (300 x 300 DPI)

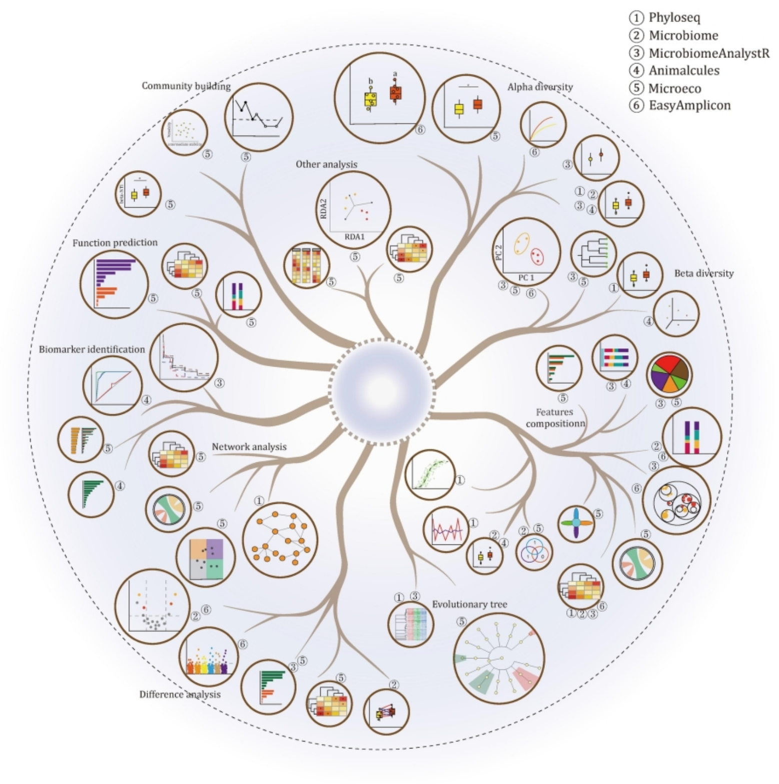

173x249mm (300 x 300 DPI)

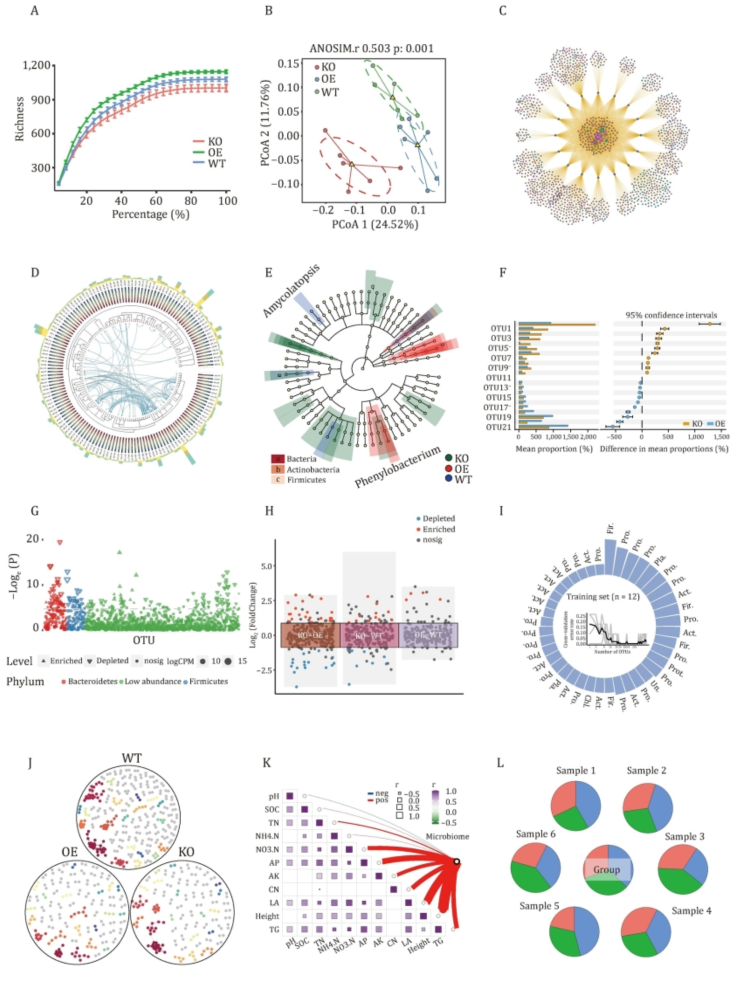

173x249mm (300 x 300 DPI)

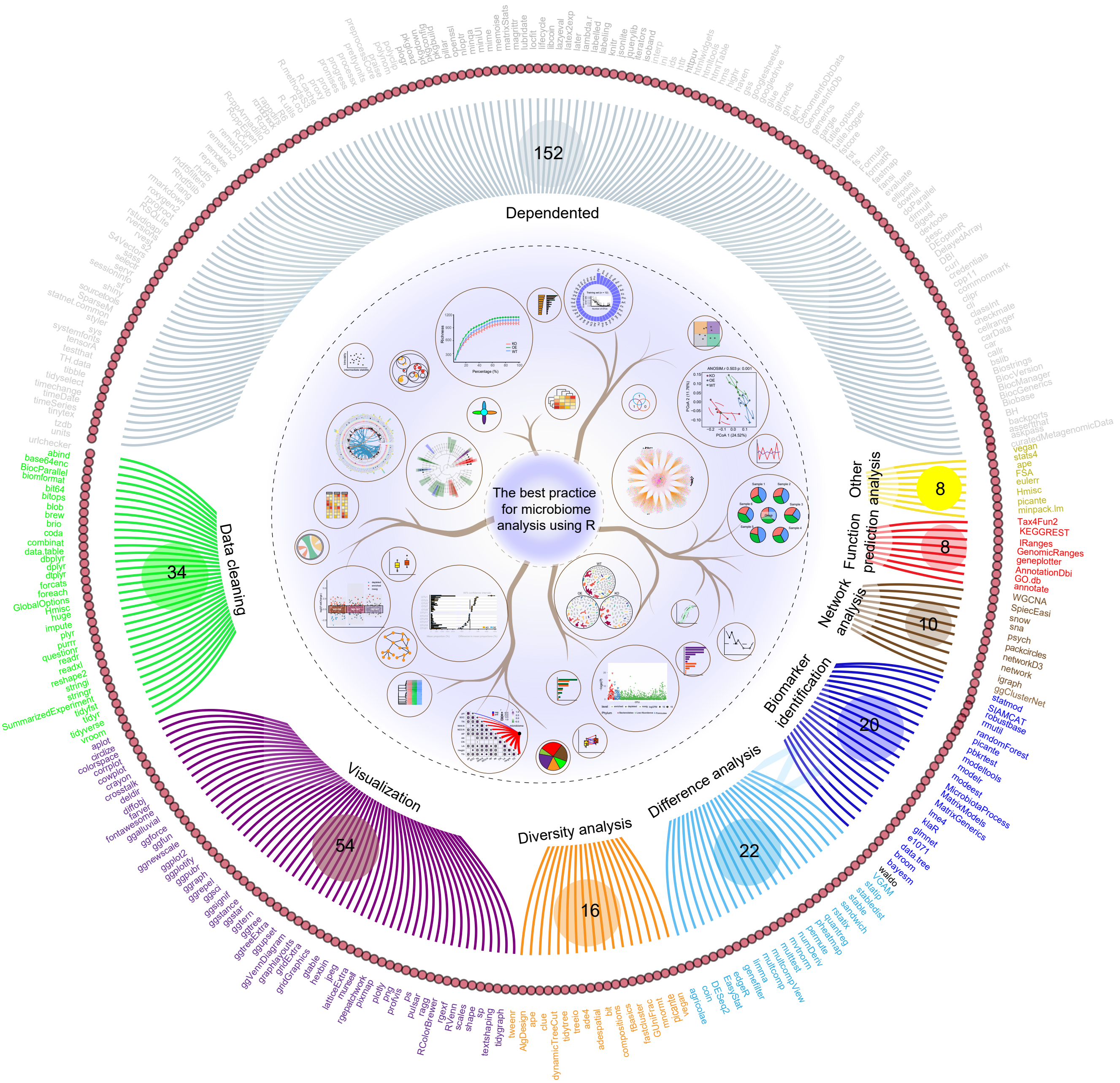
152
Dependented
The best practice for microbiome analysis using R
Data cleaning
34
Visualization
54
Diversity analysis
16
Difference analysis
22
Biomarker identification
20
Network analysis
10
Function prediction
8
Other analysis
8
